# The Role of Parathyroid Hormone-Related Protein (PTHrP) in Osteoblast Response to Microgravity: Mechanistic Implications for Osteoporosis Development

**DOI:** 10.1371/journal.pone.0160034

**Published:** 2016-07-27

**Authors:** Anne Camirand, David Goltzman, Ajay Gupta, Mohammadi Kaouass, Dibyendu Panda, Andrew Karaplis

**Affiliations:** 1 McGill University Health Centre, Montréal, Québec, Canada; 2 Department of Oncology, McGill University, Montreal, Québec, Canada; 3 Department of Biology, Université Sainte-Anne, Pointe-de-l’Eglise, Nova Scotia, Canada; 4 Lady Davis Institute, Jewish General Hospital, Montréal, Québec, Canada; Faculté de médecine de Nantes, FRANCE

## Abstract

Prolonged skeletal unloading through bedrest results in bone loss similar to that observed in elderly osteoporotic patients, but with an accelerated timeframe. This rapid effect on weight-bearing bones is also observed in astronauts who can lose up to 2% of their bone mass per month spent in Space. Despite the important implications for Spaceflight travelers and bedridden patients, the exact mechanisms involved in disuse osteoporosis have not been elucidated. Parathyroid hormone-related protein (PTHrP) regulates many physiological processes including skeletal development, and has been proposed as a mechanosensor. To investigate the role of PTHrP in microgravity-induced bone loss, trabecular and calvarial osteoblasts (TOs and COs) from *Pthrp* ^+/+^ and ^-/-^ mice were subjected to actual Spaceflight for 6 days (Foton M3 satellite). *Pthrp* ^+/+^, ^+/-^ and ^-/-^ osteoblasts were also exposed to simulated microgravity for periods varying from 6 days to 6 weeks. While COs displayed little change in viability in 0*g*, viability of all TOs rapidly decreased in inverse proportion to PTHrP expression levels. Furthermore, *Pthrp*^+/+^ TOs displayed a sharp viability decline after 2 weeks at 0*g*. Microarray analysis of *Pthrp*^+/+^ TOs after 6 days in simulated 0*g* revealed expression changes in genes encoding prolactins, apoptosis/survival molecules, bone metabolism and extra-cellular matrix composition proteins, chemokines, insulin-like growth factor family members and Wnt-related signalling molecules. 88% of 0*g*-induced expression changes in *Pthrp*^*+/+*^ cells overlapped those caused by *Pthrp* ablation in normal gravity, and pulsatile treatment with PTHrP_1-36_ not only reversed a large proportion of 0*g*-induced effects in *Pthrp*^*+/+*^ TOs but maintained viability over 6-week exposure to microgravity. Our results confirm PTHrP efficacy as an anabolic agent to prevent microgravity-induced cell death in TOs.

## Introduction

Bone loss due to osteoporosis (OP) is the most common cause of fractures among the elderly. Age-related OP elicits fractures with minimal trauma in up to 40% of women and 20% of men over the age of fifty and is a major health and fiscal burden [1, 2]. OP can also develop in younger individuals as a consequence of premature menopause or skeletal unloading. In the absence of gravity load on weight-bearing bones, the equilibrium between osteoblastic bone formation and osteoclastic bone resorption is uncoupled and the balance is shifted towards resorption, resulting in bone loss. Skeletal disuse can occur after prolonged bedrest on Earth but is also encountered in the weightless conditions of Space [3]. During orbital flight, astronauts are exposed to gravity forces below the physiological detection threshold (≤ 10^-5^*g*). Even a short-duration exposure to microgravity results in profound metabolic changes, and the lack of mechanical loading causes rapid demineralization in bones of the lower body leading to development of a disuse osteoporotic-like phenotype [4]. Microgravity can cause a remarkable loss of up to 2% of load-bearing bone mineral density (BMD) per month spent in Space, compared to an average 1% per year observed on Earth (starting from approximately 24–30 years of age when peak bone mass is attained) [5]. Microgravity-induced bone loss shows no sign of stabilization while at 0*g* and displays a highly-variable speed of skeletal recovery upon return to Earth [6–10]. The causes of this rapidly-progressing syndrome have not been elucidated, and only partially-effective countermeasures have been developed [11].

Skeletal loss has severe implications for long-time inhabitants of the International Space Station and will be a hindrance to spatial exploration since up to one half of bone mass could be lost during a three-year trip to Mars, resulting in mission-compromising fractures as well as complications from renal stones caused by skeleton-released calcium [5, 12–14]. The mechanosensors which control bone loss during unloading are not known, and the striking difference in microgravity-induced mineral loss between bone derived from endochondral formation (eg. trabecular bone) and bone derived from membraneous formation (calvarial bone) has also not been fully analysed. Elucidation of the causes of skeletal loss at 0*g* is further complicated by the fact that the two main cellular components regulating bone turnover, the osteoblasts and osteoclasts, are differently affected by microgravity. Osteoblasts exposed to 0*g* undergo decreases in cellular integrity with modified microtubule structure, focal adhesions and increasingly fragmented nuclei, while osteoclasts present higher numbers of discrete resorption pits and increased cellular activity [15]. Spaceflight countermeasures to bone loss include exercise and resistive training, artificial musculoskeletal loading, electrical stimulation and vibration treatment, as well as dietary additions of vitamin D, K, calcium and various drugs [16]. While physical activity in Space helps to counter muscle atrophy, it does not prevent bone loss and none of these measures fully compensate for skeletal degradation [10, 13, 14]. In long-duration bedrest studies which mimic microgravity’s physiological effects, treatment of male subjects with the anti-resorptive bisphosphonate drugs alendronate or pamidronate (osteoclast inhibitors) prevented some, but not all, bone loss [17, 18]. Furthermore, skeletal bone formed during bisphosphonate administration does not display the mechanical integrity of normal bone [6, 19] and high doses of anti-resorptive drugs may inhibit formation in unloaded bone [20]. Consequently, an alternative to be investigated is the use of anabolic substances such as parathyroid hormone (PTH) and parathyroid hormone-related hormone (PTHrP) which promote bone formation rather than inhibit resorption [21, 22].

PTHrP is a secreted factor expressed in almost all normal fetal and adult tissues. It acts as an autocrine, paracrine or intracrine factor in a wide range of developmental and physiological processes, including skeletal development and growth [23, 24]. The 13 N-terminal amino acids of PTHrP are highly-homologous to those of PTH, a characteristic that allows PTHrP to activate their common type 1 PTH/PTHrP receptor. PTH and PTHrP differ in the remainder of their sequence, a fact which accounts at least in part for the distinct properties of the two molecules [23, 24]. The major role played by PTHrP in bone development was made evident through *Pthrp* gene deletion which caused profound abnormalities in the development of the cartilaginous growth plate [25, 26], a phenotype not seen in mice with *PTH* deletion [27]. Heterozygous mice with only one null *Pthrp* allele (*Pthrp* ^*+/-*^) developed premature OP due to decreased bone formation linked to enhanced apoptosis of osteogenic cells [28]. Knock-out mice with a selective *Pthrp* deletion targeting only the osteoblasts further recapitulated this severe osteoporotic phenotype and importantly, exogenous application of PTH or PTHrP prevented apoptosis and associated bone loss [29]. PTH_1-34_ (teriparatide, Forteo^™^) is currently the only approved anabolic drug for treatment of osteoporosis but may cause bone resorption, hypercalcemia, nausea, muscle cramps and other adverse side-effects. In contrast, a study conducted on post-menopausal women showed that the _1–36_ N-terminal peptide of PTHrP did not activate bone resorption (based on bone marker responses, although changes in BMD were not measured) and acted as a pure anabolic agent while causing none of the adverse effects observed with teriparatide [30]. Although intermittent treatment with PTHrP causes an anabolic response, continuous treatment causes paradoxical downregulation of osteogenic genes resulting in skeletal catabolism [31, 32].

Decreased expression levels of PTHrP have been reported in rat long bones and cultured osteoblasts following exposure to microgravity [33], and loss of PTHrP may underlie at least part of the pathophysiology of microgravity-induced OP. We consequently tested the hypothesis that PTHrP could counter actual or simulated microgravity-induced deleterious effects in osteoblasts, and we investigated a timeline of viability for osteoblasts in weightlessness which would predispose astronauts to impaired bone formation. We also examined the efficacy of exogenous PTHrP_1-36_ peptide administration in preventing osteoblast viability loss in microgravity conditions. For these studies, we used trabecular and calvarial osteoblasts isolated one day before birth from bones of *Pthrp* ^+/+^, ^+/-^, or ^-/-^ mice. The cells in adherent conditions were subjected to Spaceflight aboard the Foton M3 satellite or to simulated microgravity in a Synthecon rotary cell culture system (RCCS) for 6-day experiments, with or without intermittent PTHrP _1–36_ treatment. We observed that PTHrP expression confers resistance to microgravity-induced cell death, that a very large proportion of the genes affected by microgravity is common to those affected by *Pthrp* gene deletion, and that intermittent treatment with the PTHrP_1-36_ peptide reverses the 0*g* effects on TO viability.

## Materials and Methods

### Animal Ethics Statement

All experiments were carried out in compliance with regulations of the McGill University Institutional Animal Care Committee (Animal permit: 5210) which specifically approved this study.

The mice were housed in conventional (non-barrier) animal facilities at 21°C with light-dark cycles of 12 hours, and were fed Bacon Softies^™^ soft pellets (Bio Serv) and water from drinking bottles *ad libitum*.

### Animals

Mice with various levels of *Pthrp* gene ablation (wild-type *Pthrp* ^+/+^, heterozygous *Pthrp* ^+/-^, and homozygous *Pthrp* ^-/-^) in a C57BL/6 background [26] were used to obtain osteoblasts. Because homozygous animals (*Pthrp* ^-/-^) exhibit perinatal lethality due to skeletal and pulmonary problems [26], osteoblasts were collected from fetal mice of all types one day before birth. Briefly, *Pthrp* ^+/-^ males were bred with *Pthrp* ^+/-^ females, pregnant females were sacrificed by cervical dislocation one day before partum and the fetal mice collected and sacrificed by decapitation. The animals were immediately surface-sterilized in 70% ethanol, tail samples were preserved for genotyping, and femoral and cranium bones extracted immediately to obtain trabecular and calvarial osteoblast cells respectively.

### Preparation of osteoblasts and cell culture

The trabecular and calvarial cells were obtained by a method modified after [34]. Briefly, calvarial (cranium, derived from membraneous bone formation) and trabecular (femur, derived from endochondral bone formation) bones were dissected from the animals and predigested in 6-well culture plates for 15 min at 37°C with gentle rotation in digestion medium (TCDA) made immediately before use: trypsin 0.05%, type I collagenase (2 mg/ml, Gibco), DNAse I (0.3 mg/ml, Sigma), penicillin streptomycin (100 U/ml). Bones were transferred to new 6-well plates with fresh TCDA, chopped into small slivers, particles were transferred to a third plate with TCDA and incubated at 37°C 15 min with rotation. Finally, the bone fragments were transferred to a fourth plate containing MAF medium (α-MEM with 15% FBS, penicillin/streptomycin 100 U/ml, fungizone 0.25 ug/ml) and incubated without rotation at 37°C in a humidified cell incubator (5% CO_2_). Only the cells emerging from the bone fragments at this step were kept for experimental purposes. The MAF medium was changed every two days and cells were transferred to 6-well plates when confluent. Aliquots were incubated with AGD differenciation medium (α-MEM 15% FBS, penicillin/streptomycin 100U/ml, fungizone 0.25 ug/ml, ascorbic acid 50 ug/ml, glycerophosphate 10 mM and dexamethasone 0,1 uM final) [35] or MAF as control to test for differenciation markers. The osteoblast primary cultures were filtered through sterile nylon cell strainers (100 microns, BD Biosciences), passaged twice, and 500,000 cells (minimum) were frozen per vial in 90% FBS, 10% DMSO using cryojars (ethanol/ dry ice) for 4 hours at -80°C. The cell vials were transferred to liquid nitrogen storage after 2 days. All culture reagents were from Wisent.

### Genotyping

Tail segments were incubated in lysis buffer (100 mM Tris-HCl 8.0, 5 mM EDTA 8.0, 0.2% SDS, 200 mM NaCl, 0.2 mg/ml proteinase K) at 55°C for 16 hours. Genomic DNA was extracted with phenol/CHCl_3_, ethanol-precipitated and resuspended in TE buffer. Aliquots were digested with PvuII, separated by agarose electrophoresis, and blots hybridized with a PTHrP-specific probe [26].

### Culture for Space mission bioreactors

**Fig 1:**
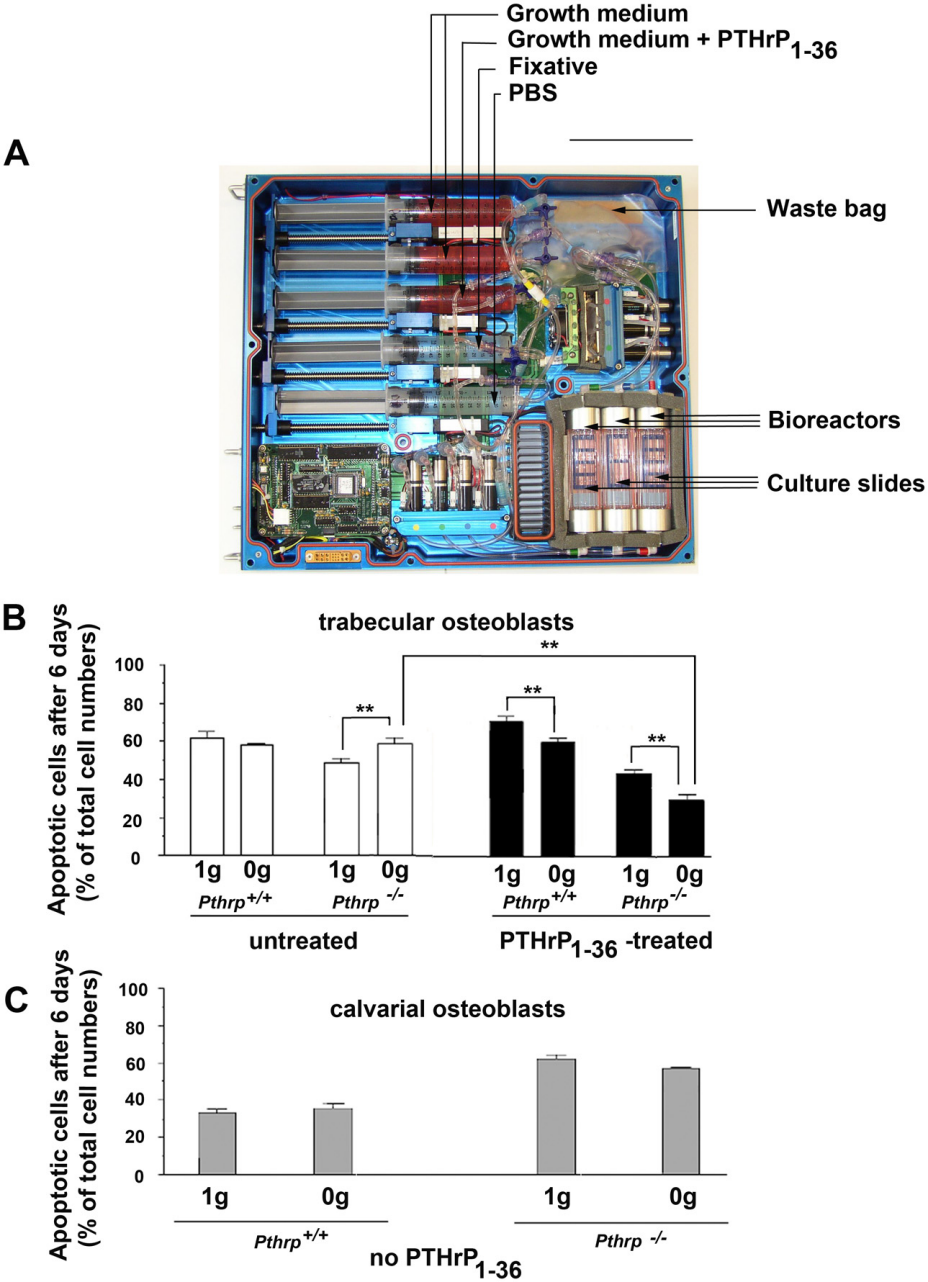
Endogenous PTHrP protects trabecular osteoblasts from Space microgravity-induced apoptosis. (6-day experiments). **(A)** Automated flight-ready tray for conducting cell culture experiments aboard Foton M3 satellite. Osteoblasts were attached onto CC2 culture slides inside three bioreactors (lower right) in a sealed pathway. **(B)** Proportion of apoptotic cells in trabecular osteoblasts in bioreactors after 6 days in Space microgravity (0*g*) or in normal Earth gravity (1*g*). ** p < 0.01. White bars: untreated; black bars: 2-h daily treatment with PTHrP_1-36_ 10^-8^M. **(C)** Proportion of apoptotic cells in calvarial osteoblasts in bioreactors after 6 days in Space microgravity (0*g*) or in normal Earth gravity (1*g*). No PTHrP_1-36_ treatment was conducted on calvarial cells. Scale bar A: 15 cm.

The available surface on culture slides in the Foton M3 mission biomodules was extremely limited, and allowed testing of the *Pthrp* ^+/+^ and ^-/-^ genotypes only. Cultured primary osteoblasts of both genotypes from calvarial and trabecular origin were exposed to AGD differenciation medium for 4–5 days then trypsinised, counted and cells were attached at very high density (25,000 cells/cm^2^, or 6,500 cells/compartment in 4-compartment slides) to the growth surfaces of sterile CC2 glass slides (Nunc). These slides were handled in a completely sterile fashion and placed inside 10cm culture dishes in a normal culture incubator (5% CO_2_) for a 4-day pre-attachment period in AGD medium. The CC2 slides superstructures were removed under sterile conditions, the glass slides inserted into the sterile mission biomodules and connected to the feeding syringes and waste exhaust lines of the automated experimental trays (Fig 1A, CALM Technologies, Kingston, Canada). Two identical trays were sealed and powered by battery to maintain 37°C temperature and provide medium changes every 48 hours. One tray was kept at 1*g* at the European Space Research and Technology Centre (ESTEC, Noordwijk, The Netherlands) while the twin tray (for 0*g*) was transported over a period of 4 days to Baikonour and installed aboard the Roscosmos (Russian Federal Space Agency) Foton M3 satellite which was launched into orbital flight. (http://esamultimedia.esa.int/docs/foton/FOTON-M3_brochure.pdf).

### Experimental flight conditions

Experimental conditions were automatically started one day after reaching orbital flight. Cells were fed fresh AGD growth medium every day, or received a daily 2-hour treatment with 10^-8^M PTHrP peptide amino acids 1–36, (Bachem, Germany) in AGD, followed by a phosphate-buffered saline (PBS) rinse, then fresh AGD medium. Intermittent PTHrP_1-36_ treatment was used as it provides anabolic effects on bone whereas continuous treatment does not [36]. After 6 days of experimentation, the cells were fixed with 1% glutaraldehyde *in situ* on the slides within the 3 bioreactors, then stored in PBS at 15°C. After retrieval from the satellite, slides were stored in PBS at 4°C until assays. Experimental conditions were identical for both 0*g* and 1*g* trays (described in S1 Table).

### Apoptosis assay

Mission slides were analysed for apoptosis events using the single-stranded DNA (ssDNA) detection technique [37]. The glass slides with glutaraldehyde-fixed cells were heated for 20 minutes at 75°C in neat formamide, rinsed with H_2_O, blocked with 3% non-fat dry milk for 1h at room temperature, reacted with a monoclonal antibody to ssDNA (1:10) coupled with horseradish peroxidase (Chemicon), then colored with freshly-prepared diaminobenzidine (DAB) and counter-stained with hematoxylin. Fixed cells positive for DAB (i.e.ssDNA) were apoptotic at the time of in-flight fixation, cells stained with hematoxylin were viable at the same moment. 11 optical fields were counted on each slide and the proportion of apoptotic cells to non-apoptotic cells at the time of fixation was determined.

### Simulated microgravity in rotary cell culture system

**Fig 2:**
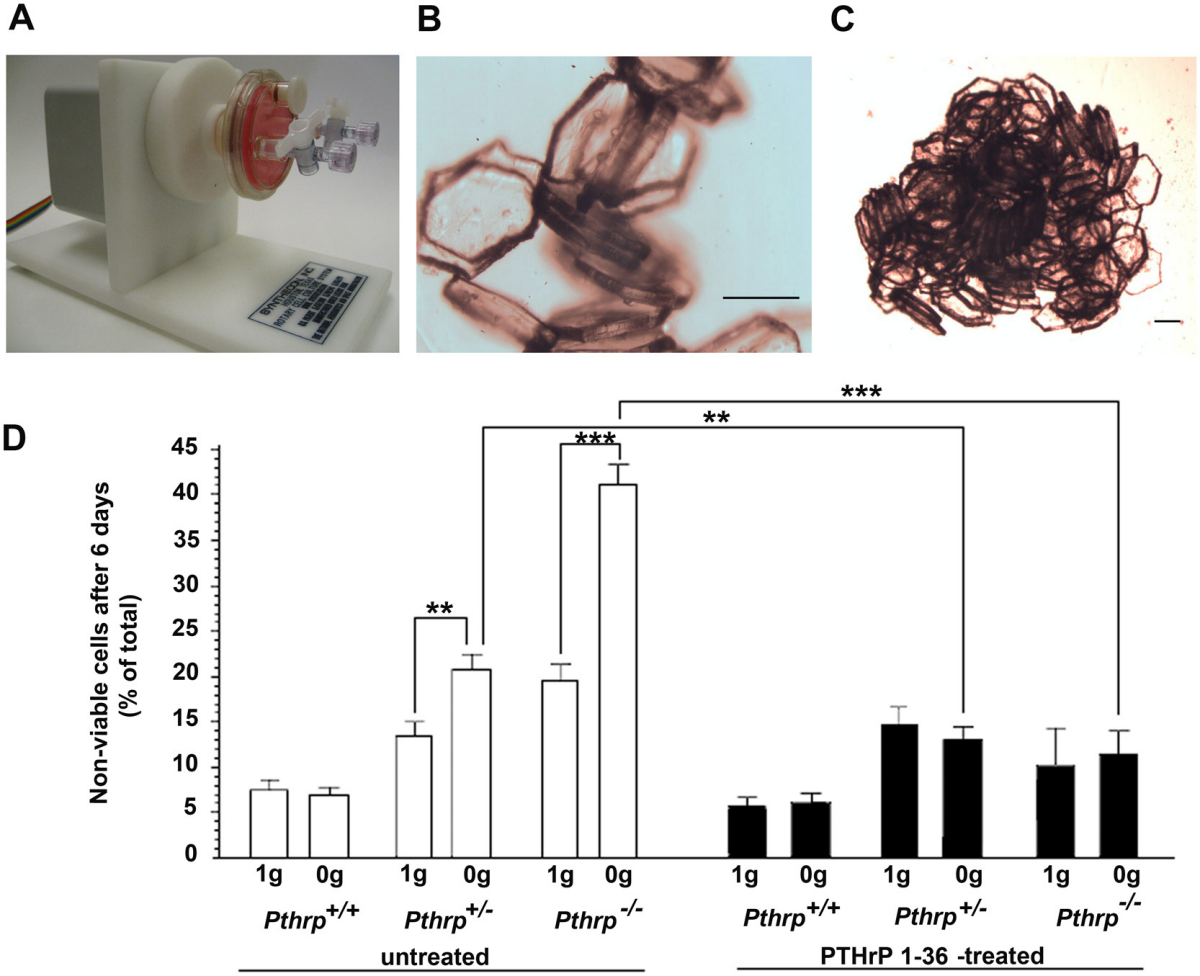
Allelic effect of endogenous PTHrP levels in simulated microgravity, and compensation by exogenous PTHrP _1–36_ (6-day experiments). **(A)** Microgravity simulation apparatus (RCCS) with one rotating culture unit (HARV). **(B)** MicroHex carriers with attached trabecular osteoblasts in culture. **(C)** Cell-induced MicroHex aggregate in culture (occurs for both trabecular and calvarial osteoblasts). **(D)** Trabecular osteoblast viability after 6 days on MicroHex carriers in normal gravity (1*g*) or in simulated microgravity (0*g*) (triplicate 6-day experiments). White bars: untreated; black bars: 2-h daily treatment with PTHrP_1-36_ 10^-8^M. ** p < 0.01, *** p < 0.001. Scale bars: B, C: 200 μm.

The flight data was completed, confirmed and expanded using the Synthecon Revolving Cell Culture System (RCCS) Earth-based microgravity simulator consisting of a horizontally-rotating axis connected to a sterile 10-ml disposable growth chamber called High Aspect Ratio Vessel (HARV) with a CO_2_-permeable membrane (Fig 2A, Synthecon, Houston, TX). The equipment was used inside a standard CO_2_ incubator while connected to an external speed regulator and power supply. Because free, non-adherent cells would rapidly undergo anoikis-induced apoptosis [38], osteoblasts grown in AGD medium were trypsinised, counted, and aliquots of 10^6^ cells were pre-attached to sterile MicroHex microcarriers (Nunc) using 1 ml of 50 cm^2^/ml MicroHex per 10^6^ cells, and incubated 4 days in AGD medium at 30°C in culture plates coated with sterile 4% agarose (Gibco) to prevent attachment [39]. The medium was changed every second day. Primary osteoblasts attach to the surface of the microcarriers and develop in a manner similar to osteoblasts plated on standard culture plasticware except for the fact that the cells create three-dimensional connections with more than one plastic surface, resulting in microcarrier-cell aggregates (Fig 2B and 2C). Within 24 hours of attachment at 1*g*, the cell/microcarrier structures assembled into clumps visible to the naked eye which mimicked the more natural cellular environment found in a living organism. After 5 days, MicroHex-attached osteoblasts (5 x 10^6^) were transferred to 10-ml sterile Synthecon HARV chambers containing AGD medium which were placed inside a standard cell incubator (37°C, 5% CO_2_). The units were rotated at optimized speed (20 rpm) to maintain the constant free fall state characteristic of orbital flight and which is the cause of weightlessness. The rotation simulated 0*g* while avoiding shear forces due to excessive rotation and collision with vessel walls. Two chambers were rotated simultaneously on the same rotation axis, one containing the untreated control receiving daily medium change, the other receiving a daily 2-h PTHrP_1-36_ 10^-8^M treatment followed by fresh medium. Cells on microcarriers but growing in AGD medium in agarose-coated plasticware in a standard cell culture incubator were used as 1g controls and received the same regimen of treatments or medium changes. Triplicate experiments were conducted with conditions identical to those aboard the satellite. At the end of the 6^th^ day, cells were collected, trypsinized to release them from the microcarriers, and aliquots analysed for viability by Trypan Blue analysis. Treatment and media change conditions for 6-week experiments were identical to those lasting 6 days.

### Trypan Blue viability test

During long-term experiments, sampling from the HARV rotating chamber was conducted at weekly intervals. Aliquots of cell-microcarriers aggregate were quickly collected in sterile fashion from the momentarily-immobilised HARV, and transferred to sterile microfuge tubes. The aggregates were washed with growth medium then trypsinized in tubes kept horizontally for 20 minutes at 37°C. After cell detachment, the tubes were put vertically in racks to sediment the microcarriers, and the supernatant collected. Microcarriers were rinsed twice with growth medium to collect residual cells, the trypsinised combined volumes were centrifuged, the cells resuspended in growth medium and stained with Trypan Blue for viability [40].

### RNA extraction and microarrays

Collected cells from duplicate experiments were lysed on MicroHex microcarriers using Trizol (InVitrogen, Carlsbad, CA) according to manufacturer’s intructions, frozen at -80°C and sent for RNA extraction and microarray analysis to the Centre d’Innovation de Génome Québec, (Montréal, QC). Illumina Mouse Ref-8 v2.0 slides (25,697 probes) were hybridized with trabecular osteoblast samples (*Pthrp* ^+/+^ and *Pthrp* ^-/-^) from experiments in 1*g* or in simulated microgravity for 6 days, conducted with or without intermittent (2-h daily) PTHrP _1–36_ treatment (8 different conditions in total). Data pre-processing and analysis of expressions levels results were conducted using the FlexArray software version 1.5 from Génome Québec [41] http://genomequebec.mcgill.ca/FlexArray. Normalization was performed with the Lumi package and the data were analyzed using EB Wright and Simon statistical analysis. We retained only gene lists with probe values of p ≤ 0.05 for analysis. Cluster analysis was performed with the Database for Annotation, Visualization and Integrated Discovery online functional annotation tool [42] and by manual searches. Venn diagrams were made using FunRich analysis tool [43]. Microarray data was deposited in the NCBI GEO database and is available under accession number GSE 78980.

### Analysis software, statistics

Statistical analyses of viability experiments were conducted using GraphPad Prism 4.0 for Windows (GraphPad Software, San Diego, CA), using one-way ANOVA with Bonferroni’s multiple comparison post-test. Results were considered significant at p ≤ 0.05.

## Results

### Endogenous PTHrP and exogenous PTHrP_1-36_ protect trabecular osteoblasts from Space microgravity-induced apoptosis

In order to investigate the role of PTHrP in bone cells exposed to space microgravity, trabecular and calvarial osteoblasts obtained from bones of *Pthrp* ^-/-^ fetal mice and *Pthrp* ^+/+^ littermates [26] were attached to CC2 culture slides and inserted into sterile bioreactors connected online to the automated experimental trays (Fig 1A). The 6-day experiment involved a daily 2-hour treatment with 10^-8^ M PTHrP _1–36_ peptide, or a control with growth medium change only for both the 0*g* and 1*g* trays (S1 Table). After return to Earth, an analysis of apoptotic cells was conducted by the ssDNA method [37]. A comparison of *Pthrp* ^+/+^ TOs in the 1*g* tray with equivalent cells aboard the Foton3 satellite indicates that a 6-day exposure to microgravity causes no significant induction of apoptosis. For *Pthrp* ^*-/-*^ TOs, however, a 20.4 ± 5.0% increase in apoptotic cells was observed at 0*g* over the 6-day period compared to the equivalent *Pthrp* ^-/-^ cells which had remained on Earth (Fig 1B white bars). Intermittent treatment with 10^-8^M PTHrP _1–36_ peptide reduced the number of apoptotic cells in 0g compared to 1g by 11.6 ± 4.1% and 50.2% ± 5.4% for ^+/+^ and ^-/-^ TOs respectively (Fig 1B black bars) (p < 0.01). In contrast, over the 6-day period, osteoblasts of calvarial origin (COs) showed little difference in apoptosis due to microgravity exposure although *Pthrp* ^*-/-*^ COs displayed elevated apotosis proportions compared to ^+/+^ COs regardless of gravity conditions (Fig 1C). COs were not treated with PTHrP due to the limited reaction volumes available within the experimental trays. These data implicate PTHrP in TO survival in short-term microgravity exposure, and show that in the absence of endogenous PTHrP, intermittent treatment with exogenous PTHrP _1–36_ reduces the number of cells undergoing apoptosis.

### Allelic effect of endogenous PTHrP levels in simulated microgravity, and compensation by exogenous PTHrP _1–36_

In order to reproduce and expand Space flight results on the role of endogenous and exogenous PTHrP in trabecular octeoblasts, we used the Revolving Cell Culture System Earth-based microgravity simulator from Synthecon (Fig 2A). TOs from *Pthrp* ^+/+^, *Pthrp* ^+/-^ and *Pthrp* ^-/-^ fetal animals attached to sterile MicroHex microcarriers (Fig 2B) spontaneously transformed into three-dimensional structures within 24 h (Fig 2C) and these aggregates remained intact throughout the experiment. The low negative impact of our design is evident in the excellent survival of various types of osteoblasts in the Synthecon chambers for extended periods of time (see next section).

Cell-microcarrier aggregates were collected after 6 days, trypsinised and analysed by Trypan blue for viability. After 6 days, *Pthrp*^+/+^ TOs presented a low (7.5±1.1%) percentage of non-viable cells in both 0*g* and 1*g* experimental set-ups. Heterozygous *Pthrp* ^+/-^ TOs displayed a higher percentage of non-viable cells (22% ± 2.1 at 0*g* compared to 13.5±1.9% at 1*g*) p < 0.01, and *Pthrp* ^-/-^ TOs were greatly affected by weightlessness (non-viable cells: 43±3.1% at 0*g* compared to 20 ± 2.5% at 1*g*) (Fig 2D white bars) p < 0.001. Intermittent treatment with PTHrP _1–36_ made little difference at 1*g* or 0*g* for *Pthrp*^+/+^ TOs but reduced the percentage of non-viable cells from 22.1 to 12.7 ± 4% at 0*g* in the heterozygous TOs. In *Pthrp* ^-/-^ cells, PTHrP _1–36_ intermittent treatment decreased non-viable cells from 20±3.2% to 11±2.5% at 1*g*, but there was a striking reduction (43±4.2% to 11±5.0%) in numbers of non-viable cells at 0*g* (Fig 2D black bars) p < 0.001. These data confirm the results obtained in Space: there is little to no effect of microgravity on *Pthrp*^*+/+*^ cell viability in 0g over 6 days, but incremental loss of viability as *Pthrp* levels of expression decrease. Intermittent PTHrP_1-36_ maintains or increases viability, especially in *Pthrp*^*-/-*^ TOs. The simulated microgravity experiments demonstrate again the greater vulnerability of *Pthrp*-ablated trabecular osteoblasts to microgravity and point to a definite allelic effect for *Pthrp* ablation.

### Long-term exposure to simulated microgravity is detrimental to wild-type trabecular osteoblasts but compensated for by exogenous PTHrP_1-36_ treatment

**Fig 3:**
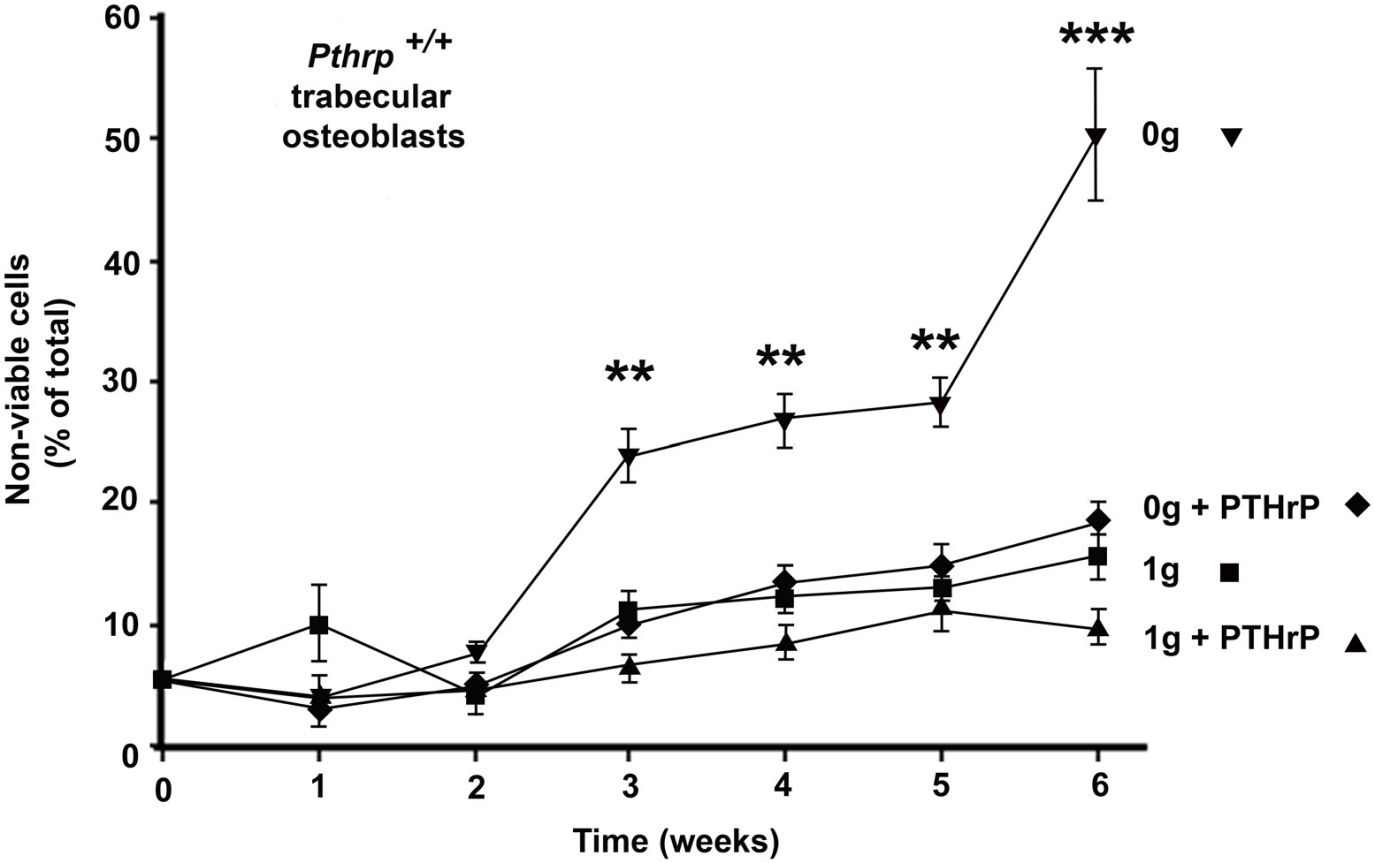
Long-term exposure to simulated microgravity is detrimental to wild-type trabecular osteoblasts but compensated for by exogenous intermittent PTHrP_1-36_ treatment. *Pthrp*^*+/+*^ trabecular osteoblasts at 1*g* (■) or 0*g* (▼). *Pthrp*^*+/+*^ trabecular osteoblasts treated with PTHrP_1-36_ 10^-8^M.at 1*g* (▲) or 0*g* (♦). (triplicate 6-week experiments). Viability estimated by Trypan blue. ** p < 0.01, *** p < 0.001.

In order to investigate the effect of longer microgravity exposure on wild-type TOs, 6-week experiments were conducted with wild-type *Pthrp*^+/+^ TO cells attached to microcarriers and exposed to RCCS-simulated microgravity (0*g*) or to normal gravity (1*g*). Incubation conditions, media changes and intermittent daily PTHrP _1–36_ treatments (10^-8^ M) were identical to those described above. Samples of osteoblast aggregates on microcarriers were drawn from 1*g* and 0*g* experimental set at weekly intervals, trypsinized, and cell viability assessed by Trypan blue staining. The three-dimensional cell aggregates on micro-carriers provide *in vitro* growth conditions that are closer to those found *in vivo* compared to single layer cultures [44] and allow excellent survival of wild-type trabecular osteoblasts in normal gravity over the course of 6 weeks; wild-type *Pthrp*^+/+^ TOs attached to MicroHex carriers easily survived 6 weeks in culture plates in normal 1*g* gravity, with only a 10–15% overall loss in viability (Fig 3). In contrast, *Pthrp*^+/+^ TOs on MicroHex carriers exposed to simulated microgravity (0*g*) started to lose viability after 2 weeks and presented a striking 50% non-viable cells after 6 weeks. Importantly, a 2-h daily treatment with PTHrP_1-36_ over the 6-week period reduced this loss of viability to 15%, similar to that of cells left at 1*g*. As expected from results observed in Fig 2, *Pthrp*^*-/-*^ TOs did not survive past week 2 (not shown). These results suggest that microgravity-induced molecular events leading to cell death occur early in wild-type TOs and that intermittent treatment with PTHrP _1–36_ peptide maintains viability in these cells over the course of 6 weeks.

### Long-term exposure to simulated microgravity has little negative effect on calvarial osteoblasts

**Fig 4:**
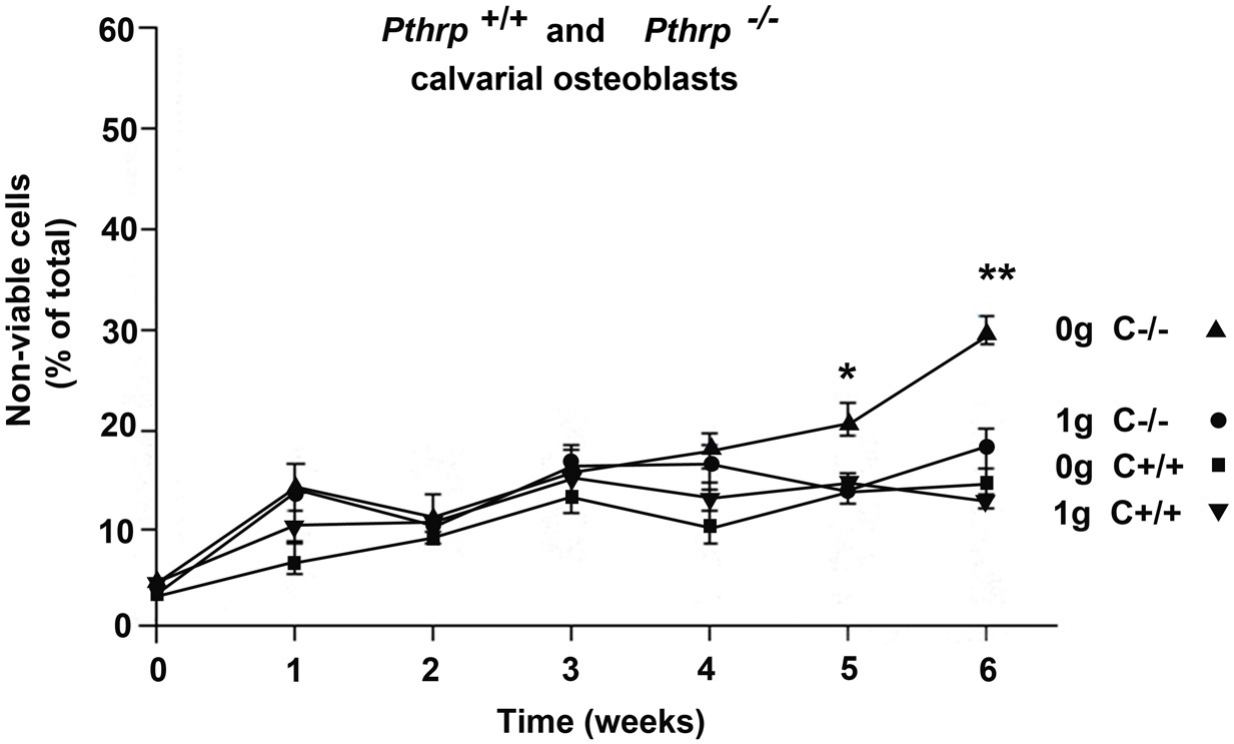
Long-term exposure to simulated microgravity has little negative effect on calvarial osteoblasts. *Phtrp*^*+/+*^ calvarial osteoblasts at 1g (▼) or 0g (■). *Pthrp*^*-/-*^ calvarial osteoblasts at 1g (●) or 0g (▲). (triplicate 6-week experiments). No exogenous PTHrP_1-36_ treatment was applied in this experiment. Viability estimated by Trypan blue. * p < 0.05, ** p < 0.01.

Long-term experiments (6-weeks) were conducted in the RCCS equipment with calvarial osteoblasts from *Pthrp* ^+/+^ and ^-/-^ mice. CO cells attached to MicroHex carriers produced three-dimensional structures similar to those observed in trabecular experiments. Experimental 0*g* and 1*g* conditions were identical to above except that no exogenous PTHrP _1–36_ treatment was conducted. Fig 4 shows that COs from wild type *Pthrp* ^+/+^ mice maintain comparable viability in 0*g* and 1*g* for at least 6 weeks (10 to 12% non-viable cells). As suggested by results from Fig 1C, *Pthrp* ^-/-^ COs are more sensitive to 0g but the percentage of non-viable cells after 6 weeks at 0*g* is only 13% above that of *Pthrp* ^-/-^ calvarial cells at 1*g*. These results indicate that wild-type COs are little affected by a 6-week exposure to microgravity, but that PTHrP nevertheless plays some role in resistance to weightlessness in cranium-derived osteoblasts since *Pthrp* ^-/-^ calvarial cells slowly lose viability in 0*g*.

### Microarray analysis conducted on trabecular osteoblasts: effect of 6-day exposure to microgravity, effect of *Pthrp* ablation, and reversal by intermittent PTHrP _1–36_ treatment

Microgravity-induced adverse effects appear early on in TOs exposed to weightless conditions. Since these early events are crucial to all subsequent physiological changes, expression microarrays after 6 days at 0*g* were analysed in order to identify the genes involved. Microarray analysis (Illumina Mouse Ref8) was conducted on *Pthrp*^+/+^ wild type TOs exposed to 6 days of simulated microgravity to check for early gene expression changes that lead to microgravity-induced alterations detectable in the following weeks. 0*g*-induced changes in *Pthrp*^*+/+*^ TOs were also compared with those resulting from *Pthrp* gene ablation. Reversal was examined upon intermittent treatment with the PTHrP_1-36_ peptide (10^-8^M) of *Pthrp*-ablated and 0*g*-exposed TO cells.

In *Pthrp*^+/+^ trabecular osteoblasts, a 6-day period in simulated microgravity significantly upregulated 59 genes (fold change > 2.0, *p* < 0.05, complete gene list in S2A Table), and downregulated 129 genes (fold change < 0.5, *p* < 0.05, complete gene list in S2B Table) compared to *Pthrp*^+/+^ TOs which remained at 1*g* (total number of genes significantly affected by 6 days at 0g = 188). *Pthrp* gene ablation (i.e. comparison of *Pthrp*^+/+^ and *Pthrp*^-/-^ TOs at 1*g*) significantly upregulated 270 genes (fold change > 2.0, *p* < 0.05, complete genelist in S3A Table) and downregulated 493 genes (fold change < 0.5, *p* < 0.05, complete gene list in S3B Table)(total number of genes significantly affected by *Pthrp* ablation = 763).

**Fig 5:**
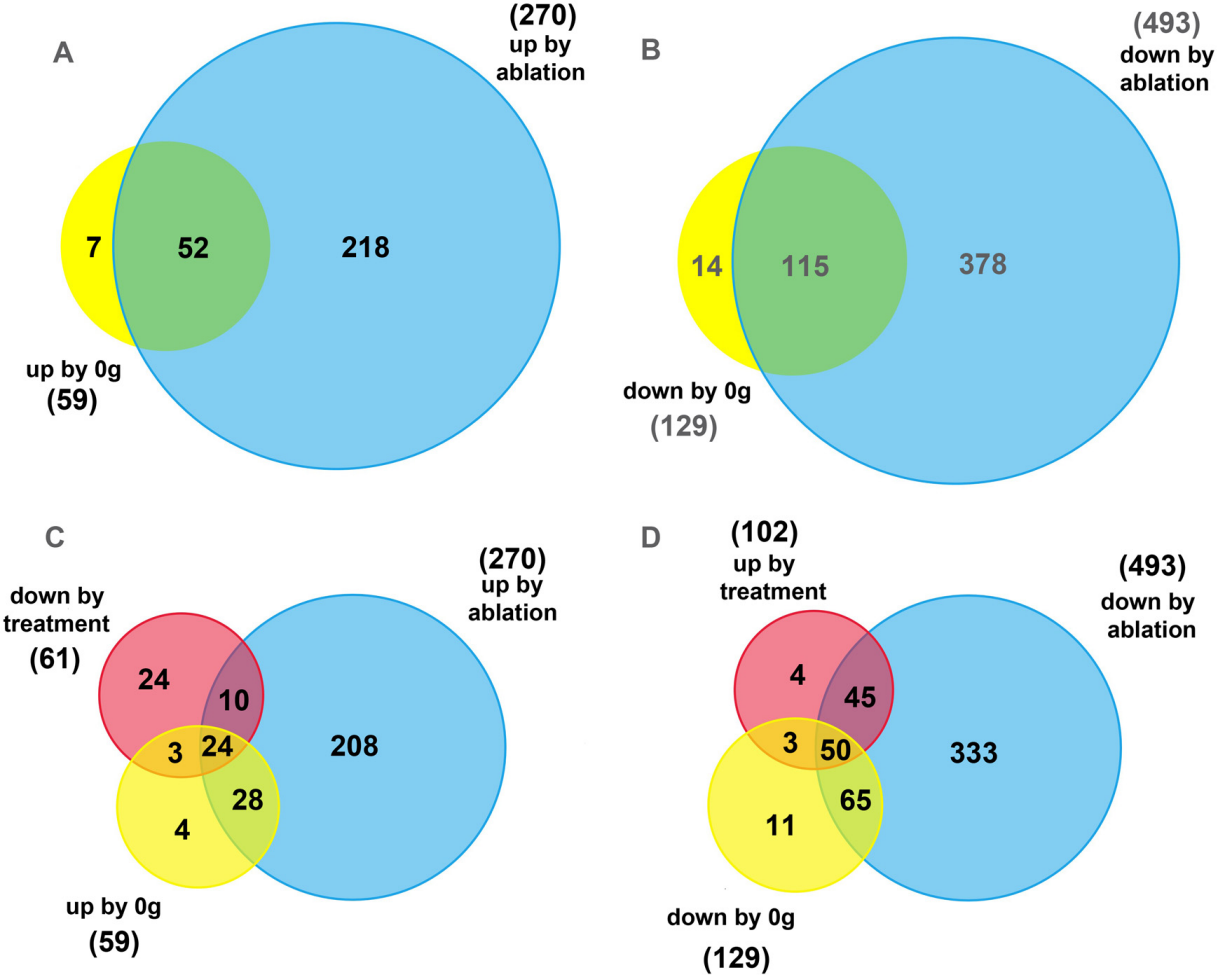
Venn diagrams illustrating the number of trabecular osteoblast genes significantly up- or downregulated by microgravity exposure (6 days) or by *Pthrp* ablation, and reversed by PTHrP_1-36_ treatment. **(A)** 52 genes are upregulated by both 0g (yellow) and *Pthrp* ablation (blue). Fold change > 2.0, *p* < 0.05. **(B)** 115 genes are downregulated by both 0*g* (yellow) and *Pthrp* ablation (blue). Fold change < 0.5, *p* < 0.05. **(C)** 24 genes are upregulated by both 0g (yellow) and *Pthrp* ablation (blue), and are also downregulated (central intersect) by PTHrP_1-36_ treatment (red). Fold change < 0.65, *p* < 0.05. **(D)** 50 genes are downregulated by both 0*g* (yellow) and *Pthrp* ablation (blue), and upregulated (central intersect) by PTHrP_1-36_ treatment (red). Fold change >1.5, *p* < 0.05.

With this information, a parallel was established between the effects of microgravity exposure on wild-type *Pthrp*^+/+^ TOs, and the changes in gene expression occurring after *Pthrp* ablation at 1*g*. 52 upregulated and 115 downregulated genes were found to be common between 0*g* exposure and *Pthrp* ablation effects (Fig 5A and 5B, intersects, complete list of common genes in Tables 1 and 2). In all, 88% of the genes modified by microgravity were similarly affected by *Pthrp* ablation. Clustering analysis suggests the common genes are mainly related to the following metabolic categories: prolactins (up: *Plf2*, *Mrpplf3)*, genes involved in bone growth, mineralization and bone morphogenic protein (BMP) metabolism (up: *Aqp5; Grem1*, *Inhba*, *Nbl1*, *Cryab*, *Ank*, *CD44*, *Scx*, *Mustn1*, *Tnfrsf11b* (osteoprotegerin), *Bdnf*, *Stmn2*; down: *Ptn* (pleiotrophin), *Hp*, *Scara5*, *Itm2a*, *Ptn*, *Dlk1*, *Ramp2*, *Dab*, *Eno1*, *CD14*, *Hemp1*, *Lgmn*, *Txnip*, *Ptx3)*, apoptosis/survival (down: *Clu*, *Rbm3*), extracellular matrix components (up: *Timp3*, *Prelp*, *Ctgf*, *Col7a1*; down: *Lum*, *Dcn*, *H19*, *Nid2*, *Col3a1*, *Dpt*, *Ctsc*), Wnt signaling (up: *Gpc1*, *Nkd2*; down: *Sfrp 2* and *1*), IGF signaling (down: *Igfbp5* and *3*, *Igf2*), heat-shock proteins (up: *Hspb1* and *8*), chemokines (down: *Cxcl12* and *4*, *Ccr5*), cell cycle control (up: *Ccng1*, down: *Cdkn1c*), as well as a variety of histone genes. Importantly, none of the significantly-affected genes presented a contradictory direction change in expression level between *Pthrp* ablation or microgravity treatment.

**Table 1:** Genes (52) upregulated by both microgravity and *Pthrp* ablation.

Target ID	Definition (all probes: *Mus musculus*)	RefSeq ID	Fold change 0g	Fold change ablation	C
PLF2	prolactin family 2, subfamily c, member 3 (Prl2c3), mRNA.	NM_011118.1	12.81098	20.67081	P
MRPPLF3	prolactin family 2, subfamily c, member 4 (Prl2c4), mRNA.	NM_011954.2	11.76853	19.04649	P
AQP5	aquaporin 5 (Aqp5), mRNA.PREDICTED: similar to aquaporin 5 (LOC100046616), mRNA.	NM_009701.4XM_001476512.1	7.7909763.627969	25.586929.547247	A
GREM1	gremlin1 (Grem1) mRNA.	NM_011824.1	4.151464	5.17375	B
GPC1	glypican 1 (Gpc1), mRNA.	NM_016696.3	4.13393	4.008327	W
ALDH3A1	aldehyde dehydrogenase family 3, subfamily A1 (Aldh3a1), mRNA.	NM_007436.1	3.821048	2.733907	
ASS1	argininosuccinate synthetase 1 (Ass1), mRNA.	NM_007494.3	3.663361	2.781254	
NKD2	naked cuticle 2 homolog (Drosophila) (Nkd2), mRNA.	NM_028186.3	3.63063	9.964373	W
NQO1	NAD(P)H dehydrogenase, quinone 1 (Nqo1), mRNA.	NM_008706.4	3.439677	9.410905	
INHBA	inhibin beta-A (Inhba), mRNA.	NM_008380.1	3.374437	6.03861	B
HIST1H4H	histone cluster 1, H4h (Hist1h4h), mRNA.	NM_153173.2	3.122563	6.525515	H
HSPB1	heat shock protein 1 (Hspb1), mRNA.	NM_013560.1	2.937195	3.686913	HS
CRIP2	cysteine rich protein 2 (Crip2), mRNA.	NM_024223.1	2.87678	4.260978	
TIMP3	tissue inhibitor of metalloproteinase 3 (Timp3), mRNA	NM_011595.2NM_011595.2	2.8337662.168426	4.7180244.573879	E
ESD	esterase D/formylglutathione hydrolase (Esd), mRNA.	NM_016903.2	2.617979	4.782361	
PRELP	proline arginine-rich end leucine-rich repeat (Prelp), mRNA.	NM_054077.3	2.563206	2.682429	E
1110032E23RIK	RIKEN cDNA 1110032E23 gene (1110032E23Rik), mRNA	NM_133187.2	2.532211	5.501027	
GDF15	growth differentiation factor 15 (Gdf15), mRNA.	NM_011819.1	2.506674	2.841855	T
AHRR	aryl-hydrocarbon receptor repressor (Ahrr), mRNA.	NM_009644.2	2.366026	2.51242	
NBL1	neuroblastoma, suppression of tumorigenicity 1 (Nbl1), mRNA.	NM_008675.1	2.362421	2.214473	B
CRYAB	crystallin, alpha B (Cryab), mRNA.	NM_009964.1	2.332638	5.135748	B
FABP3	fatty acid binding protein 3, muscle and heart (Fabp3), mRNA.	NM_010174.1	2.32906	7.70136	
MDM2	transformed mouse 3T3 cell double minute 2 (Mdm2), mRNA.	NM_010786.2NM_010786.3	2.318342.246531	2.8090142.71183	
KCTD10	potassium channel tetramerisation domain containing 10 (Kctd10), mRNA.	NM_026145.3	2.279135	2.72172	IC
SGK	serum/glucocorticoid regulated kinase 1 (Sgk1), mRNA.	NM_011361.1	2.240079	2.362139	
ANK	progressive ankylosis (Ank), mRNA.	NM_020332.3	2.228785	9.115022	B
EPHX1	epoxide hydrolase 1, microsomal (Ephx1), mRNA.	NM_010145.2	2.215451	2.907278	
WHRN	whirlin (Whrn), transcript variant 3, mRNA.	NM_001008792.1	2.202689	2.327795	
CD44	CD44 antigen (Cd44), transcript variant 2, mRNA.	NM_001039150.1	2.195092	4.927895	B
CTGF	connective tissue growth factor (Ctgf), mRNA.	NM_010217.1	2.193812	3.29075	E
SCX	scleraxis (Scx), mRNA.	NM_198885.2	2.185468	4.251521	B
MUSTN1	musculoskeletal, embryonic nuclear protein 1 (Mustn1), mRNA.	NM_181390.1NM_181390.2	2.1853642.109783	3.9570883.099647	B
HIST1H2BJ	histone cluster 1, H2bj (Hist1h2bj), mRNA.	NM_178198.1	2.173656	2.755197	H
EEF1A2	eukaryotic translation elongation factor 1 alpha 2 (Eef1a2), mRNA.	NM_007906.2	2.153269	2.293173	A
TNFRSF11B	tumor necrosis factor receptor superfamily, member 11b (osteoprotegerin) (Tnfrsf11b), mRNA.	NM_008764.3	2.129809	9.058214	B
HOXC6	homeo box C6 (Hoxc6), mRNA.	NM_010465.2	2.107019	2.215212	
2300002D11RIK	RIKEN cDNA 2300002D11 gene (2300002D11Rik), mRNA.	NM_001081156.1	2.097322	4.2774	
BDNF	brain derived neurotrophic factor (Bdnf), transcript variant 3, mRNA.	NM_001048141.1	2.091327	7.461118	B
HSPB8	heat shock protein 8 (Hspb8), mRNA.	NM_030704.1	2.090601	2.42948	H
PDGFA	platelet-derived growth factor, alpha chain (Pdgfa) mRNA.		2.084386	2.631637	PD
CCNG1	cyclin G1 (Ccng1), mRNA.	NM_009831.2	2.083259	3.109058	CC
HIST1H2BM	histone cluster 1, H2bm (Hist1h2bm), mRNA.	NM_178200.1	2.072647	2.824912	H
HIST1H2BH	histone cluster 1, H2bh (Hist1h2bh), mRNA.	NM_178197.1	2.069317	2.693571	H
HIST1H2BF	histone cluster 1, H2bf (Hist1h2bf), mRNA.	NM_178195.1	2.066524	2.55937	H
COL7A1	collagen, type VII, alpha 1 (Col7a1), mRNA.	NM_007738.3	2.044317	3.129226	E
A630005A06RIK	TBC1 domain family, member 2 (Tbc1d2), mRNA.	NM_198664.3	2.044002	3.518042	
GSTP1	glutathione S-transferase, pi 1 (Gstp1), mRNA.	NM_013541.1	2.039079	2.672256	
TNFRSF12A	tumor necrosis factor receptor superfamily, member 12a (Tnfrsf12a), mRNA.	NM_013749.1	2.026671	3.449432	
SNRPN	SNRPN upstream reading frame (Snurf), mRNA.	NM_033174.2	2.014161	3.736806	
HIST1H2BK	histone cluster 1, H2bk (Hist1h2bk), mRNA.	NM_175665.1	2.012974	2.669902	H
STMN2	stathmin-like 2 (Stmn2), mRNA.	NM_025285.2	2.007183	3.763786	B
DOS	downstream of Stk11 (Dos), mRNA.	NM_015761.2	2.00261	3.474366	

Genes (52) upregulated by both microgravity and *Pthrp* ablation. All fold changes > 2. All p values < 0.05. Multiple entries for a gene indicate multiple probes. C: Clustering: A: apoptosis/survival, B: bone metabolism and bone morphogenic proteins, CC: cell cycle, E: extracellular matrix, H: histone, HS: heat shock, IC: ion channel, P: prolactins, PD: Platelet-derived growth factor, T: tumor growth factor, W: Wnt.

**Table 2:** Genes (115) downregulated by both microgravity and *Pthrp* ablation.

Target ID	Definition (all probes: *Mus musculus*)	RefSeq ID	Fold change 0g	Fold change ablation	C
SFRP2	secreted frizzled-related protein 2 (Sfrp2), mRNA.	NM_009144.1	0.1316429	0.096523	W
PTN	pleiotrophin (Ptn), mRNA.	NM_008973.2	0.1557338	0.047141	B
IGFBP5	insulin-like growth factor binding protein 5 (Igfbp5), mRNA.	NM_010518.2	0.1603545	0.2130594	I
1500015O10RIK	RIKEN cDNA 1500015O10 gene (1500015O10Rik), mRNA.	NM_024283.2	0.1804316	0.0975587	
IGF2	insulin-like growth factor 2 (Igf2), mRNA.	NM_010514.2	0.2085582	0.026108	I
CLU	PREDICTED: similar to clusterin (LOC100046120), mRNA.	XM_001475611.1	0.213665	0.47421	A
CXCL12	chemokine (C-X-C motif) ligand 12 (Cxcl12), transcript variant 3, mRNA.	NM_013655.2NM_021704.2NM_001012477.1	0.21502010.22348560.236011	0.28323790.40775260.3778157	CH
HP	haptoglobin (Hp), mRNA.	NM_017370.1NM_017370.1	0.22221020.4034571	0.18333460.4167181	B
C3	complement 3 (C3) mRNA.	NM_009778.1	0.2325967	0.1254136	
SCARA5	scavenger receptor class A, member 5 (putative) (Scara5), mRNA.	NM_028903.1	0.2351416	0.1405516	B
LUM	lumican (Lum), mRNA.	NM_008524.1	0.235224	0.0573447	E
MRC1	mannose receptor, C type 1 (Mrc1), mRNA.	NM_008625.1	0.253038	0.0815048	
4930583H14RIK	RIKEN cDNA 4930583H14 gene (4930583H14Rik), mRNA.	NM_026358.2	0.2575598	0.1684547	
2310061N23RIK	interferon, alpha-inducible protein 27 (Ifi27), mRNA.	NM_029803.1	0.2600992	0.0590283	
DCN	decorin (Dcn), mRNA.	NM_007833.4NM_007833.4	0.28897940.3837174	0.06403410.0548521	E
H19	H19 fetal liver mRNA (H19), non-coding RNA.	NR_001592.1	0.2906019	0.0360464	E
PPP1R3C	protein phosphatase 1, regulatory (inhibitor) subunit 3C (Ppp1r3c), mRNA.	NM_016854.2	0.2954025	0.1777717	
RBM3	PREDICTED: similar to RNA binding motif protein 3 (LOC100043257), mRNA.	XM_001480197.1	0.2991486	0.2378549	A
APOD	PREDICTED: similar to apolipoprotein D (LOC100047583), mRNA.	XM_001479138.1	0.2996465	0.1742532	A
ITM2A	integral membrane protein 2A (Itm2a), mRNA.	NM_008409.2	0.2997355	0.138948	B
D930038M13RIK	ABI gene family, member 3 (NESH) binding protein (Abi3bp), transcript variant 1, mRNA.	NM_178790.3	0.3036303	0.2955018	B
PFKL	phosphofructokinase, liver, B-type (Pfkl), mRNA.	NM_008826.3	0.3054363	0.2514627	
OLFML1	olfactomedin-like 1 (Olfml1), mRNA.	NM_172907.2	0.3123395	0.3050197	
HIST1H2AD	histone cluster 1, H2ad (Hist1h2ad), mRNA.	NM_178188.3	0.3149356	0.0061305	
DLK1	delta-like 1 homolog (Drosophila) (Dlk1), mRNA.	NM_010052.4	0.3273854	0.1513731	B
HIST1H2AK	histone cluster 1, H2ak (Hist1h2ak), mRNA.	NM_178183.1	0.3372404	0.0596174	H
2310056P07RIK	family with sequence similarity 162, member A (Fam162a), mRNA.	NM_027342.1	0.3391683	0.2567712	
HIST1H2AF	histone cluster 1, H2af (Hist1h2af), mRNA.	NM_175661.1	0.3396376	0.067387	H
SLC1A3	solute carrier family 1 (glial high affinity glutamate transporter), member 3 (Slc1a3), mRNA.	NM_148938.2	0.3399492	0.1181837	
PSCDBP	cytohesin 1 interacting protein (Cytip), mRNA.	NM_139200.4	0.3403133	0.1328767	
NID2	nidogen 2 (Nid2), mRNA.	NM_008695.2	0.3477556	0.3321977	E
TGFBI	transforming growth factor, beta induced (Tgfbi), mRNA.	NM_009369.1	0.3514812	0.0792747	T
HIST1H2AH	histone cluster 1, H2ah (Hist1h2ah), mRNA.	NM_175659.1	0.352093	0.0777217	
FMO1	flavin containing monooxygenase 1 (Fmo1), mRNA.	NM_010231.2	0.3568025	0.3366457	
EGLN3	EGL nine homolog 3 (C. elegans) (Egln3), mRNA.	NM_028133.1	0.3569504	0.3106277	
DPEP2	dipeptidase 2 (Dpep2), mRNA.	NM_176913.3	0.35774720.3965119	0.14003250.18665	
COL3A1	collagen, type III, alpha 1 (Col3a1), mRNA.	NM_009930.1	0.3599768	0.2207009	E
1200009O22RIK	RIKEN cDNA 1200009O22 gene (1200009O22Rik), mRNA.	NM_025817.3	0.3616481	0.3116025	
HIST1H2AO	histone cluster 1, H2ao (Hist1h2ao), mRNA.	NM_178185.1	0.3618695	0.057145	H
AGTR1A	angiotensin II receptor, type 1a (Agtr1a), mRNA.	NM_177322.2	0.3640873	0.3477361	
LDB2	LIM domain-binding protein 2 (Ldb2) mRNA.	NM_010698.2	0.3694307	0.2963222	
DPT	dermatopontin (Dpt), mRNA.	NM_019759.2	0.369822	0.2973928	E
6330406I15RIK	RIKEN cDNA 6330406I15 gene (6330406I15Rik), mRNA.	NM_027519.1NM_027519.3	0.37182430.3991528	0.19828060.2041744	
RASSF4	Ras association (RalGDS/AF-6) domain family member 4 (Rassf4), mRNA.	NM_178045.3NM_178045.3	0.3723380.3845508	0.36712360.1214973	
PDGFRA	platelet derived growth factor receptor, alpha polypeptide (Pdgfra), transcript variant 1, mRNA.	NM_011058.2	0.3755132	0.394926	PD
HIST1H2AN	histone cluster 1, H2an (Hist1h2an), mRNA.	NM_178184.1	0.3776532	0.0945669	H
ZCCH5	Zinc finger, CCHC domain-containing 5 (Zcchc5), mRNA	NM_199468.2	0.3839341	0.4813192	
IGFBP3	insulin-like growth factor binding protein 3 (Igfbp3), mRNA.	NM_008343.2	0.3878084	0.3767934	I
AOC3	amine oxidase, copper containing 3 (Aoc3), mRNA.	NM_009675.1	0.390921	0.102008	
KNG1	kininogen 1 (Kng1), mRNA.	NM_023125.2	0.3914529	0.2682237	
SERPING1	serine (or cysteine) peptidase inhibitor, clade G, member 1 (Serping1), mRNA.	NM_009776.1	0.3939361	0.2493484	
SFRP1	secreted frizzled-related protein 1 (Sfrp1), mRNA.	NM_013834.1	0.3966504	0.1332278	W
EMR1	EGF-like module containing, mucin-like, hormone receptor-like sequence 1 (Emr1), mRNA.	NM_010130.3	0.4002873	0.0452258	
SIRPB1	signal-regulatory protein beta 1 (Sirpb1), transcript variant 3, mRNA.	NM_001002898.1	0.4006563	0.250706	
CAR9	carbonic anhydrase 9 (Car9), mRNA.	NM_139305.1	0.401926	0.3807879	
SRPX	sushi-repeat-containing protein (Srpx), mRNA.	NM_016911.4NM_016911.4	0.40437110.4169736	0.12827860.1438187	
LBP	lipopolysaccharide binding protein (Lbp), mRNA.	NM_008489.2	0.4046987	0.3596355	
2310006J04RIK	Ankyrin repeat domain 37 (Ankrd 37), mRNA	NM_001039562.1	0.4075139	0.4461992	
CCR5	chemokine (C-C motif) receptor 5 (Ccr5), mRNA.	NM_009917.2NM_009917.2NM_009917.4	0.40916990.44612370.4461237	0.31747080.32485210.3880605	CH
RAMP2	receptor (calcitonin) activity modifying protein 2 (Ramp2), mRNA.	NM_019444.2	0.4105468	0.2878545	B
LSP1	PREDICTED: predicted gene, ENSMUSG00000043795 (ENSMUSG00000043795), mRNA.	XM_001480835.1	0.4114556	0.1243922	
CDKN1C	cyclin-dependent kinase inhibitor 1C (P57) (Cdkn1c), mRNA.	NM_009876.3	0.4116614	0.347614	CC
CORO1A	coronin, actin binding protein 1A (Coro1a), mRNA.	NM_009898.2NM_009898.2NM_009898.2	0.41277540.44359450.4970813	0.08707340.05964910.1696803	
C1QA	complement component 1, q subcomponent, alpha polypeptide (C1qa), mRNA.	NM_007572.2	0.4128893	0.1927809	
SLC2A1	solute carrier family 2 (facilitated glucose transporter), member 1 (Slc2a1), mRNA.	NM_011400.2	0.4167042	0.2991707	
GPR23	G protein-coupled receptor 23 (Gpr23), mRNA.	NM_175271.2	0.4254305	0.4447173	
PDK1	pyruvate dehydrogenase kinase, isoenzyme 1 (Pdk1), nuclear gene encoding mitochondrial protein, mRNA.	NM_172665.3	0.4255101	0.3667602	
CTSC	cathepsin C (Ctsc), mRNA.	NM_009982.2	0.4265272	0.0758005	E
OASL2	2'-5' oligoadenylate synthetase-like 2 (Oasl2), mRNA.	NM_011854.1	0.4272569	0.1159377	
C1QB	complement component 1, q subcomponent, beta polypeptide (C1qb), mRNA.	NM_009777.2	0.4278048	0.0245339	
DAB2	disabled homolog 2 (Drosophila) (Dab2), transcript variant 2, mRNA.	NM_023118.1NM_001008702.1	0.42782040.4450885	0.17925610.1341114	B
RSPO3	R-spondin 3 homolog (Xenopus laevis) (Rspo3), mRNA.	NM_028351.2	0.4293909	0.2896171	W
MFAP2	microfibrillar-associated protein 2 (Mfap2), mRNA.	NM_008546.2NM_008546.2NM_008546.2	0.42967080.47591550.4945922	0.18086140.25899670.2757687	B
GMFG	glia maturation factor, gamma (Gmfg), transcript variant 1, mRNA.	NM_022024.2NM_022024.2	0.43023620.4870403	0.203090.2355645	
SELENBP1	PREDICTED: hypothetical protein LOC100044204 (LOC100044204), mRNA.	XM_001471696.1	0.433163	0.2613255	
MEST	mesoderm specific transcript (Mest), mRNA.	NM_008590.1NM_008590.1	0.43492520.4556044	0.31959320.3918639	
NDRG1	N-myc downstream regulated gene 1 (Ndrg1), mRNA.	NM_008681.2	0.4352534	0.3870171	
MS4A6D	membrane-spanning 4-domains, subfamily A, member 6D (Ms4a6d), mRNA.	NM_026835.2	0.4373044	0.0358791	
ALOX5AP	arachidonate 5-lipoxygenase activating protein (Alox5ap), mRNA.	NM_009663.1	0.4387523	0.0317102	
HIST1H2AG	histone cluster 1, H2ag (Hist1h2ag), mRNA.	NM_178186.2	0.4395184	0.2645484	H
1300002F13RIK	ERBB receptor feedback inhibitor 1 (Errfi1), mRNA.	NM_133753.1	0.4395548	0.2132049	
EVI2A	ecotropic viral integration site 2a (Evi2a), transcript variant 2, mRNA.	NM_010161.3	0.4402088	0.1280774	
CXCL4	chemokine (C-X-C motif) ligand 4 (Cxcl4), mRNA.	NM_019932.2	0.4419611	0.180652	CH
1190002H23RIK	RIKEN cDNA 1190002H23 gene (1190002H23Rik), mRNA.	NM_025427.2	0.4430251	0.3481645	
ENO1	enolase 1, alpha non-neuron (Eno1), mRNA.	NM_023119.1	0.4461543	0.4872021	B
LYZ	lysozyme 1 (Lyz1), mRNA.	NM_013590.3	0.4473266	0.3851333	
CD52	CD52 antigen (Cd52), mRNA.	NM_013706.1	0.44963	0.0749331	
NDRL	N-myc downstream gene 1 (Ndrl), mRNA.		0.4504366	0.4056498	
CYP7B1	cytochrome P450, family 7, subfamily b, polypeptide 1 (Cyp7b1), mRNA.	NM_007825.3	0.450876	0.1997939	
PIRA4	paired-Ig-like receptor A4 (Pira4), mRNA.	NM_011091.1	0.4527747	0.0263051	
RARRES2	retinoic acid receptor responder (tazarotene induced) 2 (Rarres2), mRNA.	NM_027852.1	0.4537163	0.3671236	
CD14	CD14 antigen (Cd14), mRNA.	NM_009841.3	0.4582975	0.1117494	B
ALDH3B1	aldehyde dehydrogenase 3 family, member B1 (Aldh3b1), mRNA.	NM_026316.2	0.4627661	0.3188771	
KCNAB2	potassium voltage-gated channel, shaker-related subfamily, beta member 2 (Kcnab2), mRNA.	NM_010598.2	0.4659567	0.1179869	
C1QG	complement component 1, q subcomponent, C chain (C1qc), mRNA.	NM_007574.2	0.468035	0.3163972	
GALNT9	UDP-N-acetyl-alpha-D-galactosamine:polypeptide N-acetylgalactosaminyltransferase 9 (Galnt9), mRNA.	NM_198306.1	0.4683551	0.3906375	
ADD3	adducin 3 (gamma) (Add3), mRNA.	NM_013758.2	0.4687887	0.306828	
ZFP36L1	zinc finger protein 36, C3H type-like 1 (Zfp36l1), mRNA.	NM_007564.4	0.4703194	0.2280471	
HEMP1	NCK associated protein 1 like (Nckap1l), mRNA.	NM_153505.4	0.4709411	0.0826084	B
2810046M22RIK		NM_026621.1	0.4778584	0.4976117	
LGMN	legumain (Lgmn), mRNA.	NM_011175.2	0.4770529	0.2286424	B
TXNIP	thioredoxin interacting protein (Txnip), transcript variant 1, mRNA.	NM_001009935.2	0.4827267	0.3327381	B
EHD4	EH-domain containing 4 (Ehd4), mRNA.	NM_133838.4	0.483588	0.3327381	E
EMR1	EGF-like module containing, mucin-like, hormone receptor-like sequence 1 (Emr1), mRNA.	NM_010130.1	0.4839607	0.1299728	
ARHGAP20	Rho GTPase activating protein 20 (Arhgap20), mRNA.	NM_175535.3	0.4859805	0.4102931	
PTX3	pentaxin related gene (Ptx3) mRNA	NM_008987.2	0.4867727	0.1136763	B
NCF4	neutrophil cytosolic factor 4 (Ncf4), mRNA.	NM_008677.1	0.4881307	0.1080301	
PFC	complement factor properdin (Cfp), mRNA.	NM_008823.3	0.488737	0.0720321	
SMARCA1	SWI/SNF related, matrix associated, actin dependent regulator of chromatin, subfamily a, member 1 (Smarca1), mRNA.	NM_053123.3	0.4911543	0.3176144	
FCER1G	Fc receptor IgE high affinity 1 gamma polypeptide (Fcerg) mRNA.	NM_010185.2	0.4915371	0.0163625	
CENPA	centromere protein A (Cenpa), mRNA.	NM_007681.2	0.4943203	0.0808812	
1200002N14RIK	RIKEN cDNA 1200002N14 gene (1200002N14Rik), mRNA.	NM_027878.2	0.4949315	0.207954	
TK1	thymidine kinase 1 (Tk1), mRNA.	NM_009387.1	0.4950169	0.1742371	
ALDOA	aldolase A, fructose-bisphosphate (Aldoa), mRNA.	NM_007438.3	0.4959253	0.4941576	
SLPI	secretory leukocyte peptidase inhibitor (Slpi), mRNA.	NM_011414.2	0.4988716	0.2429238	

Genes (115) downregulated by both microgravity and *Pthrp* ablation. All fold changes < 0.5. All p values < 0.05. Multiple entries for a gene indicate multiple probes. C: Clustering: A: apoptosis/survival, B: bone metabolism and bone morphogenic proteins, CC: cell cycle, CH: chemokines, E: extracellular matrix, H: histone, I: Insulin-like growth factor, PD: Platelet-derived growth factor, T: tumor growth factor, W: Wnt.

Because the PTHrP_1-36_ anabolic agent was observed to compensate for weightlessness and reverse the effects of microgravity exposure in TOs (Figs 1, 2 and 3), microarray analysis was conducted on *Pthrp*^*+/+*^ TOs at 0*g* treated intermittently with 10^-8^M PTHrP_1-36_ for 6 days. The expression levels of 102 genes were upregulated and 61 downregulated (total 163, fold change >1.5 or <0.65, *p* value< 0.05, complete list in S4A and S4B Table). Although the treatment peptide corresponds to only 36 amino acids from the N-terminus of PTHrP and not the whole sequence, we expect reversal of some effects induced by microgravity and/or *Pthrp* ablation. Accordingly, 27 of the 0*g*-upregulated genes (45.7%), and 34 of the ablation-upregulated genes (12.5%) were downregulated by PTHrP_1-36_ treatment. Of those, 24 genes were common to all three conditions (Fig 5C, listed in Table 3). 53 of the 0*g*-downregulated (41%) and 95 of the ablation-downregulated genes (19.2%) were upregulated by PTHrP_1-36_ treatment. Of those, 50 genes were common to all three conditions (Fig 5D, listed in Table 4). Clustering analysis revealed the same functional gene categories as above. None of the significantly-affected genes presented a contradictory direction change in expression level between PTHrP_1-36_ treatment and *Pthrp* ablation or microgravity treatment (S1 Fig).

**Table 3:** Genes (24) upregulated by both *Pthrp* ablation and microgravity and downregulated by PTHrP_1-36_ treatment.

Target ID	Definition (all probes: *Mus musculus*)	RefSeqID	Fold change PTHrP 1–36 treatment	C
AQP5	aquaporin 5 (Aqp5), mRNA.PREDICTED: similar to aquaporin 5 (LOC100046616), mRNA.	NM_009701.4XM_001476512.1	0.37050890.4987479	A
TNFRSF11B	tumor necrosis factor receptor superfamily, member 11b (osteoprotegerin) (Tnfrsf11b), mRNA.	NM_008764.3	0.4687011	B
HSPB1	heat shock protein 1 (Hspb1), mRNA.	NM_013560.1	0.4871401	HS
MRPPLF3	prolactin family 2, subfamily c, member 4 (Prl2c4), mRNA.	NM_011954.2	0.5052176	P
NKD2	naked cuticle 2 homolog (Drosophila) (Nkd2), mRNA.	NM_028186.3	0.5150308	W
PLF2	prolactin family 2, subfamily c, member 3 (Prl2c3), mRNA.	NM_011118.1	0.5191368	P
BDNF	brain derived neurotrophic factor (Bdnf), transcript variant 3, mRNA.	NM_001048141.1	0.5334656	B
TIMP3	tissue inhibitor of metalloproteinase 3 (Timp3), mRNA.	NM_011595.2NM_011595.2	0.53376130.6267908	E
CRYAB	crystallin, alpha B (Cryab), mRNA.	NM_009964.1	0.5387105	B
ASS1	argininosuccinate synthetase 1 (Ass1), mRNA.	NM_007494.3	0.5595178	
ESD	esterase D/formylglutathione hydrolase (Esd), mRNA.	NM_016903.2	0.5693265	
INHBA	inhibin beta-A (Inhba), mRNA.	NM_008380.1	0.5792015	B
CD44	CD44 antigen (Cd44), transcript variant 2, mRNA	NM_001039150.1	0.5834	B
GREM1	gremlin1 (Grem1) mRNA.	NM_011824.1	0.5978743	B
GDF15	growth differentiation factor 15 (Gdf15), mRNA.	NM_011819.1	0.5985932	T
MUSTN1	musculoskeletal, embryonic nuclear protein 1 (Mustn1), mRNA.	NM_181390.1	0.6015734	B
MDM2	transformed mouse 3T3 cell double minute 2 (Mdm2), mRNA.	NM_010786.2	0.6034403	
KCTD10	potassium channel tetramerisation domain containing 10 (Kctd10), mRNA.	NM_026145.3	0.61132	IC
EEF1A2	eukaryotic translation elongation factor 1 alpha 2 (Eef1a2), mRNA.	NM_007906.2	0.6231256	A
WHRN	whirlin (Whrn), transcript variant 3, mRNA	NM_001008792.1	0.6237	
EPHX1	epoxide hydrolase 1, microsomal (Ephx1), mRNA	NM_010145.2	0.6255	
HIST1H4H	histone cluster 1, H4h (Hist1h4h), mRNA	NM_153173.2	0.635	H
STMN2	stathmin-like 2 (Stmn2), mRNA	NM_025285.2	0.645	B
SCX	scleraxis (Scx), mRNA.	NM_198885.2	0.6553575	B

All -fold changes < 0.66, all p values < 0.05. Multiple entries for a gene indicate multiple probes. C: Clustering: A: apoptosis/survival, B: bone metabolism and bone morphogenic proteins, E: extracellular matrix, H: histone, HS: heat shock, IC: ion channel, P: prolactin, T: tumor growth factor, W: Wnt.

**Table 4:** Genes (50) downregulated by both *Pthrp* ablation and microgravity and upregulated by PTHrP_1-36_ treatment.

Target ID	Definition (all probes: *Mus musculus*)	RefSeqID	Fold change PTHrP 1–36 treatment	C
RBM3	PREDICTED: similar to RNA binding motif protein 3 (LOC100043257), mRNA.	XM_001480197.1	3.316729	A
HIST1H2AD	histone cluster 1, H2ad (Hist1h2ad), mRNA.	NM_178188.3	3.070246	H
HIST1H2AH	histone cluster 1, H2ah (Hist1h2ah), mRNA.	NM_175659.1	2.644405	H
HIST1H2AN	histone cluster 1, H2an (Hist1h2an), mRNA.	NM_178184.1	2.629741	H
HIST1H2AK	histone cluster 1, H2ak (Hist1h2ak), mRNA.	NM_178183.1	2.619065	H
PTN	pleiotrophin (Ptn), mRNA.	NM_008973.2	2.610166	B
HIST1H2AF	histone cluster 1, H2af (Hist1h2af), mRNA.	NM_175661.1	2.590586	H
HIST1H2AO	histone cluster 1, H2ao (Hist1h2ao), mRNA.	NM_178185.1	2.363633	H
2310061N23RIK	interferon, alpha-inducible protein 27 (Ifi27), mRNA.	NM_029803.1	2.240007	A
CENPA	centromere protein A (Cenpa), mRNA.	NM_007681.2	2.217246	
HIST1H2AG	histone cluster 1, H2ag (Hist1h2ag), mRNA.	NM_178186.2	2.186001	H
LUM	lumican (Lum), mRNA.	NM_008524.1	2.127828	E
ITM2A	integral membrane protein 2A (Itm2a), mRNA.	NM_008409.2	2.072151	B
IGFBP5	insulin-like growth factor binding protein 5 (Igfbp5), mRNA.	NM_010518.2	2.068563	I
DCN	decorin (Dcn), mRNA.	NM_007833.4	2.0663121.731693	E
1500015O10RIK	RIKEN cDNA 1500015O10 gene (1500015O10Rik), mRNA.	NM_024283.2	1.860714	
MFAP2	microfibrillar-associated protein 2 (Mfap2), mRNA.	NM_008546.2	1.8432941.5754411.501827	B
TK1	thymidine kinase 1 (Tk1), mRNA	NM_009387.1	1.8403	
ALOX5AP	arachidonate 5-lipoxygenase activating protein (Alox5ap), mRNA	NM_009663.1	1.8329	
CDKN1C	cyclin-dependent kinase inhibitor 1C (P57) (Cdkn1c), mRNA.	NM_009876.3	1.797543	CC
GPR23	G protein-coupled receptor 23 (Gpr23), mRNA	NM_175271.2	1.7145	
CD52	CD52 antigen (Cd52), mRNA.	NM_013706.1	1.688404	
C1QA	complement component 1, q subcomponent, alpha polypeptide (C1qa), mRNA.	NM_007572.2	1.685787	
CTSC	cathepsin C (Ctsc), mRNA.	NM_009982.2	1.68393	E
ALDH3B1	aldehyde dehydrogenase 3 family, member B1 (Aldh3b1), mRNA.	NM_026316.2	1.678022	
TGFBI	transforming growth factor, beta induced (Tgfbi), mRNA.	NM_009369.1	1.664148	T
FMO1	flavin containing monooxygenase 1 (Fmo1), mRNA.	NM_010231.2	1.657767	
IGF2	insulin-like growth factor 2 (Igf2), mRNA.	NM_010514.2	1.656335	I
CYP7B1	cytochrome P450, family 7, subfamily b, polypeptide 1 (Cyp7b1), mRNA.	NM_007825.3	1.652679	
CXCL12	chemokine (C-X-C motif) ligand 12 (Cxcl12) mRNA.	NM_013655.2	1.637518	CH
LDB2	LIM domain-binding 2 (Ldb2) mRNA.	NM_010698.2	1.634978	
CORO1A	coronin, actin binding protein 1A (Coro1a), mRNA.	NM_009898.2	1.6294451.620518	
EMR1	EGF-like module containing, mucin-like, hormone receptor-like sequence 1 (Emr1), mRNA.	NM_010130.3	1.626247	
KNG1	kininogen 1 (Kng1), mRNA.	NM_023125.2	1.5999	
OLFML1	olfactomedin-like 1 (Olfml1), mRNA.	NM_172907.2	1.599738	
AOC3	amine oxidase, copper containing 3 (Aoc3), mRNA.	NM_009675.1	1.598282	
GMFG	glia maturation factor, gamma (Gmfg), transcript variant 1, mRNA.	NM_022024.2	1.5965611.532455	
NCF4	neutrophil cytosolic factor 4 (Ncf4), mRNA.	NM_008677.1	1.593552	
SFRP2	secreted frizzled-related protein 2 (Sfrp2), mRNA.	NM_009144.1	1.589225	W
SMARCA1	SWI/SNF related, matrix associated, actin dependent regulator of chromatin, subfamily a, member 1 (Smarca1), mRNA.	NM_053123.3	1.5706	
SIRPB1	signal-regulatory protein beta 1 (Sirpb1), transcript variant 3, mRNA.	NM_001002898.1	1.559451	
RASSF4	Ras association (RalGDS/AF-6) domain family member 4 (Rassf4), mRNA.	NM_178045.3	1.558789	
KCNAB2	potassium voltage-gated channel, shaker-related subfamily, beta member 2 (Kcnab2), mRNA.	NM_010598.2	1.556591	IC
LSP1	PREDICTED: predicted gene, ENSMUSG00000043795 (ENSMUG00000043795), mRNA	XM_001480835.1	1.5468	
COL3A1	collagen, type III, alpha 1 (Col3a1), mRNA.	NM_009930.1	1.531741	E
SRPX	sushi-repeat-containing protein (Srpx), mRNA.	NM_016911.4	1.522525	
DAB2	disabled 2 mitogen-responsive phosphoprotein (Dab2) mRNA	NM_023118.1	1.519832	
EVI2A	ecotropic viral integration site 2a (Evi2a), transcript variant 2, mRNA.	NM_010161.3	1.517936	
NID2	nidogen 2 (Nid2), mRNA.	NM_008695.2	1.506611	E
C1QB	complement component 1, q subcomponent, beta polypeptide (C1qb), mRNA.	NM_009777.2	1.503228	

All -fold changes > 1.5, all p values < 0.05. C: Clustering: A: apoptosis/survival, B: bone metabolism and bone morphogenic proteins, CC: cell cycle, CH: chemokines, E: extracellular matrix, H: histone, I: Insulin-like growth factor, IC: ion channel, T: tumor growth factor, W: Wnt.

## Discussion

Exposure to Space microgravity causes an important and rapid decrease in the BMD of astronauts’ weight-bearing bones similar to that observed in disuse osteoporosis on Earth [45]. Exercise in weightless conditions does not effectively counter spine and hip bone loss, and a better understanding of bone formation/resorption uncoupling mechanisms under these conditions is needed in order to provide effective countermeasures useful to flight personnel as well as to patients suffering from disuse-induced osteoporosis on Earth. Microgravity has been reported to reduce the expression of PTHrP in lung and bone cells, leading to speculation that this molecule can act as a gravisensor [33]. In view of the fact that bone loss appears to result in significant part from osteoblast dysfunction, we investigated the mechanistic implications of PTHrP metabolism in 0*g*-induced skeletal disorders using mouse wild-type trabecular osteoblasts (*Pthrp*^*+/+*^) which mimic astronauts’ TOs at the start of their mission. Comparison of viability of the wild-type cells with *Pthrp*^*+/-*^ and ^-/-^ TOs confirmed the involvement of PTHrP in resistance to microgravity: a 6-day exposure to microgravity (actual and simulated) significantly decreased viability of *Pthrp*^*-/-*^ trabecular osteoblasts while *Pthrp*^*+/+*^ cells appeared unaffected. An allelic dose-effect for PTHrP expression was even observed using heterozygous *Pthrp*^*+/-*^ TOs in simulated 0*g*. Although *Pthrp*^*+/+*^ cells maintained viability after 6 days at 0*g*, a crucial observation made during exposure of TOs to longer periods of simulated microgravity revealed that the astronaut-mimicking *Pthrp*^*+/+*^ TOs start to lose viability at 2 weeks, suggesting early metabolic changes in the wild-type cells at 0*g*. Importantly, this sharp decline in viability could be countered by treatment with 10^-8^M PTHrP_1-36_ peptide which, when administered intermittently (in a pulsatile manner), has anabolic properties, in contrast with a continuous treatment which causes downregulation of osteogenic genes [36]. These observations confirmed the role of PTHrP in cell survival in microgravity in trabecular osteoblasts. In the present study, calvarial osteoblasts appeared much less sensitive to 0*g* conditions than trabecular osteoblasts, an observation which agrees with those made in human 6° head-down bedrest prolonged tests (which effectively mimic weightlessness) where weight-bearing bones incur substantial skeletal loss but cranium bones in fact appear to gain bone mass [46].

The mechanisms by which PTHrP counters the signal of absent gravity were investigated through expression array analyses revealing that radical changes were already occurring in *Pthrp*^*+/+*^ TOs after 6 days in microgravity. Strikingly, the genes affected by 0*g* present an 87% overlap with genes affected by *Pthrp* ablation (comparison of *Pthrp*^*+/+*^ and *Pthrp*^*-/-*^ TOs at 1*g*), and all changes proceed in the same direction for ablation and 0*g* exposure. This observation indicates that microgravity-induced metabolic changes in wild-type trabecular osteoblasts proceed mostly through PTHrP-controlled genes, affirming a prominent role of PTHrP in cell survival under microgravity. Among the most up- or downregulated genes common to 0*g* and *Pthrp* ablation are genes encoding members of the prolactin family, several genes involved in bone growth including Wnt signaling and insulin-like growth factor (IGF) signaling, genes encoding extracellular matrix (ECM)-related proteins and involved in BMP metabolism and, in mineralization, heat-shock proteins, chemokines, and genes encoding apoptosis/survival, cell cycle control as well as histones. A comparison of our microarray results with expression changes in wild-type mouse 2T3 pre-osteoblasts attached to growth container surface and exposed to simulated 0g for 3 days [47] confirms the loss of bone growth, mineralization, bone morphogenic and extra-cellular matrix proteins upon exposure to simulated microgravity by similar changes in expression of numerous common genes at 0g: osteoprotegerin, RAMP 2, lumican, decorin and various collagen proteins. Several cell signaling molecules are also similarly affected by microgravity in both studies: PDGFRα, IGF2, SFRP2, and members of the TNF and Fos family. The Wnt/βcatenin pathway is essential for bone formation in response to mechanical loading [48], and in our study, expression of mechanosensitive genes such as SFRP1 and 2 which regulate the Wnt pathway [49] was very strongly inhibited by exposure to 0g. This implicates the PTHrP pathway in this mechansensitive pathway in microgravity conditions, and agrees with results of Maycas *et al* [50] who report the involvement of PTH1R as a mechanosensor in osteocyte survival and Chen et al [51] who suggest PTHrP as a candidate mediator of the anabolic effects of mechanical force on bone.

Intermittent treatment with PTHrP_1-36_ of TOs not only reversed loss of viability (in short and long 0*g* exposures) but reversed expression changes in a significant proportion of the genes affected by microgravity. PTHrP_1-36_ also reversed many, but a lesser number, of the changes in gene expression modified by *Pthrp* ablation. In that respect, it must be noted that PTHrP_1-36_ will impact genes controlled by the N-terminus of PTHrP, and likely not genes controlled by the protein’s mid-region and C-terminus. Furthermore, reversal responses are likely to be dependent on PTHrP_1-36_ concentration, and a dose-response curve would need to be examined for its influence on genes not affected here which might be modified by higher concentrations of PTHrP_1-36_.

Of particular interest are the genes whose expression change in 0*g* was reversed by intermittent PTHrP_1-36_ treatment as their activity is clearly linked to the maintenance of viability obtained in wild-type TOs treated during long periods at 0*g*. The 74 genes (24 + 50 from central intersects in Fig 5C and 5D, common to ablation and 0*g* and reversed by PTHrP_1-36_) belong to similar clustering categories as above. Among the reversals caused by treatment, some strong candidates for microgravity-induced osteoporotic involvement appear. Two genes (*Mrpplf3* and *Plf2*) that belong to the prolactin family were among the most highly-upregulated. Prolactins are hormones secreted by the pituitary gland and some extra-pituitary tissues. Mitogen-regulated protein/proliferin (MRP/PLF) proteins are members of the prolactin gene family involved in developmental processes. Prolactins have pleiotrophic actions in lactation and many other biological functions, and their expression is regulated by PTHrP [52, 53]. Exposure of pregnant rats to prolactins causes reduced ossification in pups [54], and abnormally high prolactin expression encountered in human conditions such as hyperprolactinemic hypogonadism, extended lactation, or as a consequence of the use of some anti-psychotic drugs, is associated with acute skeletal loss [55–57]. Our results indicate that exposure to 0*g* strikingly (by 12-fold) upregulates expression of prolactin genes *Mrpplf3* and *Plf2*, with highly-efficient reversal by PTHrP_1-36_. Our observations agree with reports that Spaceflight increases prolactin levels in rats [58], and that exposure of healthy male volunteers to head-down bed rest causes significant elevation of prolactin levels [59]. Our results demonstrate expression of prolactin genes in osteoblastic cells and implicate their products in a major bone loss event that is PTHrP-controlled and unrelated to lactation.

The most highly-downregulated gene by both 0*g* and *Pthrp* ablation with reversal by PTHrP_1-36_ is *Rbm3* which encodes RNA-binding motif protein 3, an inhibitor of apoptosis [60, 61]. Its expression is upregulated by PTHrP_1-36_ which prevents TO death, suggesting *Rbm3* involvement in loss of viability and cell death observed at 0g and after ablation. Several genes encoding proteins involved in bone growth, mineralization, BMP and extracellular matrix metabolism are significantly upregulated (*Tnfrsf11b*, *Bdnf*, *Timp3*, *Cryab*, *Grem1*, *Inhba*, *Mustn1*, *Scx*) or downregulated (*Ptn*, *Itm2a*, *Lum*, *Dcn*, *Mfap2*, *Ctsc*, *Col3a1*, *Nid2*) by both 0*g* and *Pthrp* ablation. Of interest among others, the highly-downregulated *Ptn* gene encodes pleitrophin, an osteoblast-secreted matrix-associated heparin-binding protein which enhances osteogenic differentiation of bone marrow cells [62]. *Tnfrsf11b* which encodes osteoprotegerin, the decoy to the receptor activator of nuclear factor kappa-B ligand (RANKL), regulates bone mass by inhibiting osteoclast differentiation and activation. It was found in our study to be strongly upregulated by both microgravity and *Pthrp* ablation in trabecular osteoblasts but downregulated by 0*g* in calvarial osteoblasts [63], suggesting a role in TO and CO different behaviors in microgravity. Also strongly upregulated is *Timp3* (encoding tissue inhibitor of metalloprotease 3) which regulates ECM calcium deposition, and whose overexpression prevents osteoblast differentiation [64]. Gremlin overexpression is known to cause osteopenia and conditional deletion increases bone mass [65, 66], in agreement with our observation that 0g and ablation upregulate *Grem1*. Members of the IGF and chemokine families are downregulated by 0*g* and *Pthrp* ablation with reversal by PTHrP_1-36_. *Igf2* increases bone formation and bone apposition rates [67, 68], and *Igfbp5* stimulates osteoblast proliferation [69]. Chemokine ligand Cxcl12 expression accompanies osteoblast differentiation [70] and its downregulation suggests loss of osteoblast characteristics. All of the above changes point to rapid and profound modifications in trabecular osteoblast metabolism in 0*g* conditions.

The fact that bone loss in flight personnel is reversible to a certain extent upon return to Earth [5, 71, 72] indicates a major difference between Space-induced secondary osteoporosis and the primary aging-associated osteoporosis which is largely irreversible in absence of pharmacologic intervention. It is also interesting to note that in genome-wide association studies, genes associated with aging-related BMD loss do not overlap with those highlighted in our study except for PTHrP [73], osteoprotegerin [74], and *Ptn* [75, 76] suggesting mechanistic differences in bone loss control between these two types of osteoporosis.

## Conclusions

In this study, we present *in vitro* evidence that naturally-expressed PTHrP in TOs has an important anti-apoptotic function in weightless conditions. Our results confirm a survival role for PTHrP in microgravity-exposed osteoblasts, and identify molecules of interest for further investigation in order to find efficient targets for prevention of bone loss due to skeletal unloading. In particular, the involvement of members of the prolactin family suggests a novel mechanistic hypothesis for transmission of the weightlessness stimulus. The role of PTHrP in maintaining low levels of prolactins may be crucial to prevent microgravity-induced osteoporosis and, by extension, disuse osteoporosis.

## Supporting Information

S1 Fig Matrix table (pair-wise combination) of common genes. The 4 genes downregulated by both 0*g* and PTHrP_1-36_ treatment are *Fos*, *Zfp36*, *Pvrlz* and *JunB*.
(TIF)

S1 Table Summary of experimental conditions aboard satellite Foton M3. Pre-programmed valves allowed 5 ml (or less) volumes to be fed through the bioreactors. Each PTHrP_1-36_ treatment was preceded by a 1-min flush, and the 2-h treatment was followed by 2 growth medium rinses and an overnight growth medium feed.
(PDF)

S2 Table Complete list of wild-type trabecular osteoblast genes (total 188) whose expression is modified by exposure to 6 days of simulated microgravity (*Pthrp* ^+/+^ at 1g compared to *Pthrp* ^+/+^ at 0g). **(A)** Genes (total 59) upregulated by 0g (6 days). Fold change > 2.0, p < 0.05. **(B)** Genes (total 129) downregulated by 0g (6 days). Fold change < 0.5, p value< 0.05. All probes: *Mus musculus*.
(PDF)

S3 Table Complete list of trabecular osteoblasts genes (total 763) whose expression is modified by *Pthrp* gene ablation (*Pthrp* ^+/+^ at 1g compared to Pthrp^-/-^ at 1g). **(A)** Genes (total 270) upregulated by *Pthrp* ablation at 1 g. Fold change > 2.0, p < 0.05. **(B)** Genes (total 493) downregulated by *Pthrp* ablation at 1g. Fold change < 0.5, p value< 0.05. All probes: *Mus musculus*.
(PDF)

S4 Table Complete list of trabecular osteoblast genes whose expression is modified by intermittent PTHrP_1-36_ treatment (total 163 genes). **(A)** Genes (total 102) upregulated by intermittent PTHrP_1-36_ treatment. Fold change > 1.5, p < 0.05. **(B)** Genes (total 61) downregulated by treatment. Fold changes < 0.66, p < 0.05.
(PDF)
