# Supplementary figures and images for: The Role of Parathyroid Hormone-Related Protein (PTHrP) in Osteoblast Response to Microgravity: Mechanistic Implications for Osteoporosis Development
Source: PLoS One. 2016 Jul 27;11(7):e0160034. doi: 10.1371/journal.pone.0160034 (PMC4963112; doi:10.1371/journal.pone.0160034)

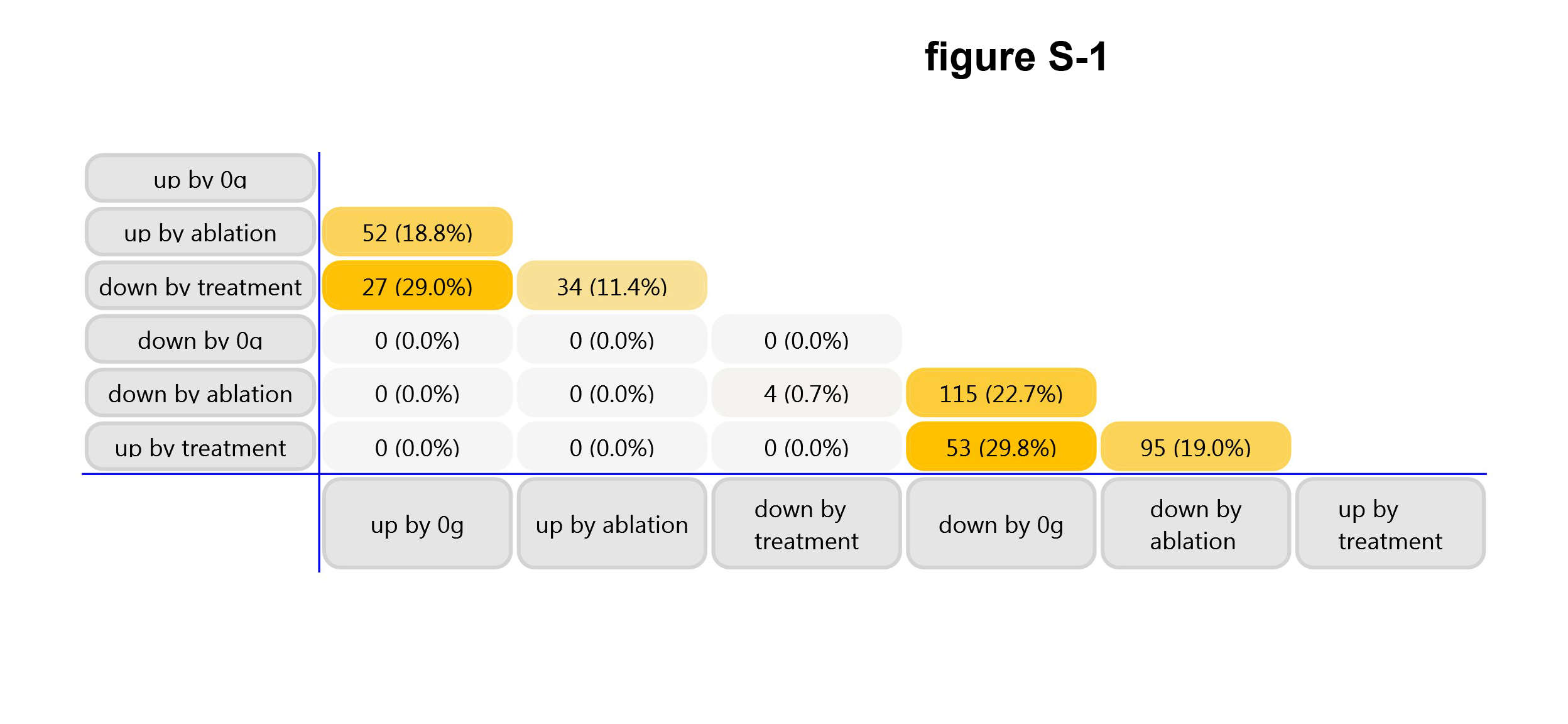

Supplement: S1 Fig — The 4 genes downregulated by both 0g and PTHrP1-36 treatment are Fos, Zfp36, Pvrlz and JunB. (TIF) [file pone.0160034.s001.tif]
