# Supplementary material for: The Role of Parathyroid Hormone-Related Protein (PTHrP) in Osteoblast Response to Microgravity: Mechanistic Implications for Osteoporosis Development
Source: PLoS One. 2016 Jul 27;11(7):e0160034. doi: 10.1371/journal.pone.0160034 (PMC4963112; doi:10.1371/journal.pone.0160034)
Supplement: S1 Table — Pre-programmed valves allowed 5 ml (or less) volumes to be fed through the bioreactors. Each PTHrP1-36 treatment was preceded by a 1-min flush, and the 2-h treatment was followed by 2 growth medium rinses and an overnight growth medium feed. (PDF) [file pone.0160034.s002.pdf]

	Process number	Date L: launch	Hour	Minute	Growth Medium (ml)	PTHrP in Medium (ml)	Fixative (ml)	PBS (ml)
Day 1	1	L + 1	1	1	0	3	0	0
	2		1	2	0	5	0	0
	3		3	3	5	0	0	0
	4		3	4	5	0	0	0
	5		3	5	5	0	0	0
Day 2	1	L + 2	1	1	0	3	0	0
	2		1	2	0	5	0	0
	3		3	3	5	0	0	0
	4		3	4	5	0	0	0
	5		3	5	5	0	0	0
Day 3	1	L + 3	1	1	0	3	0	0
	2		1	2	0	5	0	0
	3		3	3	5	0	0	0
	4		3	4	5	0	0	0
	5		3	5	5	0	0	0
Day 4	1	L + 4	1	1	0	3	0	0
	2		1	2	0	5	0	0
	3		3	3	5	0	0	0
	4		3	4	5	0	0	0
	5		3	5	5	0	0	0
Day 5	1	L + 5	1	1	0	3	0	0
	2		1	2	0	5	0	0
	3		3	3	5	0	0	0
	4		3	4	5	0	0	0
	5		3	5	5	0	0	0
Day 6	1	L + 6	1	1	0	3	0	0
	2		1	2	0	5	0	0
	3		3	3	5	0	0	0
	4		3	4	5	0	0	0
	5		3	5	5	0	0	0
Day 7	1	L + 7	0	1	0	0	0	5
	2		0	2	0	0	0	5
	3		0	3	0	0	0	5
	4		0	4	0	0	0	5
	5		2	5	0	0	5	0
	6		2	6	0	0	5	0
	7		2	7	0	0	5	0
	8		2	8	0	0	5	0
	9		2	9	0	0	0	5
	10		2	10	0	0	0	5
	11		2	11	0	0	0	5
	12		2	12	0	0	0	5
	13		2	13	0	0	0	5
