# Supplementary material for: The Role of Parathyroid Hormone-Related Protein (PTHrP) in Osteoblast Response to Microgravity: Mechanistic Implications for Osteoporosis Development
Source: PLoS One. 2016 Jul 27;11(7):e0160034. doi: 10.1371/journal.pone.0160034 (PMC4963112; doi:10.1371/journal.pone.0160034)
Supplement: S2 Table — (A) Genes (total 59) upregulated by 0g (6 days). Fold change > 2.0, p < 0.05. (B) Genes (total 129) downregulated by 0g (6 days). Fold change < 0.5, p value< 0.05. All probes: Mus musculus. (PDF) [file pone.0160034.s003.pdf]

S2 A and B Table: Genes affected by 6 days of simulated microgravity

S2 A Table
Genes (59) upregulated by 6 days of simulated microgravity (fold change > 2.0, p < 0.05)

Target ID	Definition (all probes : *Mus musculus*)	RefSeq ID	Fold change	Upregulated by ablation
PLF2	Mus musculus prolactin family 2, subfamily c, member 3 (Prl2c3), mRNA.	NM_011118.1	12.81098	*
MRPPLF3	Mus musculus prolactin family 2, subfamily c, member 4 (Prl2c4), mRNA.	NM_011954.2	11.76853	*
AQP5	Mus musculus aquaporin 5 (Aqp5), mRNA.<br>PREDICTED: Mus musculus similar to aquaporin 5 (LOC100046616), mRNA.	NM_009701.4<br>XM_001476512.1	7.790976<br>3.627969	*
CCK	Mus musculus cholecystokinin (Cck), mRNA.	NM_031161.2	5.513795	No
INHBB	PREDICTED: Mus musculus similar to Inhbb protein (LOC100046802), mRNA.	XM_001476835.1	4.413428	No
GREM1	Mus musculus gremlin1 (Grem1), mRNA	NM_011824.1	4.151464	*
GPC1	Mus musculus glypican 1 (Gpc1), mRNA.	NM_016696.3	4.13393	*
ALDH3A1	Mus musculus aldehyde dehydrogenase family 3, subfamily A1 (Aldh3a1), mRNA.	NM_007436.1	3.821048	*
ASS1	Mus musculus argininosuccinate synthetase 1 (Ass1), mRNA.	NM_007494.3	3.663361	*
NKD2	Mus musculus naked cuticle 2 homolog (Drosophila) (Nkd2), mRNA.	NM_028186.3	3.63063	*
NQO1	Mus musculus NAD(P)H dehydrogenase, quinone 1 (Nqo1), mRNA.	NM_008706.4	3.439677	*
INHBA	Mus musculus inhibin beta-A (Inhba), mRNA.	NM_008380.1	3.374437	*
COLM	Mus musculus gliomedin (Gldn), mRNA.	NM_177350.4	3.366197	No
HIST1H4H	Mus musculus histone cluster 1, H4h (Hist1h4h), mRNA.	NM_153173.2	3.122563	*
HSPB1	Mus musculus heat shock protein 1 (Hspb1), mRNA.	NM_013560.1	2.937195	*
CRIP2	Mus musculus cysteine rich protein 2 (Crip2), mRNA.	NM_024223.1	2.87678	*
TIMP3	Mus musculus tissue inhibitor of metalloproteinase 3 (Timp3), mRNA.	NM_011595.2<br>NM_011595.2	2.833766<br>2.168426	*
PRSS19	Mus musculus kallikrein related-peptidase 8 (Klk8), mRNA.	NM_008940.2	2.649386	No
ESD	Mus musculus esterase D/formylglutathione hydrolase (Esd), mRNA.	NM_016903.2	2.617979	*
PRELP	Mus musculus proline arginine-rich end leucine-rich repeat (Prelp), mRNA.	NM_054077.3	2.563206	*
1110032E23RIK	Mus musculus RIKEN cDNA 1110032E23 gene (1110032E23Rik), mRNA.	NM_133187.2	2.532211	*
GDF15	Mus musculus growth differentiation factor 15 (Gdf15), mRNA.	NM_011819.1	2.506674	*
FOSB	Mus musculus FBJ osteosarcoma oncogene B (Fosb), mRNA.	NM_008036.2	2.464137	No
AHRR	Mus musculus aryl-hydrocarbon receptor repressor (Ahrr), mRNA.	NM_009644.2	2.366026	*
NBL1	Mus musculus neuroblastoma, suppression of tumorigenicity 1 (Nbl1), mRNA.	NM_008675.1	2.362421	*
CRYAB	Mus musculus crystallin, alpha B (Cryab), mRNA.	NM_009964.1	2.332638	*
FABP3	Mus musculus fatty acid binding protein 3, muscle and heart (Fabp3), mRNA.	NM_010174.1	2.32906	*
MDM2	Mus musculus transformed mouse 3T3 cell double minute 2 (Mdm2), mRNA.	NM_010786.2<br>NM_010786.3	2.31834<br>2.246531	*
KCTD10	Mus musculus potassium channel tetramerisation domain containing 10 (Kctd10), mRNA.	NM_026145.3	2.279135	*
SGK	Mus musculus serum/glucocorticoid regulated kinase 1 (Sgk1), mRNA.	NM_011361.1	2.240079	*
ANK	Mus musculus progressive ankylosis (Ank), mRNA.	NM_020332.3	2.228785	*
EPHX1	Mus musculus epoxide hydrolase 1, microsomal (Ephx1), mRNA.	NM_010145.2	2.215451	*
WHRN	Mus musculus whirlin (Whrn), transcript variant 3, mRNA.	NM_001008792.1	2.202689	*
CD44	Mus musculus CD44 antigen (Cd44), transcript variant 2, mRNA.	NM_001039150.1	2.195092	*
CTGF	Mus musculus connective tissue growth factor (Ctgf), mRNA.	NM_010217.1	2.193812	*
SCX	Mus musculus scleraxis (Scx), mRNA.	NM_198885.2	2.185468	*

MUSTN1	Mus musculus musculoskeletal, embryonic nuclear protein 1 (Mustn1), mRNA.	NM_181390.1<br>NM_181390.2	2.185364<br>2.109783	*
HIST1H2BJ	Mus musculus histone cluster 1, H2bj (Hist1h2bj), mRNA.	NM_178198.1	2.173656	*
EEF1A2	Mus musculus eukaryotic translation elongation factor 1 alpha 2 (Eef1a2), mRNA.	NM_007906.2<br>NM_007906.2	2.153269<br>2.025144	*
TNFRSF11B	Mus musculus tumor necrosis factor receptor superfamily, member 11b (osteoprotegerin) (Tnfrsf11b), mRNA.	NM_008764.3	2.129809	*
HOXC6	Mus musculus homeo box C6 (Hoxc6), mRNA.	NM_010465.2	2.107019	*
AEBP1	Mus musculus AE binding protein 1 (Aebp1), mRNA.	NM_009636.1	2.102786	No
2300002D11RIK	Mus musculus RIKEN cDNA 2300002D11 gene (2300002D11Rik), mRNA.	NM_001081156.1	2.097322	*
BDNF	Mus musculus brain derived neurotrophic factor (Bdnf), transcript variant 3, mRNA.	NM_001048141.1	2.091327	*
HSPB8	Mus musculus heat shock protein 8 (Hspb8), mRNA.	NM_030704.1	2.090601	*
PDGFA	Mus musculus platelet-derived growth factor A type receptor (Pdgfa), mRNA.		2.084386	*
CCNG1	Mus musculus cyclin G1 (Ccng1), mRNA.	NM_009831.2	2.083259	*
AKR1C18	Mus musculus aldo-keto reductase family 1, member C18 (Akr1c18), mRNA.	NM_134066.2	2.073836	No
HIST1H2BM	Mus musculus histone cluster 1, H2bm (Hist1h2bm), mRNA.	NM_178200.1	2.072647	*
HIST1H2BH	Mus musculus histone cluster 1, H2bh (Hist1h2bh), mRNA.	NM_178197.1	2.069317	*
HIST1H2BF	Mus musculus histone cluster 1, H2bf (Hist1h2bf), mRNA.	NM_178195.1	2.066524	*
COL7A1	Mus musculus collagen, type VII, alpha 1 (Col7a1), mRNA.	NM_007738.3	2.044317	*
A630005A06RIK	Mus musculus TBC1 domain family, member 2 (Tbc1d2), mRNA.	NM_198664.3	2.044002	*
GSTP1	Mus musculus glutathione S-transferase, pi 1 (Gstp1), mRNA.	NM_013541.1	2.039079	*
TNFRSF12A	Mus musculus tumor necrosis factor receptor superfamily, member 12a (Tnfrsf12a), mRNA.	NM_013749.1	2.026671	*
SNRPN	Mus musculus SNRPN upstream reading frame (Snurf), mRNA.	NM_033174.2	2.014161	*
HIST1H2BK	Mus musculus histone cluster 1, H2bk (Hist1h2bk), mRNA.	NM_175665.1	2.012974	*
STMN2	Mus musculus stathmin-like 2 (Stmn2), mRNA.	NM_025285.2	2.007183	*
DOS	Mus musculus downstream of Stk11 (Dos), mRNA.	NM_015761.2	2.00261	*

S2 B Table
Genes (129) downregulated by 6 days of simulated microgravity (fold change< 0.5, $p < 0.05$)

Target ID	Definition (all probes : *Mus musculus*)	RefSeq ID	Fold change	Down-regulated by ablation
SFRP2	Mus musculus secreted frizzled-related protein 2 (Sfrp2), mRNA.	NM_009144.1	0.1316429	*
PTN	Mus musculus pleiotrophin (Ptn), mRNA.	NM_008973.2	0.1557338	*
IGFBP5	Mus musculus insulin-like growth factor binding protein 5 (Igfbp5), mRNA.	NM_010518.2	0.1603545	*
1500015O10RIK	Mus musculus RIKEN cDNA 1500015O10 gene (1500015O10Rik), mRNA.	NM_024283.2	0.1804316	*
IGF2	Mus musculus insulin-like growth factor 2 (Igf2), mRNA.	NM_010514.2	0.2085582	*
CLU	PREDICTED: Mus musculus similar to clusterin (LOC100046120), mRNA.	XM_001475611.1	0.213665	*
CXCL12	Mus musculus chemokine (C-X-C motif) ligand 12 (Cxcl12), transcript variant 1, mRNA.	NM_013655.2<br>NM_021704.2<br>NM_001012477.1	0.2150201<br>0.2234856<br>0.236011	*
HP	Mus musculus haptoglobin (Hp), mRNA.	NM_017370.1<br>NM_017370.1<br>NM_017370.1	0.2222102<br>0.4034571<br>0.4659876	*

C3	Mus musculus mouse complement 3, mRNA.	NM_009778.1	0.2325967	*
SCARA5	Mus musculus scavenger receptor class A, member 5 (putative) (Scara5), mRNA.	NM_028903.1	0.2351416	*
LUM	Mus musculus lumican (Lum), mRNA.	NM_008524.1	0.235224	*
MRC1	Mus musculus mannose receptor, C type 1 (Mrc1), mRNA.	NM_008625.1	0.253038	*
4930583H14RIK	Mus musculus RIKEN cDNA 4930583H14 gene (4930583H14Rik), mRNA.	NM_026358.2	0.2575598	*
2310061N23RIK	Mus musculus interferon, alpha-inducible protein 27 (Ifi27), mRNA.	NM_029803.1	0.2600992	*
DCN	Mus musculus decorin (Dcn), mRNA.	NM_007833.4<br>NM_007833.4	0.2889794<br>0.3837174	*
H19	Mus musculus H19 fetal liver mRNA (H19), non-coding RNA.	NR_001592.1	0.2906019	*
PPP1R3C	Mus musculus protein phosphatase 1, regulatory (inhibitor) subunit 3C (Ppp1r3c), mRNA.	NM_016854.2	0.2954025	*
RBM3	PREDICTED: Mus musculus similar to RNA binding motif protein 3 (LOC100043257), mRNA.	XM_001480197.1	0.2991486	*
APOD	PREDICTED: Mus musculus similar to apolipoprotein D (LOC100047583), mRNA.	XM_001479138.1	0.2996465	*
ITM2A	Mus musculus integral membrane protein 2A (Itm2a), mRNA.	NM_008409.2	0.2997355	*
D930038M13RIK	Mus musculus ABI gene family, member 3 (NESH) binding protein (Abi3bp), transcript variant 1, mRNA.	NM_178790.3	0.3036303	*
PFKL	Mus musculus phosphofructokinase, liver, B-type (Pfkl), mRNA.	NM_008826.3	0.3054363	*
BMP4	Mus musculus bone morphogenetic protein 4 (Bmp4), mRNA.	NM_007554.2	0.3117725	No
OLFML1	Mus musculus olfactomedin-like 1 (Olfml1), mRNA.	NM_172907.2	0.3123395	*
HIST1H2AD	Mus musculus histone cluster 1, H2ad (Hist1h2ad), mRNA.	NM_178188.3	0.3149356	*
DLK1	Mus musculus delta-like 1 homolog (Drosophila) (Dlk1), mRNA.	NM_010052.4	0.3273854	*
HIST1H2AK	Mus musculus histone cluster 1, H2ak (Hist1h2ak), mRNA.	NM_178183.1	0.3372404	*
2310056P07RIK	Mus musculus family with sequence similarity 162, member A (Fam162a), mRNA.	NM_027342.1	0.3391683	*
HIST1H2AF	Mus musculus histone cluster 1, H2af (Hist1h2af), mRNA.	NM_175661.1	0.3396376	*
SLC1A3	Mus musculus solute carrier family 1 (glial high affinity glutamate transporter), member 3 (Slc1a3), mRNA.	NM_148938.2	0.3399492	*
PSCDBP	Mus musculus cytohesin 1 interacting protein (Cytip), mRNA.	NM_139200.4	0.3403133	*
D0H4S114	Mus musculus DNA segment, human D4S114 (D0H4S114), mRNA.	NM_053078.3	0.3474809	No
NID2	Mus musculus nidogen 2 (Nid2), mRNA.	NM_008695.2	0.3477556	*
TGFBI	Mus musculus transforming growth factor, beta induced (Tgfbi), mRNA.	NM_009369.1	0.3514812	*
HIST1H2AH	Mus musculus histone cluster 1, H2ah (Hist1h2ah), mRNA.	NM_175659.1	0.352093	*
FMO1	Mus musculus flavin containing monooxygenase 1 (Fmo1), mRNA.	NM_010231.2	0.3568025	*
EGLN3	Mus musculus EGL nine homolog 3 (C. elegans) (Egln3), mRNA.	NM_028133.1	0.3569504	*
DPEP2	Mus musculus dipeptidase 2 (Dpep2), mRNA.	NM_176913.3<br>NM_176913.3	0.3577472<br>0.3965119	*
COL3A1	Mus musculus collagen, type III, alpha 1 (Col3a1), mRNA.	NM_009930.1	0.3599768	*
1200009O22RIK	Mus musculus RIKEN cDNA 1200009O22 gene (1200009O22Rik), mRNA.	NM_025817.3	0.3616481	*
HIST1H2AO	Mus musculus histone cluster 1, H2ao (Hist1h2ao), mRNA.	NM_178185.1	0.3618695	*
AGTR1A	Mus musculus angiotensin II receptor, type 1a (Agtr1a), mRNA.	NM_177322.2	0.3640873	*
LDB2	Mus musculus LIM domain binding protein (Ldb2), mRNA	NM_010698.2	0.3694307	*
DPT	Mus musculus dermatopontin (Dpt), mRNA.	NM_019759.2	0.369822	*
6330406I15RIK	Mus musculus RIKEN cDNA 6330406I15 gene (6330406I15Rik), mRNA.	NM_027519.1	0.3718243	*
RASSF4	Mus musculus Ras association (RalGDS/AF-6) domain family member 4 (Rassf4), mRNA.	NM_178045.3<br>NM_178045.3	0.372338<br>0.3845508	*
PDGFRA	Mus musculus platelet derived growth factor receptor, alpha polypeptide (Pdgfra), transcript variant 1, mRNA.	NM_011058.2	0.3755132	*
HIST1H2AN	Mus musculus histone cluster 1, H2an (Hist1h2an), mRNA.	NM_178184.1	0.3776532	*

ZCCHC5	Mus musculus zinc finger, CCHC domain containing 5 (Zcchc5), mRNA.	NM_199468.1	0.3839341	No
SCARA3	Mus musculus scavenger receptor class A, member 3 (Scara3), mRNA.	NM_172604.3	0.3876348	No
IGFBP3	Mus musculus insulin-like growth factor binding protein 3 (Igfbp3), mRNA.	NM_008343.2	0.3878084	*
AOC3	Mus musculus amine oxidase, copper containing 3 (Aoc3), mRNA.	NM_009675.1	0.390921	*
KNG1	Mus musculus kininogen 1 (Kng1), mRNA.	NM_023125.2	0.3914529	*
SERPING1	Mus musculus serine (or cysteine) peptidase inhibitor, clade G, member 1 (Serping1), mRNA.	NM_009776.1	0.3939361	*
SFRP1	Mus musculus secreted frizzled-related protein 1 (Sfrp1), mRNA.	NM_013834.1	0.3966504	*
6330406I15 RIK	Mus musculus RIKEN cDNA 6330406I15 gene (6330406I15Rik), mRNA.	NM_027519.3	0.3991528	*
IGFBP4	Mus musculus insulin-like growth factor binding protein 4 (Igfbp4)	NM_010517.2	0.3999539	No
EMR1	Mus musculus EGF-like module containing, mucin-like, hormone receptor-like sequence 1 (Emr1), mRNA.	NM_010130.3<br>NM_010130.1	0.4002873<br>0.4839607	*
SIRPB1	Mus musculus signal-regulatory protein beta 1 (Sirpb1), transcript variant 3, mRNA.	NM_001002898.1	0.4006563	*
CAR9	Mus musculus carbonic anhydrase 9 (Car9), mRNA.	NM_139305.1	0.401926	*
SLC24A3	Mus musculus solute carrier family 24 (sodium/potassium/calcium exchanger), member 3 (Slc24a3), mRNA.	NM_053195.2	0.4027135	No
SRPX	Mus musculus sushi-repeat-containing protein (Srpx), mRNA.	NM_016911.4<br>NM_016911.4	0.4043711<br>0.4169736	*
LBP	Mus musculus lipopolysaccharide binding protein (Lbp), mRNA.	NM_008489.2	0.4046987	*
2310006J04 RIK	Mus musculus ankyrin repeat domain 37 (Ankrd37), mRNA.	NM_001039562.1	0.4075139	No
CCR5	Mus musculus chemokine (C-C motif) receptor 5 (Ccr5), mRNA.	NM_009917.2<br>NM_009917.2<br>NM_001008702.1	0.4091699<br>0.4434175<br>0.4461237	*
RAMP2	Mus musculus receptor (calcitonin) activity modifying protein 2 (Ramp2), mRNA.	NM_019444.2	0.4105468	*
LSP1	PREDICTED: Mus musculus predicted gene, ENSMUSG00000043795 (ENSMUSG00000043795), mRNA.	XM_001480835.1	0.4114556	*
CDKN1C	Mus musculus cyclin-dependent kinase inhibitor 1C (P57) (Cdkn1c), mRNA.	NM_009876.3	0.4116614	*
CORO1A	Mus musculus coronin, actin binding protein 1A (Coro1a), mRNA.	NM_009898.2<br>NM_009898.2<br>NM_009898.2	0.4127754<br>0.4435945<br>0.4970813	*
C1QA	Mus musculus complement component 1, q subcomponent, alpha polypeptide (C1qa), mRNA.	NM_007572.2	0.4128893	*
ELN	Mus musculus elastin (Eln), mRNA.	NM_007925.3	0.4149198	No
SLC2A1	Mus musculus solute carrier family 2 (facilitated glucose transporter), member 1 (Slc2a1), mRNA.	NM_011400.2	0.4167042	*
GPR23	Mus musculus G protein-coupled receptor 23 (Gpr23), mRNA.	NM_175271.2	0.4254305	*
PDK1	Mus musculus pyruvate dehydrogenase kinase, isoenzyme 1 (Pdk1), nuclear gene encoding mitochondrial protein, mRNA.	NM_172665.3	0.4255101	*
CTSC	Mus musculus cathepsin C (Ctsc), mRNA.	NM_009982.2	0.4265272	*
OASL2	Mus musculus 2'-5' oligoadenylate synthetase-like 2 (Oasl2), mRNA.	NM_011854.1	0.4272569	*
C1QB	Mus musculus complement component 1, q subcomponent, beta polypeptide (C1qb), mRNA.	NM_009777.2	0.4278048	*
DAB2	Mus musculus disabled homolog 2 (Drosophila) (Dab2), transcript variant 2, mRNA.	NM_023118.1	0.4278204	*
RSPO3	Mus musculus R-spondin 3 homolog (Xenopus laevis) (Rspo3), mRNA.	NM_028351.2	0.4293909	*
MFAP2	Mus musculus microfibrillar-associated protein 2 (Mfap2), mRNA.	NM_008546.2<br>NM_008546.2<br>NM_008546.2	0.4296708<br>0.4759155<br>0.4945922	*
GMFG	Mus musculus glia maturation factor, gamma (Gmfg), transcript variant 1, mRNA.	NM_022024.2<br>NM_022024.2	0.4302362<br>0.4870403	*
SELENBP1	PREDICTED: Mus musculus hypothetical protein LOC100044204 (LOC100044204), mRNA.	XM_001471696.1	0.433163	*
CAPN6	Mus musculus calpain 6 (Capn6), mRNA.	NM_007603.2	0.4346417	No
MEST	Mus musculus mesoderm specific transcript (Mest), mRNA.	NM_008590.1<br>NM_008590.1	0.4349252<br>0.4556044	*

MME	Mus musculus membrane metallo endopeptidase (Mme), mRNA.	NM_008604.2	0.4350618	*
NDRG1	Mus musculus N-myc downstream regulated gene 1 (Ndrg1), mRNA.	NM_008681.2	0.4352534	No
MS4A6D	Mus musculus membrane-spanning 4-domains, subfamily A, member 6D (Ms4a6d), mRNA.	NM_026835.2	0.4373044	*
ALOX5AP	Mus musculus arachidonate 5-lipoxygenase activating protein (Alox5ap), mRNA.	NM_009663.1	0.4387523	*
HIST1H2AG	Mus musculus histone cluster 1, H2ag (Hist1h2ag), mRNA.	NM_178186.2	0.4395184	*
1300002F13RIK	Mus musculus ERBB receptor feedback inhibitor 1 (Errfi1), mRNA.	NM_133753.1	0.4395548	*
EVI2A	Mus musculus ecotropic viral integration site 2a (Evi2a), transcript variant 2, mRNA.	NM_010161.3	0.4402088	*
CXCL4	Mus musculus chemokine (C-X-C motif) ligand 4 (Cxcl4), mRNA.	NM_019932.2	0.4419611	*
1190002H23RIK	Mus musculus RIKEN cDNA 1190002H23 gene (1190002H23Rik), mRNA.	NM_025427.2	0.4430251	*
DAB2	Mus musculus disabled homolog 2 (Drosophila) (Dab2), transcript variant 2, mRNA.	NM_001008702.1	0.4450885	*
ENO1	Mus musculus enolase 1, alpha non-neuron (Eno1), mRNA.	NM_023119.1	0.4461543	*
LYZ	Mus musculus lysozyme 1 (Lyz1), mRNA.	NM_013590.3	0.4473266	*
CD52	Mus musculus CD52 antigen (Cd52), mRNA.	NM_013706.1	0.44963	*
NDRL	Mus musculus N-myc downstream gene (Ndrl), mRNA.		0.4504366	*
CYP7B1	Mus musculus cytochrome P450, family 7, subfamily b, polypeptide 1 (Cyp7b1), mRNA.	NM_007825.3	0.450876	*
PPP3CA	Mus musculus protein phosphatase 3, catalytic subunit, alpha isoform (Ppp3ca), mRNA.	NM_008913.1	0.4521215	No
PIRA4	Mus musculus paired-Ig-like receptor A4 (Pira4), mRNA.	NM_011091.1	0.4527747	*
RARRES2	Mus musculus retinoic acid receptor responder (tazarotene induced) 2 (Rarres2), mRNA.	NM_027852.1	0.4537163	*
CD14	Mus musculus CD14 antigen (Cd14), mRNA.	NM_009841.3	0.4582975	*
ODZ4	Mus musculus odd Oz/ten-m homolog 4 (Drosophila) (Odz4), mRNA.	NM_011858.3	0.4619088	No
ALDH3B1	Mus musculus aldehyde dehydrogenase 3 family, member B1 (Aldh3b1), mRNA.	NM_026316.2	0.4627661	*
OGN	Mus musculus osteoglycin (Ogn), mRNA.	NM_008760.2	0.4635047	No
KCNAB2	Mus musculus potassium voltage-gated channel, shaker-related subfamily, beta member 2 (Kcnab2), mRNA.	NM_010598.2	0.4659567	*
C1QG	Mus musculus complement component 1, q subcomponent, C chain (C1qc), mRNA.	NM_007574.2	0.468035	*
GALNT9	Mus musculus UDP-N-acetyl-alpha-D-galactosamine:polypeptide N-acetylgalactosaminyltransferase 9 (Galnt9), mRNA.	NM_198306.1	0.4683551	*
ADD3	Mus musculus adducin 3 (gamma) (Add3), mRNA.	NM_013758.2	0.4687887	*
ZFP36L1	Mus musculus zinc finger protein 36, C3H type-like 1 (Zfp36l1), mRNA.	NM_007564.4	0.4703194	*
HEMP1	Mus musculus NCK associated protein 1 like (Nckap1l), mRNA.	NM_153505.4	0.4709411	*
LGMN	Mus musculus legumain (Lgmn), mRNA.	NM_011175.2	0.4770529	*
2810046M22RIK		NM_026621.1	0.4778584	No
TXNIP	Mus musculus thioredoxin interacting protein (Txnip), transcript variant 1, mRNA.	NM_001009935.2	0.4827267	*
EHD4	Mus musculus EH-domain containing 4 (Ehd4), mRNA.	NM_133838.4	0.483588	*
LDH1	Mus musculus lactate dehydrogenase A (Ldha), mRNA.	NM_010699.1	0.4844018	No
ARHGAP20	Mus musculus Rho GTPase activating protein 20 (Arhgap20), mRNA.	NM_175535.3	0.4859805	*
PTX3	Mus musculus pentaxin related gene (Ptx3) mRNA.	NM_008987.2	0.4867727	*
ENTPD2	Mus musculus ectonucleoside triphosphate diphosphohydrolase 2 (Entpd2), mRNA.	NM_009849.1	0.4874467	No
NCF4	Mus musculus neutrophil cytosolic factor 4 (Ncf4), mRNA.	NM_008677.1	0.4881307	*
PFC	Mus musculus complement factor properdin (Cfp), mRNA.	NM_008823.3	0.488737	*
SMARCA1	Mus musculus SWI/SNF related, matrix associated, actin dependent regulator of chromatin, subfamily a, member 1 (Smarca1), mRNA.	NM_053123.3	0.4911543	*

FCER1G	Mus musculus Fc receptor IgE high affinity 1 gamma polypeptde (Fcer1g) mRNA	NM_010185.2	0.4915371	*
CENPA	Mus musculus centromere protein A (Cenpa), mRNA.	NM_007681.2	0.4943203	*
MIF	Mus musculus macrophage migration inhibitory factor (Mif), mRNA.	NM_010798.2	0.4948902	No
1200002N14RIK	Mus musculus RIKEN cDNA 1200002N14 gene (1200002N14Rik), mRNA.	NM_027878.2	0.4949315	*
TK1	Mus musculus thymidine kinase 1 (Tk1), mRNA.	NM_009387.1	0.4950169	*
ALDOA	Mus musculus aldolase A, fructose-bisphosphate (Aldoa), mRNA.	NM_007438.3	0.4959253	*
SLPI	Mus musculus secretory leukocyte peptidase inhibitor (Slpi), mRNA.	NM_011414.2	0.4988716	*
DMN	Mus musculus synemin, intermediate filament protein (Synm), transcript variant 3, mRNA.	NM_183312.3	0.4993148	No
