# Supplementary material for: The Role of Parathyroid Hormone-Related Protein (PTHrP) in Osteoblast Response to Microgravity: Mechanistic Implications for Osteoporosis Development
Source: PLoS One. 2016 Jul 27;11(7):e0160034. doi: 10.1371/journal.pone.0160034 (PMC4963112; doi:10.1371/journal.pone.0160034)
Supplement: S3 Table — (A) Genes (total 270) upregulated by Pthrp ablation at 1 g. Fold change > 2.0, p < 0.05. (B) Genes (total 493) downregulated by Pthrp ablation at 1g. Fold change < 0.5, p value< 0.05. All probes: Mus musculus. (PDF) [file pone.0160034.s004.pdf]

S3 A and B Tables: Genes (763) affected by *Pthrp* ablation (at 1g).

S3 A Table: Genes (270) upregulated by *Pthrp* ablation (at 1g) (fold change > 2.0, p < 0.05)

Target ID	Definition (all probes : *Mus musculus*)	RefSeq ID	Fold change	Up regulated by 0g
AQP5	Mus musculus aquaporin 5 (Aqp5), mRNA.<br>PREDICTED: Mus musculus similar to aquaporin 5 (LOC100046616), mRNA.	NM_009701.4<br>XM_001476512.1	25.5869<br>9.54725	*
PLF2	Mus musculus prolactin family 2, subfamily c, member 3 (Prl2c3), mRNA.	NM_011118.1	20.6708	*
MRPPLF3	Mus musculus prolactin family 2, subfamily c, member 4 (Prl2c4), mRNA.	NM_011954.2	19.0465	*
ESM1	Mus musculus endothelial cell-specific molecule 1 (Esm1), mRNA.	NM_023612.3	11.5814	
ANXA8	Mus musculus annexin A8 (Anxa8), mRNA.	NM_013473.3<br>NM_013473.2	11.0903<br>3.9279	
NKD2	Mus musculus naked cuticle 2 homolog (Drosophila) (Nkd2), mRNA.	NM_028186.3	9.96437	*
NQO1	Mus musculus NAD(P)H dehydrogenase, quinone 1 (Nqo1), mRNA.	NM_008706.4	9.41091	*
CRYAB	Mus musculus crystallin, alpha B (Cryab), mRNA.	NM_009964.1	9.13575	*
ANK	Mus musculus progressive ankylosis (Ank), mRNA.	NM_020332.3	9.11502	*
TNFRSF11B	Mus musculus tumor necrosis factor receptor superfamily, member 11b (osteoprotegerin) (Tnfrsf11b), mRNA.	NM_008764.3	9.05821	*
ADH7	Mus musculus alcohol dehydrogenase 7 (class IV), mu or sigma polypeptide (Adh7), mRNA.	NM_009626.3	8.11975	
FABP3	Mus musculus fatty acid binding protein 3, muscle and heart (Fabp3), mRNA.	NM_010174.1	7.70136	*
BDNF	Mus musculus brain derived neurotrophic factor (Bdnf), transcript variant 3, mRNA.	NM_001048141.1	7.46112	*
HIST1H4H	Mus musculus histone cluster 1, H4h (Hist1h4h), mRNA.	NM_153173.2	6.52552	*
FHL1	Mus musculus four and a half LIM domains 1 (Fhl1), transcript variant 1, mRNA.	NM_001077361.1<br>NM_001077362.1	6.46872<br>6.12187	
S3-12	Mus musculus plasma membrane associated protein, S3-12 (S3-12), mRNA.	NM_020568.2	6.38498	
INHBA	Mus musculus inhibin beta-A (Inhba), mRNA.	NM_008380.1	6.03861	*
1110032E23 RIK	Mus musculus RIKEN cDNA 1110032E23 gene (1110032E23Rik), mRNA.	NM_133187.2	5.50103	*
GREM1	Mus musculus gremlin1 (Grem1) mRNA.	NM_011824.1	5.17357	*
CDH13	Mus musculus cadherin 13 (Cdh13), mRNA.	NM_019707.4<br>NM_019707.4	5.05871<br>2.57044	
CD44	Mus musculus CD44 antigen (Cd44), transcript variant 2, mRNA.	NM_001039150.1	4.9279	*
ESD	Mus musculus esterase D/formylglutathione hydrolase (Esd), mRNA.	NM_016903.2<br>NM_016903.2	4.78236<br>2.20099	*
TIMP3	Mus musculus tissue inhibitor of metalloproteinase 3 (Timp3), mRNA.	NM_011595.2<br>NM_011595.2	4.71802<br>4.57388	*
DSCR1	Mus musculus Down syndrome critical region homolog 1 (Dscr1) mRNA.	NM_019466.2	4.5003	
IRX3	Mus musculus Iroquois related homeobox 3 (Drosophila) (Irx3), mRNA.		4.36278	
PDLIM1	Mus musculus PDZ and LIM domain 1 (elfin) (Pdlim1), mRNA.	NM_016861.3	4.35651	
2300002D11 RIK	Mus musculus RIKEN cDNA 2300002D11 gene (2300002D11Rik), mRNA.	NM_001081156.1	4.2774	*
CRIP2	Mus musculus cysteine rich protein 2 (Crip2), mRNA.	NM_024223.1	4.26098	*
SCX	Mus musculus scleraxis (Scx), mRNA.	NM_198885.2	4.25152	*
1810008K03 RIK	Mus musculus ChaC, cation transport regulator-like 1 (E. coli) (Chac1), mRNA.	NM_026929.3	4.23677	
2300002G24 RIK	Mus musculus cysteine-rich C-terminal 1 (Crct1), mRNA.	NM_028798.2	4.20434	
3110050N22 RIK		NM_173181.1	4.07191	

ITGBL1	Mus musculus integrin, beta-like 1 (Itgbl1), mRNA.	NM_145467.1	4.06449	
PHF17	Mus musculus PHD finger protein 17 (Phf17), mRNA.	NM_172303.3	4.03838	
GPC1	Mus musculus glypican 1 (Gpc1), mRNA.	NM_016696.3	4.00833	*
BC099439	Mus musculus cDNA sequence BC099439 (BC099439), mRNA.	NM_001025564.1	4.0077	
MUSTN1	Mus musculus musculoskeletal, embryonic nuclear protein 1 (Mustn1), mRNA.	NM_181390.1<br>NM_181390.2	3.95709<br>3.09965	*
FGF10	Mus musculus fibroblast growth factor 10 (Fgf10), mRNA.	NM_008002.3<br>NM_008002.3	3.9152<br>3.88914	
WNT10A	Mus musculus wingless related MMTV integration site 10a (Wnt10a), mRNA.	NM_009518.1	3.91059	
GSTO1	Mus musculus glutathione S-transferase omega 1 (Gsto1), mRNA.	NM_010362.2	3.84759	
LOC217066	Mus musculus predicted gene, OTTMUSG00000025408 (OTTMUSG00000025408), non-coding RNA.	NR_003564.1	3.83718	
TINAGL	Mus musculus tubulointerstitial nephritis antigen-like (Tinagl), mRNA.	NM_023476.1	3.81307	
ANKRD1	Mus musculus ankyrin repeat domain 1 (cardiac muscle) (Ankrd1), mRNA.	NM_013468.2	3.79705	
STMN2	Mus musculus stathmin-like 2 (Stmn2), mRNA.	NM_025285.2	3.76379	*
SNRPN	Mus musculus SNRPN upstream reading frame (Snurf), mRNA.	NM_033174.2	3.73681	*
RHOD	Mus musculus ras homolog gene family, member D (Rhod), mRNA.	NM_007485.2	3.72967	
HSPB1	Mus musculus heat shock protein 1 (Hspb1), mRNA.	NM_013560.1	3.68691	*
SLITRK5	Mus musculus SLIT and NTRK-like family, member 5 (Slitrk5), mRNA.	NM_198865.1	3.6852	
A630005A06 RIK	Mus musculus TBC1 domain family, member 2 (Tbc1d2), mRNA.	NM_198664.3	3.51804	*
3110050N22 RIK	Mus musculus family with sequence similarity 164, member A (Fam164a), mRNA.	NM_173181.3	3.51754	
DOS	Mus musculus downstream of Stk11 (Dos), mRNA.	NM_015761.2	3.47437	*
LTBP1	Mus musculus latent transforming growth factor beta binding protein 1 (Ltbp1), transcript variant 2, mRNA.	NM_206958.1	3.46436	
TNFRSF12A	Mus musculus tumor necrosis factor receptor superfamily, member 12a (Tnfrsf12a), mRNA.	NM_013749.1	3.44943	*
AQP1	Mus musculus aquaporin 1 (Aqp1), mRNA.	NM_007472.1<br>NM_007472.1<br>NM_007472.2	3.36438<br>3.17094<br>2.68865	
FLRT2	Mus musculus fibronectin leucine rich transmembrane protein 2 (Flrt2), mRNA.	NM_201518.1	3.35539	
CHST7	Mus musculus carbohydrate (N-acetylglucosamino) sulfotransferase 7 (Chst7), mRNA.	NM_021715.1	3.30149	
SLC6A9	Mus musculus solute carrier family 6 (neurotransmitter transporter, glycine), member 9 (Slc6a9), mRNA.	NM_008135.4	3.29421	
CTGF	Mus musculus connective tissue growth factor (Ctgf), mRNA.	NM_010217.1	3.29075	*
HIST1H4J	Mus musculus histone cluster 1, H4j (Hist1h4j), mRNA.	NM_178210.1	3.27048	
HOXA7	Mus musculus homeo box A7 (Hoxa7), mRNA.	NM_010455.2	3.26892	
NUPR1	Mus musculus nuclear protein 1 (Nupr1), mRNA.	NM_019738.1	3.26419	
EPHA3	Mus musculus Eph receptor A3 (Epha3), mRNA.	NM_010140.1<br>NM_010140.1	3.25155<br>2.30077	
SCMH1	Mus musculus sex comb on midleg homolog 1 (Scmh1), mRNA.	NM_013883.1	3.21681	
SNCG	Mus musculus synuclein, gamma (Sncg), mRNA.	NM_011430.2	3.17605	
RIN1	Mus musculus Ras and Rab interactor 1 (Rin1), mRNA.	NM_145495.2	3.16499	
ATF5	Mus musculus activating transcription factor 5 (Atf5), mRNA.	NM_030693.1	3.16417	
COL7A1	Mus musculus collagen, type VII, alpha 1 (Col7a1), mRNA.	NM_007738.3	3.12923	*
CCNG1	Mus musculus cyclin G1 (Ccng1), mRNA.	NM_009831.2<br>NM_009831.2<br>NM_009831.2	3.10906<br>2.3024<br>2.06394	*
PDE4DIP	Mus musculus phosphodiesterase 4D interacting protein (myomegalin) (Pde4dip), transcript variant 1, mRNA.	NM_001039376.1	3.09392	

MOD1	PREDICTED: Mus musculus similar to Mod1 protein, transcript variant 4 (LOC677317), mRNA.	XM_001004685.1	3.08651	
DPP7	Mus musculus dipeptidylpeptidase 7 (Dpp7), mRNA.	NM_031843.2	3.06014	
PFN2	Mus musculus profilin 2 (Pfn2), mRNA.	NM_019410.2<br>NM_019410.2	3.05198<br>2.75215	
CCND1	Mus musculus cyclin D1 (Ccnd1), mRNA.	NM_007631.2<br>NM_007631.1<br>NM_007631.1	3.04646<br>2.63953<br>2.55278	
PKIA	Mus musculus protein kinase inhibitor, alpha (Pkia), mRNA.	NM_008862.3	3.04401	
CD109	Mus musculus CD109 antigen (Cd109), mRNA.	NM_153098.2<br>NM_153098.2	3.02402<br>2.37338	
FBXO25	Mus musculus F-box protein 25 (Fbxo25), mRNA.	NM_025785.2	3.01954	
CKLFSF4	Mus musculus CKLF-like MARVEL transmembrane domain containing 4 (Cmtm4), mRNA.	NM_153582.4	3.01163	
RNU6	Mus musculus U6 small nuclear RNA (Rnu6), non-coding RNA.	NR_003027.1	2.9508	
ELMO2	Mus musculus engulfment and cell motility 2, ced-12 homolog (C. elegans) (Elmo2), transcript variant 3, mRNA.	NM_080287.2	2.94356	
IRX2	Mus musculus Iroquois related homeobox 2 (Drosophila) (Irx2), mRNA.	NM_010574.2	2.94042	
1810045K06 RIK	Mus musculus kelch-like 21 (Drosophila) (Klhl21), mRNA.	NM_001033352.3	2.93826	
ACOT7	Mus musculus acyl-CoA thioesterase 7 (Acot7), mRNA.	NM_133348.1<br>NM_133348.1<br>NM_133348.1<br>NM_133348.1	2.93403<br>2.57733<br>2.48516<br>2.43181	
PLA2G12A	Mus musculus phospholipase A2, group XIIA (Pla2g12a), transcript variant 1, mRNA.	NM_023196.2	2.928	
EPHX1	Mus musculus epoxide hydrolase 1, microsomal (Ephx1), mRNA.	NM_010145.2	2.90728	*
PTRH1	Mus musculus peptidyl-tRNA hydrolase 1 homolog (S. cerevisiae) (Ptrh1), mRNA.	NM_178595.3<br>NM_178595.2	2.88622<br>2.74032	
NPR2	Mus musculus natriuretic peptide receptor 2 (Npr2), mRNA.	NM_173788.3	2.87031	
MLLT11	Mus musculus myeloid/lymphoid or mixed-lineage leukemia (trithorax homolog, Drosophila); translocated to, 11 (Mllt11), mRNA.	NM_019914.3	2.86807	
MOXD1	Mus musculus monooxygenase, DBH-like 1 (Moxd1), mRNA.	NM_021509.3	2.84276	
GDF15	Mus musculus growth differentiation factor 15 (Gdf15), mRNA.	NM_011819.1	2.84186	*
HSP105	Mus musculus heat shock protein 105 (Hsp105) mRNA.	NM_013559.1	2.83632	
HIST1H2BM	Mus musculus histone cluster 1, H2bm (Hist1h2bm), mRNA.	NM_178200.1	2.82491	*
MDM2	Mus musculus transformed mouse 3T3 cell double minute 2 (Mdm2), mRNA.	NM_010786.2<br>NM_010786.3	2.80901<br>2.71183	*
CCDC3	Mus musculus coil-coilied domain containing 3 (Ccdc3) mRNA.	XM_129987.2	2.78141	
ASS1	Mus musculus argininosuccinate synthetase 1 (Ass1), mRNA.	NM_007494.3	2.78125	*
SQSTM1	Mus musculus sequestosome 1 (Sqstm1), mRNA.	NM_011018.2	2.77037	
HIST1H2BJ	Mus musculus histone cluster 1, H2bj (Hist1h2bj), mRNA.	NM_178198.1	2.7552	*
TESK1	Mus musculus testis specific protein kinase 1 (Tesk1), mRNA.	NM_011571.2	2.75272	
ALDH3A1	Mus musculus aldehyde dehydrogenase family 3, subfamily A1 (Aldh3a1), mRNA.	NM_007436.1	2.73391	*
TRIB2	Mus musculus Tribbles homolog 2 (Trib2) mRNA.	NM_144551.3	2.72893	
ARHGEF18	Mus musculus rho/rac guanine nucleotide exchange factor (GEF) 18 (Arhgef18), mRNA.	NM_133962.3	2.72592	
KCTD10	Mus musculus potassium channel tetramerisation domain containing 10 (Kctd10), mRNA.	NM_026145.3	2.72172	*
AI450948	Mus musculus AHNAK nucleoprotein 2 (Ahnak2), mRNA.	NM_001033476.1	2.71731	
FHL2	Mus musculus four and a half LIM domains 2 (Fhl2), mRNA.	NM_010212.3	2.70708	
ST3GAL3	Mus musculus ST3 beta-galactoside alpha-2,3-sialyltransferase 3 (St3gal3), mRNA.	NM_009176.2	2.70341	
HIST1H2BH	Mus musculus histone cluster 1, H2bh (Hist1h2bh), mRNA.	NM_178197.1	2.69357	*

STK38L	Mus musculus serine/threonine kinase 38 like (Stk38l), mRNA.	NM_172734.2	2.6934	
SDPR	Mus musculus serum deprivation response (Sdpr), mRNA.	NM_138741.1	2.68312	
PRELP	Mus musculus proline arginine-rich end leucine-rich repeat (Prelp), mRNA.	NM_054077.3	2.68243	*
LMCD1	Mus musculus LIM and cysteine-rich domains 1 (Lmcd1), mRNA.	NM_144799.1<br>NM_144799.1	2.67584<br>2.56154	
CATNAL1	Mus musculus catenin (cadherin associated protein), alpha-like 1 (Ctnnal1), mRNA.	NM_018761.2	2.67576	
AU040829	Mus musculus expressed sequence AU040829 (AU040829), transcript variant 2, mRNA.	NM_175003.3	2.67252	
GSTP1	Mus musculus glutathione S-transferase, pi 1 (Gstp1), mRNA.	NM_013541.1<br>NM_013541.1	2.67226<br>2.47776	*
HIST1H2BK	Mus musculus histone cluster 1, H2bk (Hist1h2bk), mRNA.	NM_175665.1	2.6699	*
PDGFA	Mus musculus platelet-derived growth factor, A polypeptide (Pdgfa), mRNA.		2.63164	*
BC022687	Mus musculus cDNA sequence BC022687 (BC022687), mRNA.	NM_145450.3	2.6233	
CHST1	Mus musculus carbohydrate (keratan sulfate Gal-6) sulfotransferase 1 (Chst1), mRNA.	NM_023850.1	2.61691	
PRKG2	Mus musculus protein kinase, cGMP-dependent, type II (Prkg2), mRNA.	NM_008926.3	2.61221	
IL7	Mus musculus interleukin 7 (Il7), mRNA.	NM_008371.4	2.60881	
EFNA5	Mus musculus ephrin A5 (Efna5), transcript variant 2, mRNA.	NM_010109.2	2.60209	
ASNS	Mus musculus asparagine synthetase (Asns), mRNA.	NM_012055.3<br>NM_012055.1	2.60203<br>2.34067	
LRP11	Mus musculus low density lipoprotein receptor-related protein 11 (Lrp11), mRNA.	NM_172784.2	2.59931	
E2F6	Mus musculus E2F transcription factor 6 (E2f6), mRNA.	NM_033270.1<br>NM_033270.1	2.59526<br>2.18826	
SULF1	Mus musculus sulfatase 1 (Sulf1), mRNA.	NM_172294.1	2.58389	
DDEF1	PREDICTED: Mus musculus similar to Development and differentiation enhancing (LOC100045359), mRNA.	XM_001474179.1	2.581	
AVPI1	Mus musculus arginine vasopressin-induced 1 (Avpi1), mRNA.	NM_027106.3	2.56632	
D10ERTD214E	Mus musculus CDGSH iron sulfur domain 1 (Cisd1), mRNA.	NM_134007.4	2.56526	
HIST1H2BF	Mus musculus histone cluster 1, H2bf (Hist1h2bf), mRNA.	NM_178195.1	2.55937	*
2010323F13RIK	Mus musculus GTP cyclohydrolase I feedback regulator (Gchfr), mRNA.	NM_177157.4	2.55781	
C85492	Mus musculus expressed sequence C85492 (C85492), mRNA.	NM_153540.3	2.55722	
GNB4	Mus musculus guanine nucleotide binding protein (G protein), beta 4 (Gnb4), mRNA.	NM_013531.3	2.55171	
ENPP5	Mus musculus ectonucleotide pyrophosphatase/phosphodiesterase 5 (Enpp5), mRNA.	NM_032003.1	2.53694	
IRS2	Mus musculus insulin receptor substrate 2 (Irs2), mRNA.	NM_001081212.1	2.53676	
HIST1H2BE	Mus musculus histone cluster 1, H2be (Hist1h2be), mRNA.	NM_178194.3	2.53288	
PDGFC	Mus musculus platelet-derived growth factor, C polypeptide (Pdgfc), mRNA.	NM_019971.2	2.51907	
DDC8	Mus musculus testis specific protein, Ddc8 (Ddc8), mRNA.	NM_021440.1	2.51858	
LIMK2	Mus musculus LIM motif-containing protein kinase 2 (Limk2), transcript variant 3, mRNA.	NM_001034030.1	2.5175	
AHRR	Mus musculus aryl-hydrocarbon receptor repressor (Ahrr), mRNA.	NM_009644.2	2.51242	*
PHGDH	Mus musculus 3-phosphoglycerate dehydrogenase (Phgdh), mRNA.	NM_016966.3	2.50713	
4933428A15RIK	Mus musculus microfibrillar-associated protein 3-like (Mfap3l), mRNA.	NM_027756.3	2.50027	
MEGF10	Mus musculus multiple EGF-like-domains 10 (Megf10), mRNA.	NM_001001979.1	2.49787	
BTEB1	Mus musculus Kruppel-like factor 9 (Klf9), mRNA.	NM_010638.4	2.49532	
DDIT3	Mus musculus DNA-damage inducible transcript 3 (Ddit3), mRNA.	NM_007837.2<br>NM_007837.2	2.49149<br>2.42554	

TMBIM1	Mus musculus transmembrane BAX inhibitor motif containing 1 (Tmbim1), mRNA.	NM_027154.3	2.47126	
CSPG4	Mus musculus chondroitin sulfate proteoglycan 4 (Cspg4), mRNA.	NM_139001.2	2.47001	
ANXA3	Mus musculus annexin A3 (Anxa3), mRNA.	NM_013470.1<br>NM_013470.1	2.46116<br>2.43753	
D2BWG0891E	Mus musculus dysbindin (dystrobrevin binding protein 1) domain containing 2 (Dbndd2), transcript variant 3, mRNA.	NM_001048228.1	2.45135	
COQ10B	Mus musculus coenzyme Q10 homolog B (S. cerevisiae) (Coq10b), transcript variant 1, mRNA.	NM_001039710.1<br>NM_001039710.1	2.45009<br>2.0111	
PRLR	Mus musculus prolactin receptor (Prlr), mRNA.	NM_011169.4	2.43943	
FOXG1	Mus musculus forkhead box G1 (Foxg1), mRNA.	NM_008241.1	2.43518	
SLC19A2	Mus musculus solute carrier family 19 (thiamine transporter), member 2 (Slc19a2), mRNA.	NM_054087.2	2.43174	
CIB2	Mus musculus calcium and integrin binding family member 2 (Cib2), mRNA.	NM_019686.3	2.42973	
HSPB8	Mus musculus heat shock protein 8 (Hspb8), mRNA.	NM_030704.1	2.42948	*
PDK4	Mus musculus pyruvate dehydrogenase kinase, isoenzyme 4 (Pdk4), mRNA.	NM_013743.2	2.42519	
CAR11	Mus musculus carbonic anhydrase 11 (Car11), mRNA.	NM_009800.2	2.42404	
SLC20A2	PREDICTED: Mus musculus hypothetical protein LOC100045882 (LOC100045882), mRNA.	XM_001475083.1	2.40661	
2310046G15RIK	Mus musculus protease, serine, 23 (Prss23), mRNA.	NM_029614.3	2.40484	
THBD	Mus musculus thrombomodulin (Thbd), mRNA.	NM_009378.2	2.39873	
HIST1H2BN	Mus musculus histone cluster 1, H2bn (Hist1h2bn), mRNA.	NM_178201.1	2.39622	
1600002K03RIK	Mus musculus RIKEN cDNA 1600002K03 gene (1600002K03Rik), mRNA.	NM_027207.1	2.39613	
SULT4A1	Mus musculus sulfotransferase family 4A, member 1 (Sult4a1), mRNA.	NM_013873.3	2.38797	
9030611O19RIK	Mus musculus family with sequence similarity 110, member C (Fam110c), mRNA.	NM_027828.2	2.37875	
NGFB	Mus musculus nerve growth factor, beta (Ngfb), mRNA.	NM_013609.1	2.37565	
HIST1H4I	Mus musculus histone cluster 1, H4i (Hist1h4i), mRNA.	NM_175656.2	2.37374	
HIST1H4K	Mus musculus histone cluster 1, H4k (Hist1h4k), mRNA.	NM_178211.1	2.3728	
SGK	Mus musculus serum/glucocorticoid regulated kinase 1 (Sgk1), mRNA.	NM_011361.1	2.36214	*
ANGPTL2	Mus musculus angiopoietin-like 2 (Angptl2), mRNA.	NM_011923.4	2.35841	
SLC9A3R2	Mus musculus solute carrier family 9 (sodium/hydrogen exchanger), member 3 regulator 2 (Slc9a3r2), transcript variant A, mRNA.	NM_023055.1	2.35753	
SAMD4	Mus musculus sterile alpha motif domain containing 4 (Samd4), transcript variant 1, mRNA.	NM_001037221.1	2.35603	
NRN1	Mus musculus neuritin 1 (Nrn1), mRNA.	NM_153529.1	2.35489	
HIST1H3E	Mus musculus histone cluster 1, H3e (Hist1h3e), mRNA.	NM_178205.1	2.34212	
DP1	Mus musculus receptor accessory protein 5 (Reep5), mRNA.	NM_007874.2	2.3364	
1700024K14RIK	Mus musculus CAP-GLY domain containing linker protein family, member 4 (Clip4), mRNA.	NM_030179.2	2.33421	
WHRN	Mus musculus whirlin (Whrn), transcript variant 3, mRNA.	NM_001008792.1	2.3278	*
LOR	Mus musculus loricrin (Lor), mRNA.	NM_008508.2	2.32305	
CD9	Mus musculus tetraspanning glycoprotein 9 (Cd9) mRNA	NM_007657.2	2.32166	
1700047I17RIK	Mus musculus RIKEN cDNA 1700047I17 gene 1 (1700047I17Rik1), mRNA.	NM_028527.1	2.31688	
HES1	Mus musculus hairy and enhancer of split 1 (Drosophila) (Hes1), mRNA.	NM_008235.2	2.31173	
SNX7	Mus musculus sorting nexin 7 (Snx7), mRNA.	NM_029655.2	2.31142	
CKLFSF4	Mus musculus CKLF-like MARVEL transmembrane domain containing 4 (Cmtm4), mRNA.	NM_153582.4	2.3065	
CBR2	Mus musculus carbonyl reductase 2 (Cbr2), mRNA.	NM_007621.1	2.30116	

HIST1H3D	Mus musculus histone cluster 1, H3d (Hist1h3d), mRNA.	NM_178204.1	2.29977	
EEF1A2	Mus musculus eukaryotic translation elongation factor 1 alpha 2 (Eef1a2), mRNA.	NM_007906.2	2.29317	*
D8ERTD594E	Mus musculus WW, C2 and coiled-coil domain containing 2 (Wwc2), mRNA.	NM_133791.4	2.29045	
C630013N10 RIK	Mus musculus kelch-like 26 (Drosophila) (Klhl26), transcript variant 1, mRNA.	NM_172052.1	2.28996	
DAB2IP	Mus musculus disabled homolog 2 (Drosophila) interacting protein (Dab2ip), mRNA.	NM_001001602.1	2.27987	
MRPL38	Mus musculus mitochondrial ribosomal protein L38 (Mrpl38), nuclear gene encoding mitochondrial protein, mRNA.	NM_024177.3	2.27595	
HIST1H4M	Mus musculus histone cluster 1, H4m (Hist1h4m), mRNA.	NM_175657.1	2.27071	
PBP2	Mus musculus phosphatidylethanolamine binding protein 2 (Pbp2), mRNA.	NM_029595.3	2.26699	
CDO1	Mus musculus cysteine dioxygenase 1, cytosolic (Cdo1), mRNA.	NM_033037.3	2.25818	
KCNK2	Mus musculus potassium channel, subfamily K, member 2 (Kcnk2), mRNA.	NM_010607.1	2.25607	
IDH3A	Mus musculus isocitrate dehydrogenase 3 (NAD+) alpha (Idh3a), nuclear gene encoding mitochondrial protein, mRNA.	NM_029573.2	2.24384	
PMM1	Mus musculus phosphomannomutase 1 (Pmm1), mRNA.	NM_013872.1<br>NM_013872.2	2.24368<br>2.0541	
SLCO3A1	Mus musculus solute carrier organic anion transporter family, member 3a1 (Slco3a1), transcript variant 1, mRNA.	NM_023908.2	2.24125	
B230104P22 RIK	Mus musculus NUAK family, SNF1-like kinase, 1 (Nuak1), mRNA.	NM_001004363.1	2.23653	
NGFB	Mus musculus nerve growth factor, beta (Ngfb), mRNA.	NM_013609.1	2.22787	
2610204M08 RIK	Mus musculus RIKEN cDNA 2610204M08 gene (2610204M08Rik), mRNA.	NM_198411.2	2.21893	
HOXC6	Mus musculus homeo box C6 (Hoxc6), mRNA.	NM_010465.2	2.21521	*
NBL1	Mus musculus neuroblastoma, suppression of tumorigenicity 1 (Nbl1), mRNA.	NM_008675.1	2.21447	*
CD151	Mus musculus transmembrane-4 protein (Cd151) mRNA	NM_009842.1	2.21371<br>2.16579<br>2.1459	
2300002D11 RIK	Mus musculus RIKEN cDNA 2300002D11 gene (2300002D11Rik), mRNA.	NM_001081156.2	2.21367	*
D5BWG0834 E	Mus musculus coiled-coil domain containing 92 (Ccdc92), mRNA.	NM_144819.2	2.20015	
GTSE1	Mus musculus G two S phase expressed protein 1 (Gtse1), mRNA.	NM_013882.1	2.19507	
HMGA1	Mus musculus high mobility group AT-hook 1 (Hmga1), transcript variant 1, mRNA.	NM_016660.2	2.19481	
RNF11	Mus musculus ring finger protein 11 (Rnf11), mRNA.	NM_013876.3<br>NM_013876.2	2.19088<br>2.13822	
GDF10	Mus musculus growth differentiation factor 10 (Gdf10), mRNA.	NM_145741.2	2.19078	
SERPINE2	Mus musculus serine (or cysteine) peptidase inhibitor, clade E, member 2 (Serpine2), mRNA.	NM_009255.2	2.18924	
TLCD1	Mus musculus TLC domain containing 1 (Tlcd1), mRNA.	NM_026708.1	2.18619	
1810059A23 RIK	Mus musculus MTERF domain containing 2 (Mterfd2), mRNA.	NM_178051.3	2.17992	
CYB5R3	Mus musculus cytochrome b5 reductase 3 (Cyb5r3), mRNA.	NM_029787.2	2.17454	
CDKN1A	Mus musculus cyclin-dependent kinase inhibitor 1A (P21) (Cdkn1a), mRNA.	NM_007669.2<br>NM_007669.2	2.17063<br>2.03977	
D930001I22R IK	Mus musculus RIKEN cDNA D930001I22 gene (D930001I22Rik), mRNA.	NM_173397.2	2.16768	
TTLL11	Mus musculus tubulin tyrosine ligase-like family, member 11 (Ttll11), mRNA.	NM_028921.1	2.16575	
THSD6	Mus musculus ADAMTS-like 5 (Adamtsl5), transcript variant 2, mRNA.	NM_025629.2	2.16529	
9130213B05 RIK	Mus musculus RIKEN cDNA 9130213B05 gene (9130213B05Rik), mRNA.	NM_145562.2	2.16525	
CLN2	Mus musculus tripeptidyl peptidase I (Tpp1), mRNA.	NM_009906.4	2.16468	
BC046404	Mus musculus cDNA sequence BC046404 (BC046404), mRNA.	NM_198861.1	2.16108	

EPN2	Mus musculus epsin 2 (Epn2), mRNA.	NM_010148.2	2.15494	
IGFBP2	Mus musculus insulin-like growth factor binding protein 2 (Igfbp2), mRNA.	NM_008342.2	2.14973	
BTBD11	Mus musculus BTB (POZ) domain containing 11 (Btbd11), transcript variant 2, mRNA.	NM_001017525.1	2.13589	
CD80	Mus musculus CD80 antigen (Cd80), mRNA.	NM_009855.2	2.13447	
SLC5A7	Mus musculus solute carrier family 5 (choline transporter), member 7 (Slc5a7), mRNA.	NM_022025.3	2.13317	
SPNB2	Mus musculus spectrin beta 2 (Spnb2), transcript variant 1, mRNA.	NM_175836.2	2.13016	
C85492	Mus musculus expressed sequence C85492 (C85492), mRNA.	NM_153540.3	2.1278	
CDKN2A	Mus musculus cyclin-dependent kinase inhibitor 2A (Cdkn2a), transcript variant 1, mRNA.	NM_009877.2	2.12587	
AKT3	Mus musculus thymoma viral proto-oncogene 3 (Akt3), mRNA.	NM_011785.2	2.12536	
MOD1	Mus musculus malic enzyme, supernatant (Mod1), mRNA.	NM_008615.1	2.12224	
OSBPL3	Mus musculus oxysterol binding protein-like 3 (Osbpl3), mRNA.	NM_027881.1	2.12214	
LRRC28	Mus musculus leucine rich repeat containing 28 (Lrrc28), mRNA.	NM_175124.4	2.12175	
RUSC2	Mus musculus RUN and SH3 domain containing 2 (Rusc2), transcript variant 1, mRNA.	NM_199057.2<br>NM_199057.2	2.12044<br>2.07967	
PPM1F	Mus musculus protein phosphatase 1F (PP2C domain containing) (Ppm1f), mRNA.	NM_176833.3	2.11857	
TWIST2	Mus musculus Twist family gene 2 (Twist2) mRNA	NM_007855.1	2.11724	
OLFM1	Mus musculus olfactomedin 1 (Olfm1), transcript variant 2, mRNA.	NM_001038612.1	2.11547	
RGS4	Mus musculus regulator of G-protein signaling 4 (Rgs4), mRNA.	NM_009062.3	2.11307	
BC046404	PREDICTED: Mus musculus similar to CDNA sequence BC046404 (LOC100045343), misc RNA.	XR_031575.1	2.11236	
POLK	Mus musculus polymerase (DNA directed), kappa (Polk), mRNA.	NM_012048.2	2.11149	
HIST1H2BC	Mus musculus histone cluster 1, H2bc (Hist1h2bc), mRNA.	NM_023422.3	2.11057	
SKP1A	Mus musculus S-phase kinase-associated protein 1A (Skp1a), mRNA.	NM_011543.3	2.10718	
C530043G21 RIK	Mus musculus family with sequence similarity 20, member B (Fam20b), mRNA.	NM_145413.4	2.10425	
CAV1	Mus musculus caveolin 1, caveolae protein (Cav1), mRNA.	NM_007616.3	2.1	
DOK1	Mus musculus docking protein 1 (Dok1), mRNA.	NM_010070.3<br>NM_010070.3	2.0981<br>2.00769	
ANXA1	Mus musculus annexin A1 (Anxa1), mRNA.	NM_010730.2	2.09796	
LRRFIP1	Mus musculus leucine rich repeat (in FLII) interacting protein 1 (Lrrfip1), mRNA.	NM_008515.1	2.09756	
3222401M22 RIK	Mus musculus raftlin family member 2 (Rftn2), mRNA. XM_920263 XM_986521 XM_986556 XM_986596 XM_986637 XM_986673	NM_028713.1	2.09728	
GATA2	Mus musculus GATA binding protein 2 (Gata2), mRNA.	NM_008090.4	2.09705	
USP20	Mus musculus ubiquitin specific peptidase 20 (Usp20), mRNA.	NM_028846.3	2.09632	
ITGB5	Mus musculus integrin beta 5 (Itgb5), mRNA.	NM_010580.1	2.09482	
0610031J06R IK	Mus musculus RIKEN cDNA 0610031J06 gene (0610031J06Rik), mRNA.	NM_020003.1	2.09432	
GRASP	Mus musculus general receptor for phosphoinositides-associated scaffold protein (Grasp) mRNA	NM_019518.2	2.09076	
TMEM119	Mus musculus transmembrane protein 119 (Tmem119), mRNA.	NM_146162.1	2.08626	
1810046J19R IK	Mus musculus RIKEN cDNA 1810046J19 gene (1810046J19Rik), mRNA.	NM_025559.2	2.08391	
ENO2	Mus musculus enolase 2, gamma neuronal (Eno2), mRNA.	NM_013509.2	2.07747	
GSS	Mus musculus glutathione synthetase (Gss), mRNA.	NM_008180.1	2.07467	
D330024H06 RIK	Mus musculus KN motif and ankyrin repeat domains 1 (Kank1), mRNA.	NM_181404.5	2.0705	
AXL	Mus musculus AXL receptor tyrosine kinase (Axl), mRNA.	NM_009465.3	2.06773	

PTGS1	Mus musculus prostaglandin-endoperoxide synthase 1 (Ptgs1), mRNA.	NM_008969.3	2.06488	
RFTN2	Mus musculus raftlin family member 2 (Rftn2), mRNA. XM_920263 XM_986521 XM_986556 XM_986596 XM_986637 XM_986673	NM_028713.1	2.06413	
0610006K04 RIK	Mus musculus nudix (nucleoside diphosphate linked moiety X)-type motif 22 (Nudt22), mRNA.	NM_026675.2	2.06218	
HYAL1	Mus musculus hyaluronoglucosaminidase 1 (Hyal1), mRNA.	NM_008317.4	2.05647	
TLE1	Mus musculus transducin-like enhancer of split 1, homolog of Drosophila E(spl) (Tle1), mRNA. XM_984202 XM_984240 XM_984277 XM_984316 XM_984359 XM_984397 XM_984426 XM_984456 XM_984492 XM_984530	NM_011599.3	2.05144	
HAGH	Mus musculus hydroxyacyl glutathione hydrolase (Hagh), mRNA.	NM_024284.1	2.04763	
D7ERTD156E	Mus musculus nodal modulator 1 (Nomo1), mRNA.	NM_153057.3	2.04593	
2900046G09 RIK	Mus musculus family with sequence similarity 131, member A (Fam131a), mRNA.	NM_133778.2	2.04532	
2610001E17 RIK	Mus musculus coiled-coil domain containing 80 (Ccdc80), mRNA.	NM_026439.2	2.04149	
HIST1H4F	Mus musculus histone cluster 1, H4f (Hist1h4f), mRNA.	NM_175655.1	2.04141	
0610007H07 RIK	Mus musculus transmembrane BAX inhibitor motif containing 4 (Tmbim4), mRNA.	NM_026617.3	2.03485	
MGST3	Mus musculus microsomal glutathione S-transferase 3 (Mgst3), mRNA.	NM_025569.1	2.02646	
6330505N24 RIK	Mus musculus RIKEN cDNA 6330505N24 gene (6330505N24Rik), mRNA.	NM_001033301.2	2.02597	
2310066E14 RIK	Mus musculus RIKEN cDNA 2310066E14 gene (2310066E14Rik), mRNA.	NM_001081241.2	2.02182	
RAB3B	Mus musculus member RAS oncogene family RIKEN-rab3b (Rab3b) mRNA.	NM_023537.4	2.02047	
DCI	Mus musculus dodecenoyl-Coenzyme A delta isomerase (3,2 trans-enoyl-Coenyme A isomerase) (Dci), nuclear gene encoding mitochondrial protein, mRNA.	NM_010023.3	2.01962	
0610031J06R IK	Mus musculus RIKEN cDNA 0610031J06 gene (0610031J06Rik), mRNA.	NM_020003.1	2.01776	
GSTA1	Mus musculus glutathione S-transferase, alpha 1 (Ya) (Gsta1), mRNA.	NM_008181.2	2.01709	
HBS1L	Mus musculus Hbs1-like (S. cerevisiae) (Hbs1l), transcript variant 1, mRNA.	NM_019702.2	2.01706	
TRP53INP2	Mus musculus transformation related protein 53 inducible nuclear protein 2 (Trp53inp2), mRNA.	NM_178111.3	2.01696	
S100A4	Mus musculus S100 calcium binding protein A4 (S100a4), mRNA.	NM_011311.1	2.01615	
1500003O03 RIK	PREDICTED: Mus musculus similar to EF-hand Ca2+ binding protein p22 (LOC100048622), mRNA.	XM_001480443.1	2.01556	
IDB1	Mus musculus inhibitor of DNA binding 1 (Id1), mRNA.	NM_010495.2	2.01443	
ASAH1	Mus musculus N-acylsphingosine amidohydrolase 1 (Asah1), mRNA.	NM_019734.1	2.01175	

S3 B Table: Genes (493) downregulated by *Pthrp* ablation (at 1g) (fold change< 0.5, p < 0.05)

Target ID	Definition (all probes : *Mus musculus*)	RefSeq ID	Fold change	Down regulated by 0g
LYZS	Mus musculus lysozyme 2 (Lyz2), mRNA.	NM_017372.3<br>NM_017372.2	0.00777<br>0.0279	
LAPTM5	Mus musculus lysosomal-associated protein transmembrane 5 (Laptm5), mRNA.	NM_010686.3	0.01087	
FCER1G	Mus musculus high affinity immunoglobulin epsilon receptor gamma subunit (Fcer1g) mRNA.	NM_010185.2	0.01636	*
LYZ	Mus musculus lysozyme (Lyz), mRNA.	NM_013590.2<br>NM_013590.3	0.02094<br>0.38513	*
C1QB	Mus musculus complement component 1, q subcomponent, beta polypeptide (C1qb), mRNA.	NM_009777.2	0.02453	*
IGF2	Mus musculus insulin-like growth factor 2 (Igf2), mRNA.	NM_010514.2	0.02611	*

C1QG	Mus musculus complement component 1, q subcomponent, C chain (C1qc), mRNA.	NM_007574.2	0.03164	*
ALOX5AP	Mus musculus arachidonate 5-lipoxygenase activating protein (Alox5ap), mRNA.	NM_009663.1	0.03171	*
CLECSF8	Mus musculus C-type lectin domain family 4, member d (Clec4d), mRNA.	NM_010819.3	0.03345	
MS4A6D	Mus musculus membrane-spanning 4-domains, subfamily A, member 6D (Ms4a6d), mRNA.	NM_026835.2	0.03588	*
H19	Mus musculus H19 fetal liver mRNA (H19), non-coding RNA.	NR_001592.1	0.03605	*
CCL4	Mus musculus chemokine (C-C motif) ligand 4 (Ccl4), mRNA.	NM_013652.2	0.03678	
CCL9	Mus musculus chemokine (C-C motif) ligand 9 (Ccl9), mRNA.	NM_011338.2	0.03902	
EMR1	Mus musculus EGF-like module containing, mucin-like, hormone receptor-like sequence 1 (Emr1), mRNA.	NM_010130.3<br>NM_010130.1<br>NM_010130.3	0.04523<br>0.1316<br>0.38638	*
PTN	Mus musculus pleiotrophin (Ptn), mRNA.	NM_008973.2	0.04714	*
MS4A7	Mus musculus membrane-spanning 4-domains, subfamily A, member 7 (Ms4a7), transcript variant 1, mRNA.	NM_027836.5	0.04969	
CD68	Mus musculus CD68 antigen (Cd68), mRNA.	NM_009853.1	0.05004	
TREM2	Mus musculus triggering receptor expressed on myeloid cells 2 (Trem2), mRNA.	NM_031254.2	0.05084	
DCN	Mus musculus decorin (Dcn), mRNA.	NM_007833.4<br>NM_007833.4	0.05486<br>0.06403	*
LPL	Mus musculus lipoprotein lipase (Lpl), mRNA.	NM_008509.2	0.05499	
HIST1H2AO	Mus musculus histone cluster 1, H2ao (Hist1h2ao), mRNA.	NM_178185.1	0.05714	*
LUM	Mus musculus lumican (Lum), mRNA.	NM_008524.1	0.05734	*
2310061N23 RIK	Mus musculus interferon, alpha-inducible protein 27 (Ifi27), mRNA.	NM_029803.1	0.05903	*
HIST1H2AK	Mus musculus histone cluster 1, H2ak (Hist1h2ak), mRNA.	NM_178183.1	0.05962	*
CORO1A	Mus musculus coronin, actin binding protein 1A (Coro1a), mRNA.	NM_009898.2<br>NM_009898.2<br>NM_009898.2<br>NM_009898.2	0.05965<br>0.08707<br>0.16968<br>0.38779	*
HIST1H2AD	Mus musculus histone cluster 1, H2ad (Hist1h2ad), mRNA.	NM_178188.3	0.06131	*
CXCL1	Mus musculus chemokine (C-X-C motif) ligand 1 (Cxcl1), mRNA.	NM_008176.1	0.06474	
HIST1H2AF	Mus musculus histone cluster 1, H2af (Hist1h2af), mRNA.	NM_175661.1	0.06739	*
PFC	Mus musculus complement factor properdin (Cfp), mRNA.	NM_008823.3	0.07203	*
CD52	Mus musculus CD52 antigen (Cd52), mRNA.	NM_013706.1	0.07493	*
CTSC	Mus musculus cathepsin C (Ctsc), mRNA.	NM_009982.2<br>NM_009982.2<br>NM_009982.3	0.0758<br>0.18634<br>0.33872	*
HIST1H2AH	Mus musculus histone cluster 1, H2ah (Hist1h2ah), mRNA.	NM_175659.1	0.07772	*
SERPINA3G	Mus musculus serine (or cysteine) peptidase inhibitor, clade A, member 3G (Serpina3g), mRNA.	NM_009251.1	0.07795	
MYO1F	Mus musculus myosin IF (Myo1f), mRNA.	NM_053214.1	0.07882	
TGFBI	Mus musculus transforming growth factor, beta induced (Tgfbi), mRNA.	NM_009369.1<br>NM_009369.3	0.07927<br>0.38979	*
CASP1	Mus musculus caspase 1 (Casp1), mRNA.	NM_009807.2	0.08006	
CENPA	Mus musculus centromere protein A (Cenpa), mRNA.	NM_007681.2	0.08088	*
WBSCR5	Mus musculus linker for activation of T cells family, member 2 (Lat2), transcript variant 2, mRNA.	NM_022964.3	0.08091	
MRC1	Mus musculus mannose receptor, C type 1 (Mrc1), mRNA.	NM_008625.1	0.0815	*
HEMP1	Mus musculus NCK associated protein 1 like (Nckap1l), mRNA.	NM_153505.4	0.08261	*
PRC1	Mus musculus protein regulator of cytokinesis 1 (Prc1), mRNA.	NM_145150.1	0.08587	
SLC11A1	Mus musculus solute carrier family 11 (proton-coupled divalent metal ion transporters), member 1 (Slc11a1), mRNA.	NM_013612.1<br>NM_013612.1<br>NM_013612.1<br>NM_013612.1	0.08799<br>0.10977<br>0.18128<br>0.33293	

E030006K04 RIK	Mus musculus centaurin, delta 3 (Centd3), mRNA.	NM_139206.1	0.08861	
HIST1H2AN	Mus musculus histone cluster 1, H2an (Hist1h2an), mRNA.	NM_178184.1	0.09457	*
SFRP2	Mus musculus secreted frizzled-related protein 2 (Sfrp2), mRNA.	NM_009144.1	0.09652	*
1500015O10 RIK	Mus musculus RIKEN cDNA 1500015O10 gene (1500015O10Rik), mRNA.	NM_024283.2	0.09756	*
MDK	Mus musculus midkine (Mdk), transcript variant 3, mRNA.	NM_0010123 36.1 NM_010784.4	0.10108 0.20629	
COTL1	Mus musculus coactosin-like 1 (Dictyostelium) (Cotl1), mRNA.	NM_028071.1	0.10111	
AOC3	Mus musculus amine oxidase, copper containing 3 (Aoc3), mRNA.	NM_009675.1	0.10201	*
RASSF4	Mus musculus Ras association (RalGDS/AF-6) domain family member 4 (Rassf4), mRNA.	NM_178045.3 NM_178045.3	0.10287 0.1215	*
LMO2	Mus musculus LIM domain only 2 (Lmo2), mRNA.	NM_008505.3	0.10409	
NCF4	Mus musculus neutrophil cytosolic factor 4 (Ncf4), mRNA.	NM_008677.1	0.10803	*
TYROBP	Mus musculus TYRO protein tyrosine kinase binding protein (Tyrobp), mRNA.	NM_011662.2	0.10834	
FOS	Mus musculus FBJ osteosarcoma oncogene (Fos), mRNA.	NM_010234.2	0.11169	
CD14	Mus musculus CD14 antigen (Cd14), mRNA.	NM_009841.3	0.11175	*
CD84	Mus musculus CD84 antigen (Cd84), mRNA.	NM_013489.1	0.11252	
IGF1	Mus musculus insulin-like growth factor 1 (Igf1), transcript variant 1, mRNA.	NM_010512.3 NM_184052.2	0.11285 0.17522	
PTX3	Mus musculus long pentraxin 3 (Ptx3) mRNA.	NM_008987.2 NM_008987.3	0.11368 0.14017	*
OASL2	Mus musculus 2'-5' oligoadenylate synthetase-like 2 (Oasl2), mRNA.	NM_011854.1	0.11594	*
KCNAB2	Mus musculus potassium voltage-gated channel, shaker-related subfamily, beta member 2 (Kcnab2), mRNA.	NM_010598.2	0.11799	*
SLC1A3	Mus musculus solute carrier family 1 (glial high affinity glutamate transporter), member 3 (Slc1a3), mRNA.	NM_148938.2	0.11818	*
ZRANB3	Mus musculus zinc finger, RAN-binding domain containing 3 (Zranb3), mRNA. XM_896419 XM_896436 XM_912948 XM_922552 XM_922558 XM_922565 XM_922581 XM_922587 XM_922596 XM_974893 XM_974926 XM_974968 XM_975006 XM_975040 XM_975084	NM_027678.2	0.12044	
NPY	Mus musculus neuropeptide Y (Npy), mRNA.	NM_023456.2	0.12159	
LSP1	PREDICTED: Mus musculus predicted gene, ENSMUSG00000043795 (ENSMUSG00000043795), mRNA.	XM_00148083 5.1	0.12439	*
C3	Mus musculus complement 3 (C3) mRNA.	NM_009778.1	0.12541	*
SPON2	Mus musculus spondin 2, extracellular matrix protein (Spon2), mRNA.	NM_133903.2	0.12584	
PYGL	Mus musculus liver glycogen phosphorylase (Pygl), mRNA.	NM_133198.1 NM_133198.1	0.12663 0.35035	
EVI2A	Mus musculus ecotropic viral integration site 2a (Evi2a), transcript variant 2, mRNA.	NM_010161.3 NM_010161.3	0.12808 0.43273	
SRPX	Mus musculus sushi-repeat-containing protein (Srpx), mRNA.	NM_016911.4 NM_016911.4	0.12828 0.14382	*
E430025L02R IK	Mus musculus leucine rich repeat containing 33 (Lrrc33), mRNA.	NM_146069.4	0.1296	
LPXN	Mus musculus leupaxin (Lpxn), mRNA.	NM_134152.3	0.1316	
LILRB4	Mus musculus leukocyte immunoglobulin-like receptor, subfamily B, member 4 (Lilrb4), mRNA.	NM_013532.2	0.13167	
MMP12	Mus musculus matrix metallopeptidase 12 (Mmp12), mRNA.	NM_008605.3	0.13191	
ARL11	Mus musculus ADP-ribosylation factor-like 11 (Arl11), mRNA.	NM_177337.3	0.13262	
PSCDBP	Mus musculus cytohesin 1 interacting protein (Cytip), mRNA.	NM_139200.4	0.13288	*
SFRP1	Mus musculus secreted frizzled-related sequence protein 1 (Sfrp1) mRNA.	NM_013834.1	0.13323	*
DAB2	Mus musculus disabled homolog 2 (Drosophila) (Dab2), transcript variant 2, mRNA.	NM_0010087 02.1 NM_023118.1	0.13411 0.17926	*

ITM2A	Mus musculus integral membrane protein 2A (Itm2a), mRNA.	NM_008409.2	0.13895	*
UHRF1	Mus musculus ubiquitin-like, containing PHD and RING finger domains, 1 (Uhrf1), mRNA.	NM_010931.2	0.13962	
DPEP2	Mus musculus dipeptidase 2 (Dpep2), mRNA.	NM_176913.3	0.14003<br>0.18665	*
SCARA5	Mus musculus scavenger receptor class A, member 5 (putative) (Scara5), mRNA.	NM_028903.1	0.14055	*
DLK1	Mus musculus delta-like 1 homolog (Drosophila) (Dlk1), mRNA.	NM_010052.4	0.15137	*
PTPNS1	Mus musculus signal-regulatory protein alpha (Sirpa), mRNA.	NM_007547.2	0.15282	
MCM5	Mus musculus minichromosome maintenance deficient 5, cell division cycle 46 (S. cerevisiae) (Mcm5), mRNA.	NM_008566.2	0.15351	
FCGR3	Mus musculus Fc receptor, IgG, low affinity III (Fcgr3), mRNA.	NM_010188.4	0.15699	
EMB	Mus musculus embigin (Emb), mRNA.	NM_010330.3	0.15774	
MS4A6D	Mus musculus membrane-spanning 4-domains, subfamily A, member 6D (Ms4a6d), mRNA.	NM_026835.2	0.15781	
OLFML2B	Mus musculus olfactomedin-like 2B (Olfml2b), mRNA.	NM_177068.3	0.15971	
4930583H14 RIK	Mus musculus RIKEN cDNA 4930583H14 gene (4930583H14Rik), mRNA.	NM_026358.2	0.16845	*
TUBB2B	Mus musculus tubulin, beta 2b (Tubb2b), mRNA.	NM_023716.2<br>NM_023716.1	0.17209<br>0.44793	
P2RY6	Mus musculus pyrimidinergic receptor P2Y, G-protein coupled, 6 (P2ry6), mRNA.	NM_183168.1	0.17217	
USP18	Mus musculus ubiquitin specific peptidase 18 (Usp18), mRNA.<br>PREDICTED: Mus musculus similar to ubiquitin specific protease UBP43 (LOC100048346), mRNA.	NM_011909.1<br>M_001480051 .1	0.17303<br>0.25553	
TK1	Mus musculus thymidine kinase 1 (Tk1), mRNA.	NM_009387.1	0.17424	*
APOD	PREDICTED: Mus musculus similar to apolipoprotein D (LOC100047583), mRNA.	XM_00147913 8.1	0.17425	*
OAS1G	Mus musculus 2'-5' oligoadenylate synthetase 1G (Oas1g), mRNA.	NM_011852.2	0.1746	
POLR2G	Mus musculus polymerase (RNA) II (DNA directed) polypeptide G (Polr2g), mRNA.	NM_026329.2	0.17533	
VAV1	Mus musculus vav 1 oncogene (Vav1), mRNA.	NM_011691.3	0.17711	
PPP1R3C	Mus musculus protein phosphatase 1, regulatory (inhibitor) subunit 3C (Ppp1r3c), mRNA.	NM_016854.2	0.17777	*
CDCA3	Mus musculus cell division cycle associated 3 (Cdca3), mRNA.	NM_013538.4	0.1784	
FOLR2	Mus musculus folate receptor 2 (fetal) (Folr2), mRNA.	NM_008035.1	0.17841	
CXCL16	Mus musculus chemokine (C-X-C motif) ligand 16 (Cxcl16), mRNA.	NM_023158.6	0.17918	
ANLN	Mus musculus anillin, actin binding protein (Anln), mRNA.	NM_028390.2	0.17991	
FBLN1	Mus musculus fibulin 1 (Fbln1), mRNA.	NM_010180.1	0.18019	
CXCL4	Mus musculus chemokine (C-X-C motif) ligand 4 (Cxcl4), mRNA.	NM_019932.2	0.18065	*
MFAP2	Mus musculus microfibrillar-associated protein 2 (Mfap2), mRNA.	NM_008546.2<br>NM_008546.2<br>NM_008546.2	0.18086<br>0.259<br>0.27577	*
HP	Mus musculus haptoglobin (Hp), mRNA.	NM_017370.1<br>NM_017370.1<br>NM_017370.1	0.18333<br>0.41672<br>0.4727	*
NFIL3	PREDICTED: Mus musculus similar to NFIL3/E4BP4 transcription factor (LOC100046232), mRNA.	XM_00147581 7.1	0.18485	
CDC20	Mus musculus cell division cycle 20 homolog (S. cerevisiae) (Cdc20), mRNA.	NM_023223.1<br>NM_023223.1<br>NM_023223.1	0.18489<br>0.34963<br>0.35414	
PLK1	Mus musculus polo-like kinase 1 (Drosophila) (Plk1), mRNA.	NM_011121.3	0.18491	
HCPH	Mus musculus protein tyrosine phosphatase, non-receptor type 6 (Ptpn6), transcript variant 2, mRNA.	NM_0010777 05.1	0.18546	
LST1	Mus musculus liver-specific organic anion transporter (Lst1) mRNA.	XM_359281.1	0.18555	
TLR13	Mus musculus toll-like receptor 13 (Tlr13), mRNA.	NM_205820.1	0.1866	

C1QA	Mus musculus complement component 1, q subcomponent, alpha polypeptide (C1qa), mRNA.	NM_007572.2	0.19278	*
SLAMF9	Mus musculus SLAM family member 9 (Slamf9), mRNA.	NM_029612.3	0.19353	
APOBEC1	Mus musculus apolipoprotein B mRNA editing enzyme, catalytic polypeptide 1 (Apobec1), transcript variant 1, mRNA.	NM_031159.3	0.19424	
SMOC1	Mus musculus SPARC related modular calcium binding 1 (Smoc1), mRNA.	NM_022316.1	0.19476	
CCL3	Mus musculus chemokine (C-C motif) ligand 3 (Ccl3), mRNA.	NM_011337.2	0.19482	
EMILIN2	Mus musculus elastin microfibril interfacer 2 (Emilin2), mRNA.	NM_145158.2	0.19501	
PBK	Mus musculus PDZ binding kinase (Pbk), mRNA.	NM_023209.1	0.19636	
6330406I15RIK	Mus musculus RIKEN cDNA 6330406I15 gene (6330406I15Rik), mRNA.	NM_027519.1	0.19828	*
RPS6KA1	Mus musculus ribosomal protein S6 kinase polypeptide 1 (Rps6ka1), mRNA.	NM_009097.1	0.19858	
CYP7B1	Mus musculus cytochrome P450, family 7, subfamily b, polypeptide 1 (Cyp7b1), mRNA.	NM_007825.3	0.19979	*
BC013481	Mus musculus RNA binding motif protein 47 (Rbm47), mRNA.	NM_178446.2	0.20078	
FCRL3	Mus musculus Fc receptor, IgG, low affinity IV (Fcgr4), mRNA.	NM_144559.1	0.20276	
GMFG	Mus musculus glia maturation factor, gamma (Gmfg), transcript variant 1, mRNA.	NM_022024.2<br>NM_022024.2	0.20309<br>0.23556	*
BIRC5	Mus musculus baculoviral IAP repeat-containing 5 (Birc5), transcript variant 1, mRNA.	NM_009689.1<br>NM_009689.2<br>NM_009689.2	0.20396<br>0.24155<br>0.26431	
6330406I15RIK	Mus musculus RIKEN cDNA 6330406I15 gene (6330406I15Rik), mRNA.	NM_027519.3	0.20417	*
CCL4	Mus musculus chemokine (C-C motif) ligand 4 (Ccl4), mRNA.	NM_013652.2	0.20766	
1200002N14RIK	Mus musculus RIKEN cDNA 1200002N14 gene (1200002N14Rik), mRNA.	NM_027878.2	0.20795	*
APOB48R	Mus musculus apolipoprotein B48 receptor (Apob48r), mRNA.	NM_138310.1	0.20797	
CSF1R	Mus musculus colony stimulating factor 1 receptor (Csf1r), mRNA.	NM_001037859.2	0.2102	
IGFBP5	Mus musculus insulin-like growth factor binding protein 5 (Igfbp5), mRNA.	NM_010518.2	0.21306	*
1300002F13RIK	Mus musculus ERBB receptor feedback inhibitor 1 (Errfi1), mRNA.	NM_133753.1	0.2132	*
ELMO1	Mus musculus engulfment and cell motility 1, ced-12 homolog (C. elegans) (Elmo1), transcript variant 2, mRNA.	NM_198093.2	0.2135	
HMGN2	Mus musculus high mobility group nucleosomal binding domain 2 (Hmgn2), mRNA.	NM_016957.3	0.21351	
SLC15A3	Mus musculus solute carrier family 15, member 3 (Slc15a3), mRNA.	NM_023044.1	0.21475	
OLFML3	Mus musculus olfactomedin-like 3 (Olfml3), mRNA.	NM_133859.2	0.21513	
MYLK	Mus musculus myosin, light polypeptide kinase (Mylk), mRNA.	NM_139300.3<br>NM_139300.3	0.21625<br>0.2756	
ZFP36	Mus musculus zinc finger protein 36 (Zfp36), mRNA.	NM_011756.4	0.21637	
4632417K18RIK	Mus musculus RIKEN cDNA 4632417K18 gene (4632417K18Rik), mRNA.	NM_026640.2	0.21756	
COL3A1	Mus musculus collagen, type III, alpha 1 (Col3a1), mRNA.	NM_009930.1	0.2207	*
LOC100034251	Mus musculus predicted gene, OTTMUSG00000000971 (OTTMUSG00000000971), mRNA.	NM_001081957.1	0.22164	
COL18A1	Mus musculus procollagen, type XVIII, alpha 1 (Col18a1), mRNA.	NM_009929.2	0.2239	
RGS1	Mus musculus regulator of G-protein signaling 1 (Rgs1), mRNA.	NM_015811.1<br>NM_015811.1<br>NM_015811.1	0.22631<br>0.42553<br>0.45232	
APOE	Mus musculus apolipoprotein E (Apoe), mRNA.	NM_009696.2	0.22645	
NUSAP1	Mus musculus nucleolar and spindle associated protein 1 (Nusap1), transcript variant 2, mRNA.	NM_001042652.1	0.22666	
HK3	Mus musculus hexokinase 3 (Hk3), nuclear gene encoding mitochondrial protein, mRNA.	NM_001033245.3	0.22785	

ZFP36L1	Mus musculus zinc finger protein 36, C3H type-like 1 (Zfp36l1), mRNA.	NM_007564.2 NM_007564.4	0.22805 0.26544	*
LGMN	Mus musculus legumain (Lgmn), mRNA.	NM_011175.2	0.22864	*
H2-AB1	Mus musculus histocompatibility 2, class II antigen A, beta 1 (H2-Ab1), mRNA.	NM_207105.2	0.23107	
LGALS3BP	Mus musculus lectin, galactoside-binding, soluble, 3 binding protein (Lgals3bp), mRNA.	NM_011150.2	0.23114	
BTK	Mus musculus Bruton agammaglobulinemia tyrosine kinase (Btk), mRNA.	NM_013482.1	0.23157	
SOCS3	Mus musculus suppressor of cytokine signaling 3 (Socs3), mRNA.	NM_007707.2	0.23266	
FES	Mus musculus feline sarcoma oncogene (Fes), mRNA.	NM_010194.2	0.23406	
GBP2	Mus musculus guanylate binding protein 2 (Gbp2), mRNA.	NM_010260.1	0.23663	
CTSS	Mus musculus cathepsin S (Ctss), mRNA.	NM_021281.1	0.23739	
RBM3	PREDICTED: Mus musculus similar to RNA binding motif protein 3 (LOC100043257), mRNA.	XM_00148019 7.1	0.23785	*
RAB8B	Mus musculus RAB8B, member RAS oncogene family (Rab8b), mRNA.	NM_173413.2 NM_173413.2	0.23791 0.27441	
MYO1G	Mus musculus myosin IG (Myo1g), mRNA.	NM_178440.2	0.23811	
PARP14	Mus musculus poly (ADP-ribose) polymerase family, member 14 (Parp14), mRNA. XM_901644 XM_916789 XM_924484 XM_924488	NM_0010395 30.1	0.23892	
LCP1	Mus musculus lymphocyte cytosolic protein 1 (Lcp1), mRNA.	NM_008879.3	0.23989	
CDC2A	Mus musculus cell division cycle 2 homolog A (S. pombe) (Cdc2a), mRNA.	NM_007659.3	0.24236	
SLPI	Mus musculus secretory leukocyte peptidase inhibitor (Slpi), mRNA.	NM_011414.2	0.24292	*
UBE1L	Mus musculus ubiquitin-activating enzyme E1-like (Ube1l), mRNA.	NM_023738.4	0.24773	
SERPING1	Mus musculus serine (or cysteine) peptidase inhibitor, clade G, member 1 (Serping1), mRNA.	NM_009776.1	0.24935	*
DOK3	Mus musculus docking protein 3 (Dok3), mRNA.	NM_013739.2	0.24945	
A230050P20 RIK	Mus musculus RIKEN cDNA A230050P20 gene (A230050P20Rik), mRNA.	NM_175687.1	0.24993	
EDNRA	Mus musculus endothelin receptor type A (Ednra), mRNA.	NM_010332.2	0.25053	
SIRPB1	Mus musculus signal-regulatory protein beta 1 (Sirpb1), transcript variant 3, mRNA.	NM_0010028 98.1	0.25071	*
PFKL	Mus musculus phosphofructokinase, liver, B-type (Pfkl), mRNA.	NM_008826.3	0.25146	*
NRM	Mus musculus nurim (nuclear envelope membrane protein) (Nrm), mRNA.	NM_134122.2	0.25211	
ARRB2	Mus musculus arrestin, beta 2 (Arrb2), mRNA.	NM_145429.4	0.25506	
2310056P07 RIK	Mus musculus family with sequence similarity 162, member A (Fam162a), mRNA.	NM_027342.1	0.25677	*
AA175286	PREDICTED: Mus musculus sterile alpha motif domain containing 9-like, transcript variant 1 (Samd9l), mRNA.	XM_620286.3	0.25754	
GLRX	Mus musculus glutaredoxin (Glrx), mRNA.	NM_053108.2	0.25757	
ADAMTS2	Mus musculus a disintegrin-like and metallopeptidase (reprolysin type) with thrombospondin type 1 motif, 2 (Adamts2), mRNA.	NM_175643.2 NM_175643.2	0.25883 0.2796	
AI132321	Mus musculus phospholipase D family, member 4 (Pld4), mRNA.	NM_178911.4	0.2594	
STK6	Mus musculus aurora kinase A (Aurka), mRNA.	NM_011497.3	0.2609	
SELENBP1	PREDICTED: Mus musculus hypothetical protein LOC100044204 (LOC100044204), mRNA.	XM_00147169 6.1	0.26133	*
MFAP5	Mus musculus microfibrillar associated protein 5 (Mfap5), mRNA.	NM_015776.2	0.26267	
PIRA4	Mus musculus paired-Ig-like receptor A4 (Pira4), mRNA.	NM_011091.1	0.26305	*
HIST1H2AG	Mus musculus histone cluster 1, H2ag (Hist1h2ag), mRNA.	NM_178186.2	0.26455	*
ARHGAP30	Mus musculus Rho GTPase activating protein 30 (Arhgap30), mRNA.	NM_0010055 08.1	0.26709	
KNG1	Mus musculus kininogen 1 (Kng1), mRNA.	NM_023125.2	0.26822	
KIF22	Mus musculus kinesin family member 22 (Kif22), mRNA.	NM_145588.1	0.26835	

DOK2	Mus musculus docking protein 2 (Dok2), mRNA.	NM_010071.2	0.26926	
2700094K13 RIK	Mus musculus RIKEN cDNA 2700094K13 gene (2700094K13Rik), transcript variant 2, mRNA.	NM_0010372 79.1	0.27098	
PDGFB	Mus musculus platelet-derived growth factor B (Pdgfb) mRNA.	NM_011057.2	0.27199	
CD53	Mus musculus CD53 antigen (Cd53), mRNA.	NM_007651.2	0.27232	
AI586015	Mus musculus signal transducing adaptor family member 1 (Stap1), mRNA.	NM_019992.3	0.27272	
TGM2	Mus musculus transglutaminase 2, C polypeptide (Tgm2), mRNA.	NM_009373.3	0.2746	
IFIT3	Mus musculus interferon-induced protein with tetratricopeptide repeats 3 (Ifit3), mRNA.	NM_010501.1	0.27671	
GAS6	Mus musculus growth arrest specific 6 (Gas6), mRNA.	NM_019521.2	0.27858	
G1P2	PREDICTED: Mus musculus hypothetical protein LOC100038882 (LOC100038882), mRNA.	XM_00147168 6.1	0.27871	
AI481100	Mus musculus interferon inducible GTPase 2 (Iigp2), mRNA.	NM_019440.2	0.27876	
TGFBR3	Mus musculus transforming growth factor, beta receptor III (Tgfbr3), mRNA.	NM_011578.2	0.27966	
ADAM8	Mus musculus a disintegrin and metallopeptidase domain 8 (Adam8), mRNA.	NM_007403.2<br>NM_007403.2	0.27974<br>0.32845	
FYB	Mus musculus FYN binding protein (Fyb), mRNA.	NM_011815.1	0.2812	
CXCL12	Mus musculus chemokine (C-X-C motif) ligand 12 (Cxcl12), transcript variant 3, mRNA.	NM_013655.2<br>NM_021704.2<br>NM_021704.2	0.28324<br>0.37782<br>0.40775	*
TYMS	Mus musculus thymidylate synthase (Tyms), mRNA.	NM_021288.3	0.28611	
4732429D16 RIK	Mus musculus RIKEN cDNA 4732429D16 gene (4732429D16Rik), mRNA.	NM_145437.2	0.28756	
RAMP2	Mus musculus receptor (calcitonin) activity modifying protein 2 (Ramp2), mRNA.	NM_019444.2	0.28785	*
RSPO3	Mus musculus R-spondin 3 homolog (Xenopus laevis) (Rspo3), mRNA.	NM_028351.2	0.28962	*
TOP2A	Mus musculus topoisomerase (DNA) II alpha (Top2a), mRNA.	NM_011623.1	0.29004	
CXCL5	PREDICTED: Mus musculus similar to LPS-induced CXC chemokine (LOC100044702), mRNA.	XM_00147269 9.1	0.29091	
IFITM3	Mus musculus interferon induced transmembrane protein 3 (Ifitm3), mRNA.	NM_025378.2	0.29163	
AI467606	Mus musculus expressed sequence AI467606 (AI467606), mRNA.	NM_178901.3	0.29215	
2510004L01 RIK	Mus musculus radical S-adenosyl methionine domain containing 2 (Rsad2), mRNA.	NM_021384.3	0.29317	
FSTL1	Mus musculus follistatin-like 1 (Fstl1), mRNA.	NM_008047.4	0.29322	
LHFPL2	Mus musculus lipoma HMGIC fusion partner-like 2 (Lhfpl2), mRNA.	NM_172589.2	0.29358	
MEOX2	Mus musculus mesenchyme homeobox 2 (Meox2), mRNA.	NM_008584.3	0.29404	
D930038M1 3RIK	Mus musculus ABI gene family, member 3 (NESH) binding protein (Abi3bp), transcript variant 1, mRNA.	NM_178790.3	0.2955	*
LDB2	Mus musculus LIM domain-binding protein (Ldb2) mRNA.	NM_010698.2	0.29632	*
H2-M3	Mus musculus histocompatibility 2, M region locus 3 (H2-M3), mRNA.	NM_013819.2	0.29638	
DPT	Mus musculus dermatopontin (Dpt), mRNA.	NM_019759.2	0.29739	*
KCNK13	Mus musculus potassium channel, subfamily K, member 13 (Kcnk13), mRNA.	NM_146037.1	0.29916	
SLC2A1	Mus musculus solute carrier family 2 (facilitated glucose transporter), member 1 (Slc2a1), mRNA.	NM_011400.2	0.29917	*
IGSF4A	Mus musculus immunoglobulin superfamily member 4A (Igsf4a) mRNA.		0.30045	
LIG1	Mus musculus ligase I, DNA, ATP-dependent (Lig1), transcript variant 2, mRNA.	NM_010715.2	0.30209	
RNF144	Mus musculus ring finger protein 144A (Rnf144a), transcript variant 2, mRNA.	NM_080563.3	0.30219	
MCM6	Mus musculus minichromosome maintenance deficient 6 (MIS5 homolog, S. pombe) (S. cerevisiae) (Mcm6), mRNA.	NM_008567.1<br>NM_008567.1<br>NM_008567.1	0.30253<br>0.32313<br>0.39003	
PLEKHA2	Mus musculus pleckstrin homology domain-containing, family A (phosphoinositide binding specific) member 2 (Plekha2), mRNA.	NM_031257.2	0.30295	

OLFML1	Mus musculus olfactomedin-like 1 (Olfml1), mRNA.	NM_172907.2	0.30502	*
ADD3	Mus musculus adducin 3 (gamma) (Add3), mRNA.	NM_013758.2<br>NM_013758.2	0.30683<br>0.48848	*
CRABP1	Mus musculus cellular retinoic acid binding protein I (Crabp1), mRNA.	NM_013496.2	0.30772	
PRKCB	Mus musculus protein kinase C, beta (Prkcb), mRNA.	NM_008855.2	0.30783	
HIST2H2AC	Mus musculus histone cluster 2, H2ac (Hist2h2ac), mRNA.	NM_175662.1	0.30805	
NCAPH	Mus musculus non-SMC condensin I complex, subunit H (Ncaph), mRNA.	NM_144818.1	0.30911	
ETS2	Mus musculus E26 avian leukemia oncogene 2, 3' domain (Ets2), mRNA.	NM_011809.2	0.30972	
CUGBP2	Mus musculus CUG triplet repeat, RNA binding protein 2 (Cugbp2), transcript variant 6, mRNA.	NM_010160.2	0.31011	
EGLN3	Mus musculus EGL nine homolog 3 (C. elegans) (Egln3), mRNA.	NM_028133.1	0.31063	*
1200009O22 RIK	Mus musculus RIKEN cDNA 1200009O22 gene (1200009O22Rik), mRNA.	NM_025817.3	0.3116	*
HTR2B	Mus musculus 5-hydroxytryptamine (serotonin) receptor 2B (Htr2b), mRNA.	NM_008311.2	0.31208	
2810417H13 RIK	Mus musculus RIKEN cDNA 2810417H13 gene (2810417H13Rik), mRNA.	NM_026515.2	0.31595	
PLCG2	Mus musculus phospholipase C, gamma 2 (Plcg2), mRNA.	NM_172285.1	0.3161	
MCM4	Mus musculus minichromosome maintenance deficient 4 homolog (S. cerevisiae) (Mcm4), mRNA.	NM_008565.2	0.31697	
CCR5	Mus musculus chemokine (C-C motif) receptor 5 (Ccr5), mRNA.	NM_009917.2<br>NM_009917.2<br>NM_009917.4	0.31747<br>0.32485<br>0.38806	*
SMARCA1	Mus musculus SWI/SNF related, matrix associated, actin dependent regulator of chromatin, subfamily a, member 1 (Smarca1), mRNA.	NM_053123.3	0.31761	*
ALDH3B1	Mus musculus aldehyde dehydrogenase 3 family, member B1 (Aldh3b1), mRNA.	NM_026316.2<br>NM_026316.2	0.31888<br>0.47899	*
MEST	Mus musculus mesoderm specific transcript (Mest), mRNA.	NM_008590.1<br>NM_008590.1	0.31959<br>0.39186	*
TRF	Mus musculus transferrin (Trf), mRNA.	NM_133977.2	0.31982	
ANGPTL4	Mus musculus angiopoietin-like 4 (Angptl4), mRNA.	NM_020581.1	0.32088	
UNC93B1	Mus musculus unc-93 homolog B1 (C. elegans) (Unc93b1), mRNA.	NM_019449.1	0.32173	
AADACL1	Mus musculus arylacetamide deacetylase-like 1 (Aadacl1), mRNA.	NM_178772.2	0.32218	
EBI3	Mus musculus Epstein-Barr virus induced gene 3 (Ebi3), mRNA.	NM_015766.2	0.32241	
SH3BP2	Mus musculus SH3-domain binding protein 2 (Sh3bp2), mRNA.	NM_011893.2	0.32393	
LMNB1	Mus musculus lamin B1 (Lmnb1), mRNA.	NM_010721.1	0.3261	
SLC40A1	Mus musculus solute carrier family 40 (iron-regulated transporter), member 1 (Slc40a1), mRNA.	NM_016917.2	0.32805	
WAS	Mus musculus Wiskott-Aldrich syndrome homolog (human) (Was), mRNA.	NM_009515.1	0.32929	
HTRA3	Mus musculus HtrA serine peptidase 3 (Htra3) mRNA.	NM_030127.1	0.32964	
XDH	Mus musculus xanthine dehydrogenase (Xdh), mRNA.	NM_011723.2	0.33023	
SELPL	Mus musculus selectin, platelet (p-selectin) ligand (Selplg), mRNA.	NM_009151.2	0.33025	
NID2	Mus musculus nidogen 2 (Nid2), mRNA.	NM_008695.2	0.3322	*
EHD4	Mus musculus EH-domain containing 4 (Ehd4), mRNA.	NM_133838.4	0.33274	*
OAS2	Mus musculus 2'-5' oligoadenylate synthetase 2 (Oas2), mRNA.	NM_145227.1	0.33594	
FMO1	Mus musculus flavin containing monooxygenase 1 (Fmo1), mRNA.	NM_010231.2	0.33665	*
ARHGAP4	Mus musculus Rho GTPase activating protein 4 (Arhgap4), mRNA.	NM_138630.1	0.33721	
PVRL2	Mus musculus poliovirus receptor-related 2 (Pvrl2), mRNA.	NM_008990.2<br>NM_008990.2	0.33784<br>0.41621	
KLF2	Mus musculus Kruppel-like factor 2 (lung) (Klf2), mRNA.	NM_008452.1	0.33799	
MCM10	Mus musculus minichromosome maintenance deficient 10 (S. cerevisiae) (Mcm10), mRNA.	NM_027290.1	0.34008	

PIK3CG	Mus musculus phosphoinositide-3-kinase, catalytic, gamma polypeptide (Pik3cg), mRNA.	NM_020272.1	0.34035	
PSTPIP1	Mus musculus proline-serine-threonine phosphatase-interacting protein 1 (Pstpip1), mRNA.	NM_011193.1	0.34421	
STARD8	Mus musculus START domain containing 8 (Stard8), mRNA.	NM_199018.1<br>NM_199018.1	0.34453<br>0.44115	
COL1A1	Mus musculus collagen, type I, alpha 1 (Col1a1), mRNA.	NM_007742.2	0.34461	
FEN1	Mus musculus flap structure specific endonuclease 1 (Fen1), mRNA.	NM_007999.3	0.34486	
PRDX4	Mus musculus peroxiredoxin 4 (Prdx4), mRNA.	NM_016764.3	0.34493	
FGFR1OP2	Mus musculus FGFR1 oncogene partner 2 (Fgfr1op2), mRNA.	NM_026218.2	0.34529	
MMP13	Mus musculus matrix metallopeptidase 13 (Mmp13), mRNA.	NM_008607.1	0.34616	
HCLS1	Mus musculus hematopoietic cell specific Lyn substrate 1 (Hcls1), mRNA.	NM_008225.1	0.34628	
PSMB9	Mus musculus proteasome (prosome, macropain) subunit, beta type 9 (large multifunctional peptidase 2) (Psmb9), mRNA.	NM_013585.2	0.34632	
ACTG2	Mus musculus actin, gamma 2, smooth muscle, enteric (Actg2), mRNA.	NM_009610.1	0.34644	
CDKN1C	Mus musculus cyclin-dependent kinase inhibitor 1C (P57) (Cdkn1c), mRNA.	NM_009876.3	0.3476	*
AGTR1A	Mus musculus angiotensin II receptor, type 1a (Agtr1a), mRNA.	NM_177322.2	0.34774	*
1190002H23 RIK	Mus musculus RIKEN cDNA 1190002H23 gene (1190002H23Rik), mRNA.	NM_025427.2	0.34816	*
NNMT	Mus musculus nicotinamide N-methyltransferase (Nnmt), mRNA.	NM_010924.1	0.34839	
STXBP2	Mus musculus syntaxin-binding protein 2 (Stxbp2) mRNA.	NM_011503.2	0.34934	
PLEKHA4	Mus musculus pleckstrin homology domain containing, family A (phosphoinositide binding specific) member 4 (Plekha4), mRNA.	NM_148927.1	0.35053	
CD180	Mus musculus CD180 antigen (Cd180), mRNA.	NM_008533.2	0.35076	
D12ERTD553 E	Mus musculus DNA segment, Chr 12, ERATO Doi 553, expressed (D12Ertd553e), mRNA.	NM_029758.3	0.35205	
2310016C08 RIK	Mus musculus RIKEN cDNA 2310016C08 gene (2310016C08Rik), mRNA.	NM_023516.3	0.35281	
BC032204	Mus musculus fermitin family homolog 3 (Drosophila) (Fermt3), mRNA.	NM_153795.1	0.35284	
KIF2C	Mus musculus kinesin family member 2C (Kif2c), mRNA. XM_986361	NM_134471.3	0.35353	
SERPINA3N	Mus musculus serine (or cysteine) peptidase inhibitor, clade A, member 3N (Serpina3n), mRNA.	NM_009252.2	0.35442	
TYMS-PS	Mus musculus thymidylate synthase, pseudogene (Tyms-ps), non-coding RNA.	NR_000040.1	0.35575	
CDCA7	Mus musculus cell division cycle associated 7 (Cdca7), mRNA.	NM_025866.3	0.35656	
TXNIP	Mus musculus thioredoxin interacting protein (Txnip), transcript variant 1, mRNA.	NM_0010099 35.2	0.35698	*
2310057H16 RIK	Mus musculus tubulin, beta 6 (Tubb6), mRNA.	NM_026473.2	0.35893	
BICC1	Mus musculus bicaudal C homolog 1 (Drosophila) (Bicc1), mRNA.	NM_031397.2	0.35937	
LBP	Mus musculus lipopolysaccharide binding protein (Lbp), mRNA.	NM_008489.2	0.35964	*
COL6A2	Mus musculus procollagen, type VI, alpha 2 (Col6a2), mRNA.	NM_146007.1	0.36	
GPNMB	Mus musculus glycoprotein (transmembrane) nmb (Gpnmb), mRNA.	NM_053110.3<br>NM_053110.2	0.36165<br>0.49491	
5430435G22 RIK	Mus musculus RIKEN cDNA 5430435G22 gene (5430435G22Rik), mRNA.	NM_145509.1	0.36174	
D14ERTD226 E	Mus musculus tetraspanin 14 (Tspan14), mRNA.	NM_145928.1	0.3619	
LY9	Mus musculus lymphocyte antigen 9 (Ly9), mRNA.	NM_008534.2	0.36195	
THBS2	Mus musculus thrombospondin 2 (Thbs2), mRNA.	NM_011581.1	0.36245	
RGS10	Mus musculus regulator of G-protein signalling 10 (Rgs10), mRNA.	NM_026418.2	0.36297	
H2-AB1	PREDICTED: Mus musculus similar to MHC class II antigen beta chain (LOC641240), mRNA.	XM_918601.3	0.36309	
FIGNL1	Mus musculus fidgetin-like 1 (Fignl1), mRNA.	NM_021891.2	0.3644	

1200013B08 RIK	Mus musculus RIKEN cDNA 1200013B08 gene (1200013B08Rik), mRNA.	NM_028773.2	0.36454	
SAMHD1	Mus musculus SAM domain and HD domain, 1 (Samhd1), mRNA.	NM_018851.2	0.36484	
CD248	Mus musculus CD248 antigen, endosialin (Cd248), mRNA.	NM_054042.2	0.36556	
PLAC8	Mus musculus placenta-specific 8 (Plac8), mRNA.	NM_139198.1	0.36585	
H2AFZ	Mus musculus H2A histone family, member Z (H2afz), mRNA.	NM_016750.1	0.36586	
AL024069	Mus musculus RIKEN cDNA 5033414K04 gene (5033414K04Rik), mRNA.	NM_0010039 48.1	0.36658	
PDK1	Mus musculus pyruvate dehydrogenase kinase, isoenzyme 1 (Pdk1), nuclear gene encoding mitochondrial protein, mRNA.	NM_172665.3	0.36676	*
AW046396	Mus musculus a disintegrin and metallopeptidase domain 23 (Adam23), mRNA.	NM_011780.2	0.36698	
RARRES2	Mus musculus retinoic acid receptor responder (tazarotene induced) 2 (Rarres2), mRNA.	NM_027852.1	0.36712	*
CX3CR1	Mus musculus chemokine CX3C receptor 1 (Cx3cr1) mRNA.	NM_009987.2	0.36835	
SPAG5	Mus musculus sperm associated antigen 5 (Spag5), mRNA.	NM_017407.1	0.36915	
HAS2	Mus musculus hyaluronan synthase 2 (Has2), mRNA.	NM_008216.2	0.37107	
PMP22	Mus musculus peripheral myelin protein 22 (Pmp22), mRNA.	NM_008885.2<br>NM_008885.2	0.3713<br>0.46766	
MSR2	Mus musculus macrophage scavenger receptor 2 (Msr2), mRNA.	NM_030707.1	0.37187	
KIF23	Mus musculus kinesin family member 23 (Kif23), mRNA.	NM_024245.3	0.37337	
RASL12	Mus musculus RAS-like, family 12 (Rasl12), mRNA.	NM_0010331 58.1	0.37355	
LIP1	Mus musculus lysosomal acid lipase 1 (Lip1) mRNA.	NM_021460.1	0.37369	
OAS1G	Mus musculus 2'-5' oligoadenylate synthetase 1G (Oas1g), mRNA.	NM_011852.2	0.37414	
SEPP1	Mus musculus selenoprotein P, plasma, 1 (Sepp1), transcript variant 1, mRNA.	NM_009155.3	0.3748	
SAP30	Mus musculus sin3 associated polypeptide (Sap30), mRNA.	NM_021788.1	0.37556	
IGFBP3	Mus musculus insulin-like growth factor binding protein 3 (Igfbp3), mRNA.	NM_008343.2	0.37679	*
CPXM1	Mus musculus carboxypeptidase X 1 (M14 family) (Cpxm1), mRNA.	NM_019696.1	0.37692	
1810009M01 RIK	Mus musculus transmembrane protein 176B (Tmem176b), mRNA.	NM_023056.3	0.37818	
LY6C	Mus musculus lymphocyte antigen 6 complex, locus C1 (Ly6c1), mRNA.	NM_010741.2	0.37845	
SESN1	Mus musculus sestrin 1 (Sesn1), mRNA.	NM_0010133 70.1	0.37882	
6330403K07 RIK	Mus musculus RIKEN cDNA 6330403K07 gene (6330403K07Rik), mRNA.	NM_134022.2	0.37961	
SOCS2	Mus musculus suppressor of cytokine signaling 2 (Socs2), mRNA.	NM_007706.3	0.38076	
CAR9	Mus musculus carbonic anhydrase 9 (Car9), mRNA.	NM_139305.1	0.38079	*
MMP23	Mus musculus matrix metallopeptidase 23 (Mmp23), mRNA.	NM_011985.2	0.38102	
H2-AB1	Mus musculus histocompatibility 2, class II antigen A, beta 1 (H2-Ab1), mRNA.	NM_207105.2	0.38152	
WBSCR5	Mus musculus linker for activation of T cells family, member 2 (Lat2), transcript variant 2, mRNA.	NM_022964.3	0.38344	
TBL2	Mus musculus transducin 2 (Tbl2) mRNA.		0.38379	
E130203B14 RIK	Mus musculus RIKEN cDNA E130203B14 gene (E130203B14Rik), mRNA.	NM_178791.4	0.38453	
NDRG1	Mus musculus N-myc downstream regulated gene 1 (Ndrg1), mRNA.	NM_008681.2	0.38702	*
GALNT9	Mus musculus UDP-N-acetyl-alpha-D-galactosamine:polypeptide N-acetylgalactosaminyltransferase 9 (Galnt9), mRNA.	NM_198306.1	0.39064	*
IGTP	Mus musculus interferon gamma induced GTPase (Igtp), mRNA.	NM_018738.3	0.39073	
CDCA2	Mus musculus cell division cycle associated 2 (Cdca2), mRNA.	NM_175384.3	0.39254	
CCNB1	Mus musculus cyclin B1 (Ccnb1), mRNA.	NM_172301.3<br>NM_172301.3	0.39272<br>0.4558	

SLA	Mus musculus src-like adaptor (Sla), transcript variant 2, mRNA.	NM_009192.2	0.39273	
FBN1	Mou musculus fibrillin-1 (Fbn1) mRNA		0.39273	
PLEK	Mus musculus pleckstrin (Plek), mRNA.	NM_019549.1	0.39531	
IL13RA1	Mus musculus interleukin 13 receptor, alpha 1 (Il13ra1), mRNA.	NM_133990.4	0.39548	
PDGFRA	Mus musculus platelet derived growth factor receptor, alpha polypeptide (Pdgfra), transcript variant 1, mRNA.	NM_011058.2	0.39649	*
CASP2	Mus musculus caspase 2 (Casp2), mRNA.	NM_007610.1	0.39863	
TNFAIP8	Mus musculus tumor necrosis factor, alpha-induced protein 8 (Tnfaip8), mRNA.	NM_134131.1	0.39963	
ISGF3G	Mus musculus interferon regulatory factor 9 (Irf9), mRNA.	NM_008394.2	0.40111	
RRM1	Mus musculus ribonucleotide reductase M1 (Rrm1), mRNA.	NM_009103.2	0.40131	
BC022145	Mus musculus transmembrane protein 106A (Tmem106a), mRNA.	NM_144830.3	0.4018	
SYNGR1	Mus musculus synaptogyrin 1 (Syngr1), transcript variant 1b, mRNA.	NM_009303.2	0.40244	
CH25H	Mus musculus cholesterol 25-hydroxylase (Ch25h), mRNA.	NM_009890.1	0.40267	
PIRA3	Mus musculus paired-Ig-like receptor A3 (Pira3), mRNA.	NM_011090.1	0.40279	
ZFHX1B	Mus musculus zinc finger E-box binding homeobox 2 (Zeb2), transcript variant 2, mRNA.	NM_015753.3	0.40296	
JUNB	Mus musculus Jun-B oncogene (Junb), mRNA.	NM_008416.1	0.40376	
GRN	Mus musculus granulin (Grn), mRNA.	NM_008175.3	0.4046	
NDRL			0.40565	*
HAT1	Mus musculus histone aminotransferase 1 (Hat1), mRNA.	NM_026115.3	0.40591	
ZDHHC14	Mus musculus zinc finger, DHHC domain containing 14 (Zdhhc14), mRNA.	NM_146073.3	0.40697	
PTPN18	Mus musculus protein tyrosine phosphatase, non-receptor type 18 (Ptpn18), mRNA.	NM_011206.2	0.40708	
ATP2A3	Mus musculus ATPase, Ca++ transporting, ubiquitous (Atp2a3), mRNA.	NM_016745.2	0.4074	
TMSB10	Mus musculus thymosin, beta 10 (Tmsb10), mRNA.	NM_025284.3<br>NM_025284.3	0.40747<br>0.49716	
GSTT1	Mus musculus glutathione S-transferase, theta 1 (Gstt1), mRNA.	NM_008185.2	0.40799	
GP49A	Mus musculus glycoprotein 49 A (Gp49a), mRNA.	NM_008147.1	0.40905	
ADFP	Mus musculus adipose differentiation related protein (Adfp), mRNA.	NM_007408.3	0.40936	
C1QR1	Mus musculus CD93 antigen (Cd93), mRNA.	NM_010740.3	0.40998	
ARHGAP20	Mus musculus Rho GTPase activating protein 20 (Arhgap20), mRNA.	NM_175535.3	0.41029	*
CHAF1B	Mus musculus chromatin assembly factor 1, subunit B (p60) (Chaf1b), mRNA.	NM_028083.3	0.41039	
6330406L22 RIK	Mus musculus histocompatibility (minor) HA-1 (Hmha1), mRNA.	NM_027521.2	0.41153	
ACTB	Mus musculus actin, beta, cytoplasmic (Actb), mRNA.	NM_007393.1	0.41154	
E130016E03 RIK	Mus musculus RIKEN cDNA E130016E03 gene (E130016E03Rik), mRNA.	NM_0010395 56.2	0.41212	
CLSPN	Mus musculus claspin homolog (Xenopus laevis) (Clspn), mRNA.	NM_175554.3<br>NM_175554.3	0.41361<br>0.49682	
SAMSN1	Mus musculus SAM domain, SH3 domain and nuclear localization signals, 1 (Samsn1), mRNA.	NM_023380.2	0.41591	
KLHL6	Mus musculus kelch-like 6 (Drosophila) (Klhl6), mRNA.	NM_183390.1<br>NM_183390.1	0.4164<br>0.48716	
ARHGAP18	Mus musculus Rho GTPase activating protein 18 (Arhgap18), mRNA.	NM_176837.2	0.41644	
GBP4	Mus musculus guanylate binding protein 3 (Gbp3), mRNA.	NM_018734.3<br>NM_018734.2	0.41726<br>0.44809	
MAD2L1	Mus musculus MAD2 (mitotic arrest deficient, homolog)-like 1 (yeast) (Mad2l1), mRNA.	NM_019499.2	0.41746	
6430706D22 RIK	Mus musculus RIKEN cDNA 6430706D22 gene (6430706D22Rik), mRNA.	NM_198652.1	0.41791	
PTPRO	Mus musculus protein tyrosine phosphatase, receptor type, O (Ptpro), mRNA.	NM_011216.2	0.41865	

ARRDC4	Mus musculus arrestin domain containing 4 (Arrdc4), transcript variant 1, mRNA.	NM_001042592.2	0.41884	
H2-GS17	Mus musculus predicted gene, EG667977 (EG667977), mRNA.	NM_001081032.1	0.41928	
FCGR2B	Mus musculus Fc receptor, IgG, low affinity IIb (Fcgr2b), transcript variant 2, mRNA.	NM_010187.2	0.42161	
ARHGDIB	Mus musculus Rho, GDP dissociation inhibitor (GDI) beta (Arhgdib), mRNA.	NM_007486.1	0.42174	
5430400N05RIK	Mus musculus zinc finger protein 710 (Zfp710), mRNA.	NM_175433.4	0.42278	
RIS2	Mus musculus chromatin licensing and DNA replication factor 1 (Cdt1), mRNA.	NM_026014.3	0.42286	
MBC2	Mus musculus membrane bound C2 domain containing protein (Mbc2), mRNA.	NM_011843.2	0.42419	
EGFR	Mus musculus epidermal growth factor receptor (Egfr), transcript variant 1, mRNA.	NM_207655.2	0.42472	
3632451O06RIK	Mus musculus RIKEN cDNA 3632451O06 gene (3632451O06Rik), mRNA.	NM_026142.1	0.42621	
SMPDL3B	Mus musculus sphingomyelin phosphodiesterase, acid-like 3B (Smpdl3b), mRNA.	NM_133888.2	0.42669	
PIRA11	Mus musculus paired-Ig-like receptor A11 (Pira11), mRNA.	NM_011088.1	0.42732	
H2-T23	Mus musculus histocompatibility 2, T region locus 23 (H2-T23), mRNA.	NM_010398.1	0.42843	
BC025872	Mus musculus RCSD domain containing 1 (Rcsd1), transcript variant 1, mRNA.	NM_178593.3	0.42847	
GPX7	Mus musculus glutathione peroxidase 7 (Gpx7), mRNA.	NM_024198.3	0.42906	
AW491445	Mus musculus solute carrier family 25, member 45 (Slc25a45), mRNA.	NM_134154.3	0.43047	
D12ERTD647E	Mus musculus DNA segment, Chr 12, ERATO Doi 647, expressed (D12Ertd647e), transcript variant 5, mRNA.	NM_194069.1	0.43143	
D12ERTD647E	Mus musculus DNA segment, Chr 12, ERATO Doi 647, expressed (D12Ertd647e), transcript variant 4, mRNA.	NM_194068.1	0.4316	
BC025206	Mus musculus UDP-GlcNAc:betaGal beta-1,3-N-acetylglucosaminyltransferase 8 (B3gnt8), transcript variant 1, mRNA.	NM_146184.4	0.43223	
NCAPD2	Mus musculus non-SMC condensin I complex, subunit D2 (Ncapd2), mRNA.	NM_146171.1	0.4325	
QPCT	Mus musculus glutaminyl-peptide cyclotransferase (glutaminyl cyclase) (Qpct), mRNA.	NM_027455.1	0.43283	
PRKR	Mus musculus eukaryotic translation initiation factor 2-alpha kinase 2 (Eif2ak2), mRNA.	NM_011163.3	0.43311	
PSD4	Mus musculus pleckstrin and Sec7 domain containing 4 (Psd4), mRNA.	NM_177611.3	0.43423	
PIK3R1	Mus musculus phosphatidylinositol 3-kinase, regulatory subunit, polypeptide 1 (p85 alpha) (Pik3r1), transcript variant 2, mRNA.	NM_001077495.1 NM_001024955.1	0.43484 0.4383	
CSRP2	Mus musculus cysteine and glycine-rich protein 2 (Csrp2), mRNA.	NM_007792.3	0.4354	
IRAK3	Mus musculus interleukin-1 receptor-associated kinase 3 (Irak3), mRNA.	NM_028679.3	0.43703	
YWHAH	Mus musculus tyrosine 3-monooxygenase/tryptophan 5-monooxygenase activation protein, eta polypeptide (Ywhah), mRNA.	NM_011738.1	0.43719	
2610039C10RIK	Mus musculus RIKEN cDNA 2610039C10 gene (2610039C10Rik), mRNA.	NM_025642.1	0.43726	
CRYL1	Mus musculus crystallin, lambda 1 (Cryl1), mRNA.	NM_030004.2	0.43785	
PAFAH1B3	Mus musculus platelet-activating factor acetylhydrolase, isoform 1b, alpha1 subunit (Pafah1b3), mRNA.	NM_008776.1	0.43863	
E2F2	Mus musculus E2F transcription factor 2 (E2f2), mRNA.	NM_177733.2	0.43876	
H2-AB1	Mus musculus histocompatibility 2, class II antigen A, beta 1 (H2-Ab1), mRNA.	NM_207105.1	0.43886	
BLVRB	Mus musculus biliverdin reductase B (flavin reductase (NADPH)) (Blvrb), mRNA.	NM_144923.2	0.44301	
UGT1A10	Mus musculus UDP glycosyltransferase 1 family, polypeptide A10 (Ugt1a10), mRNA.	NM_201641.1	0.44321	
TLR2	Mus musculus toll-like receptor 2 (Tlr2), mRNA.	NM_011905.2	0.44374	
PIRA11	Mus musculus paired-Ig-like receptor A11 (Pira11), mRNA.	NM_011088.1	0.44432	
AATK	Mus musculus apoptosis-associated tyrosine kinase (Aatk), mRNA.	NM_007377.3	0.44451	

GPR23	Mus musculus G protein-coupled receptor 23 (Gpr23), mRNA.	NM_175271.2	0.44472	*
2310006J04RIK	Mus musculus ankyrin repeat domain 37 (Ankrd37), mRNA.	NM_001039562.1	0.4462	*
SLC8A1	Mus musculus solute carrier family 8, member 1 (Slc8a1) mRNA.	NM_011406.1	0.44715	
ENTPD2	Mus musculus ectonucleoside triphosphate diphosphohydrolase 2 (Entpd2), mRNA.	NM_009849.1	0.44719	*
LY6E	Mus musculus lymphocyte antigen 6 complex, locus E (Ly6e), mRNA.	NM_008529.2	0.4483	
COL1A2	Mus musculus collagen, type I, alpha 2 (Col1a2), mRNA.	NM_007743.2	0.44881	
STAT1	Mus musculus signal transducer and activator of transcription 1 (Stat1), mRNA.	NM_009283.3	0.44883	
ANPEP	Mus musculus alanyl (membrane) aminopeptidase (Anpep), mRNA.	NM_008486.1	0.44893	
CRIM2	Mus musculus cysteine rich BMP regulator 2 (chordin like) (Crim2), mRNA.	NM_001029985.1	0.45255	
PALMD	Mus musculus palmdelphin (Palmd), mRNA.	NM_023245.3	0.45351	
COL6A1	Mus musculus procollagen, type VI, alpha 1 (Col6a1), mRNA.	NM_009933.2	0.45375	
ATF3	Mus musculus activating transcription factor 3 (Atf3), mRNA.	NM_007498.2	0.45377	
EG630499	Mus musculus predicted gene, EG630499 (EG630499), mRNA.	NM_001081015.1	0.45424	
CDK2	Mus musculus cyclin-dependent kinase 2 (Cdk2), transcript variant 1, mRNA.	NM_183417.2	0.45534	
POLD1	Mus musculus polymerase (DNA directed), delta 1, catalytic subunit (Pold1), mRNA.	NM_011131.2	0.45547	
RGMA	Mus musculus RGM domain family, member A (Rgma), mRNA.	NM_177740.4	0.45559	
UBTD2	Mus musculus ubiquitin domain containing 2 (Ubtd2), mRNA.	NM_173784.3	0.45603	
GNG2	Mus musculus guanine nucleotide binding protein (G protein), gamma 2 (Gng2), transcript variant 2, mRNA.	NM_001038637.1	0.45677	
IDH2	Mus musculus isocitrate dehydrogenase 2 (NADP+), mitochondrial (Idh2), nuclear gene encoding mitochondrial protein, mRNA.	NM_173011.1	0.45765	
ARHGEF3	Mus musculus Rho guanine nucleotide exchange factor (GEF) 3 (Arhgef3), mRNA.	NM_027871.1	0.45841	
EGR1	Mus musculus early growth response 1 (Egr1), mRNA.	NM_007913.5	0.46033	
PIP5K2A	Mus musculus phosphatidylinositol-5-phosphate 4-kinase, type II, alpha (Pip4k2a), mRNA.	NM_008845.4	0.46102	
H3F3A	Mus musculus H3 histone, family 3A (H3f3a), mRNA.	NM_008210.2	0.4611	
LOC434858	Mus musculus predicted gene, EG434858 (EG434858), non-coding RNA.	NR_002883.1	0.46112	
TLE4	Mus musculus transducin-like enhancer of split 4, homolog of Drosophila E(spl) (Tle4), mRNA. XM_924093 XM_924097 XM_924099 XM_924101 XM_924105 XM_924108 XM_924112 XM_924118	NM_011600.2	0.46113	
BTG1	PREDICTED: Mus musculus similar to myocardial vascular inhibition factor (LOC100047353), mRNA.	XM_001477963.1	0.46118	
JMJD2B	Mus musculus jumonji domain containing 2B (Jmjd2b), mRNA.	NM_172132.1	0.46147	
ADCY2	Mus musculus adenylate cyclase 2 (Adcy2), mRNA.	NM_153534.2	0.46347	
PROS1	Mus musculus protein S (alpha) (Pros1), mRNA.	NM_011173.2	0.46376	
PTGDS2	Mus musculus prostaglandin D2 synthase 2, hematopoietic (Ptgds2), mRNA.	NM_019455.2	0.46545	
CAR13	Mus musculus carbonic anhydrase 13 (Car13), mRNA.	NM_024495.4	0.46565	
BC004728		NM_174992.2	0.46638	
ITGA11	Mus musculus integrin alpha 11 (Itga11), mRNA.	NM_176922.4 NM_176922.4	0.46692 0.48869	
IRF5	Mus musculus interferon regulatory factor 5 (Irf5), mRNA.	NM_012057.3	0.46711	
GLIPR2	Mus musculus GLI pathogenesis-related 2 (Glipr2), mRNA.	NM_027450.3	0.46797	
SNX24	Mus musculus sorting nexing 24 (Snx24), mRNA.	NM_029394.3	0.46839	
COL14A1	Mus musculus collagen, type XIV, alpha 1 (Col14a1), mRNA.	NM_181277.2	0.46846	
BST2	Mus musculus bone marrow stromal cell antigen 2 (Bst2), mRNA.	NM_198095.2	0.4693	

CASP4	Mus musculus caspase 4, apoptosis-related cysteine peptidase (Casp4), mRNA.	NM_007609.1	0.47004	
MBP	Mus musculus myelin basic protein (Mbp), transcript variant 8, mRNA.	NM_0010252 45.1	0.47054	
SKAP2	Mus musculus src family associated phosphoprotein 2 (Skap2), mRNA.	NM_018773.2	0.471	
NAV1	Mus musculus neuron navigator 1 (Nav1), mRNA.	NM_173437.1	0.47124	
GM885	Mus musculus gene model 885, (NCBI) (Gm885), mRNA.	NM_0010334 35.1	0.4713	
CCL12	PREDICTED: Mus musculus similar to monocyte chemoattractant protein-5, transcript variant 1 (LOC100048556), mRNA.	XM_00148089 1.1	0.47144	
EGR2	Mus musculus early growth response 2 (Egr2), mRNA.	NM_010118.1	0.4715	
RHOE	Mus musculus Rho family GTPase 3 (Rnd3), mRNA.	NM_028810.2	0.47243	
FCNA	Mus musculus ficolin A (Fcna), mRNA.	NM_007995.3	0.47271	
MCC	Mus musculus mutated in colorectal cancers (Mcc), mRNA.	NM_0010334 06.1	0.47357	
FBLIM1	Mus musculus filamin binding LIM protein 1 (Fblim1), mRNA.	NM_133754.3	0.4738	
GNGT2	Mus musculus guanine nucleotide binding protein (G protein), gamma transducing activity polypeptide 2 (Gngt2), transcript variant 2, mRNA.	NM_0010386 64.2	0.47384	
MMP3	Mus musculus matrix metallopeptidase 3 (Mmp3), mRNA.	NM_010809.1	0.47405	
CLU	PREDICTED: Mus musculus similar to clusterin (LOC100046120), mRNA.	XM_00147561 1.1	0.47421	*
D730019B10 RIK	Mus musculus zinc finger CCCH type containing 12D (Zc3h12d), mRNA.	NM_172785.2	0.47438	
EG433016	Mus musculus predicted gene, EG433016 (EG433016), mRNA.	NM_0010825 47.1	0.4744	
PRKCDBP	Mus musculus protein kinase C, delta binding protein (Prkcdbp), mRNA.	NM_028444.1	0.47443	
ST5	Mus musculus suppression of tumorigenicity 5 (St5), transcript variant 1, mRNA.	NM_0010013 26.1 NM_029811.2	0.47471 0.49405	
PRICKLE1	Mus musculus prickle like 1 (Drosophila) (Prickle1), mRNA.	NM_0010332 17.3	0.47491	
TPM3	Mus musculus tropomyosin 3, gamma (Tpm3), mRNA.	NM_022314.2	0.47569	
IL7R	Mus musculus interleukin 7 receptor (Il7r), mRNA.	NM_008372.3	0.47571	
RAET1B	Mus musculus retinoic acid early transcript beta (Raet1b), mRNA.	NM_009017.1	0.47675	
SAA3	Mus musculus serum amyloid A 3 (Saa3), mRNA.	NM_011315.3	0.47694	
GSTK1	Mus musculus glutathione S-transferase kappa 1 (Gstk1), mRNA.	NM_029555.2	0.47789	
UGCG	Mus musculus UDP-glucose ceramide glucosyltransferase (Ugcg), mRNA.	NM_011673.2	0.47796	
AP2B1	Mus musculus adaptor-related protein complex 2, beta 1 subunit (Ap2b1), transcript variant 1, mRNA.	NM_0010358 54.2	0.47975	
BC003277	Mus musculus transmembrane protein 51 (Tmem51), mRNA.	NM_145402.3	0.48	
BZW2	Mus musculus basic leucine zipper and W2 domains 2 (Bzw2), mRNA.	NM_025840.2	0.48078	
2010005H15 RIK	Mus musculus RIKEN cDNA 2010005H15 gene (2010005H15Rik), mRNA.	NM_029733.2	0.48083	
ZCCHC5	Mus musculus zinc finger, CCHC domain containing 5 (Zcchc5), mRNA.	NM_199468.1	0.48132	*
5133400G04 RIK	Mus musculus RIKEN cDNA 5133400G04 gene (5133400G04Rik), transcript variant 2, mRNA.	NM_029485.1	0.48307	
5133400G04 RIK	Mus musculus RIKEN cDNA 5133400G04 gene (5133400G04Rik), transcript variant 2, mRNA.	NM_029485.1	0.48331	
CDC7	Mus musculus cell division cycle 7 (S. cerevisiae) (Cdc7), mRNA.	NM_009863.2	0.48349	
TM6SF1	Mus musculus transmembrane 6 superfamily member 1 (Tm6sf1), mRNA.	NM_145375.1	0.48433	
BAT1A	Mus musculus HLA-B-associated transcript 1A (Bat1a), mRNA.	NM_019693.2	0.48514	
PNP	PREDICTED: Mus musculus similar to purine nucleoside phosphorylase (LOC100045567), mRNA.	XM_00147453 6.1	0.48626	
MFAP4	Mus musculus microfibrillar-associated protein 4 (Mfap4), mRNA.	NM_029568.2	0.48718	

ENO1	Mus musculus enolase 1, alpha non-neuron (Eno1), mRNA.	NM_023119.1	0.4872	*
OSMR	Mus musculus oncostatin M receptor (Osmr), mRNA.	NM_011019.1	0.48807	
TNFAIP8L2	Mus musculus tumor necrosis factor, alpha-induced protein 8-like 2 (Tnfaip8l2), mRNA.	NM_027206.1	0.48985	
PDZRN3	Mus musculus PDZ domain containing RING finger 3 (Pdzrn3), mRNA.	NM_018884.1	0.48992	
TRPV2	Mus musculus transient receptor potential cation channel, subfamily V, member 2 (Trpv2), mRNA.	NM_011706.1	0.49065	
WSB1	Mus musculus WD repeat and SOCS box-containing 1 (Wsb1), transcript variant 2, mRNA.	NM_0010425 65.2	0.49111	
STAB1	Mus musculus stabilin 1 (Stab1), mRNA.	NM_138672.2	0.49287	
ALDOA	Mus musculus aldolase A, fructose-bisphosphate (Aldoa), mRNA.	NM_007438.3	0.49416	*
IL1RN	Mus musculus interleukin 1 receptor antagonist (Il1rn), transcript variant 1, mRNA.	NM_031167.3	0.49519	
SNX2	Mus musculus sorting nexin 2 (Snx2), mRNA.	NM_026386.1	0.49573	
SSR2	Mus musculus signal sequence receptor, beta (Ssr2), mRNA.	NM_025448.2	0.49652	
2810046M22 RIK		NM_026621.1	0.49761	*
ZFP608	Mus musculus zinc finger protein 608 (Zfp608), mRNA. XM_001000874 XM_001000888 XM_001000902 XM_001000914 XM_993937 XM_993952 XM_993993 XM_994038 XM_994057 XM_994076	NM_175751.3	0.49809	
TPI1	Mus musculus triosephosphate isomerase 1 (Tpi1), mRNA.	NM_009415.1	0.49815	
RAB32	Mus musculus RAB32, member RAS oncogene family (Rab32), mRNA.	NM_026405.3	0.49837	
CSNK1D	Mus musculus casein kinase 1, delta (Csnk1d), transcript variant 2, mRNA.	NM_027874.2	0.49875	
TUBA1A	Mus musculus tubulin, alpha 1A (Tuba1a), mRNA.	NM_011653.1	0.49881	
PAOX	Mus musculus polyamine oxidase (exo-N4-amino) (Paox), mRNA.	NM_153783.2	0.49926	
