# Supplementary material for: The Role of Parathyroid Hormone-Related Protein (PTHrP) in Osteoblast Response to Microgravity: Mechanistic Implications for Osteoporosis Development
Source: PLoS One. 2016 Jul 27;11(7):e0160034. doi: 10.1371/journal.pone.0160034 (PMC4963112; doi:10.1371/journal.pone.0160034)
Supplement: S4 Table — (A) Genes (total 102) upregulated by intermittent PTHrP1-36 treatment. Fold change > 1.5, p < 0.05. (B) Genes (total 61) downregulated by treatment. Fold changes < 0.66, p < 0.05. (PDF) [file pone.0160034.s005.pdf]

S4 A and B tables: Genes (163) affected by PTHrP$_{1-36}$ treatment.

S4 A Table: Genes (102) upregulated by PTHrP $_{1-36}$ treatment. Fold change > 1.5, p value< 0.05

Target ID	Definition (all probes: *Mus musculus*)	RefSeq ID	Fold change	Down regulated by both 0g and ablation
RBM3	PREDICTED: similar to RNA binding motif protein 3 (LOC100043257), mRNA.	XM_001480197.1	3.3167	*
HIST1H2AD	histone cluster 1, H2ad (Hist1h2ad), mRNA.	NM_178188.3	3.0702	*
HIST1H2AH	histone cluster 1, H2ah (Hist1h2ah), mRNA.	NM_175659.1	2.6444	*
HIST1H2AN	histone cluster 1, H2an (Hist1h2an), mRNA.	NM_178184.1	2.6297	*
HIST1H2AK	histone cluster 1, H2ak (Hist1h2ak), mRNA.	NM_178183.1	2.6191	*
PTN	pleiotrophin (Ptn), mRNA.	NM_008973.2	2.6102	*
HIST1H2AF	histone cluster 1, H2af (Hist1h2af), mRNA.	NM_175661.1	2.5906	*
HIST1H2AO	histone cluster 1, H2ao (Hist1h2ao), mRNA.	NM_178185.1	2.3636	*
CDCA3	cell division cycle associated 3 (Cdca3), mRNA.	NM_013538.4	2.2954	
2310061N23RIK	interferon, alpha-inducible protein 27 (Ifi27), mRNA.	NM_029803.1	2.24	*
CDC20	cell division cycle 20 homolog (S. cerevisiae) (Cdc20), mRNA.	NM_023223.1<br>NM_023223.1<br>NM_023223.1	2.2371<br>1.6896<br>1.6657	
CENPA	centromere protein A (Cenpa), mRNA.	NM_007681.2	2.2172	*
HIST1H2AG	histone cluster 1, H2ag (Hist1h2ag), mRNA.	NM_178186.2	2.186	*
LUM	lumican (Lum), mRNA.	NM_008524.1	2.1278	*
BIRC5	baculoviral IAP repeat-containing 5 (Birc5), transcript variant 1, mRNA.	NM_009689.1<br>NM_009689.2<br>NM_009689.2	2.0974<br>1.9794<br>1.7963	
ITM2A	integral membrane protein 2A (Itm2a), mRNA.	NM_008409.2	2.0722	*
IGFBP5	insulin-like growth factor binding protein 5 (Igfbp5), mRNA.	NM_010518.2	2.0686	*
DCN	decorin (Dcn), mRNA.	NM_007833.4<br>NM_007833.4	2.0663<br>1.7317	*
D0H4S114	DNA segment, human D4S114 (D0H4S114), mRNA.	NM_053078.3	2.0484	
HAGHL	hydroxyacylglutathione hydrolase-ike (Haghl) mRNA		2.0424	
PLK1	polo-like kinase 1 (Drosophila) (Plk1), mRNA.	NM_011121.3	2.0252	
KIF22	kinesin family member 22 (Kif22), mRNA.	NM_145588.1	1.9758	
2700094K13RIK	RIKEN cDNA 2700094K13 gene (2700094K13Rik), transcript variant 2, mRNA.	NM_001037279.1	1.931	
HIST2H2AC	histone cluster 2, H2ac (Hist2h2ac), mRNA.	NM_175662.1	1.8885	
1500015O10RIK	RIKEN cDNA 1500015O10 gene (1500015O10Rik), mRNA.	NM_024283.2	1.8607	*
ANLN	anillin, actin binding protein (Anln), mRNA.	NM_028390.2	1.858	
NUSAP1	nucleolar and spindle associated protein 1 (Nusap1), transcript variant 2, mRNA.	NM_001042652.1	1.856	
PRC1	protein regulator of cytokinesis 1 (Prc1), mRNA.	NM_145150.1	1.8457	
IDH2	isocitrate dehydrogenase 2 (NADP+), mitochondrial (Idh2), nuclear gene encoding mitochondrial protein, mRNA.	NM_173011.1	1.8455	
MFAP2	microfibrillar-associated protein 2 (Mfap2), mRNA.	NM_008546.2<br>NM_008546.2<br>NM_008546.2	1.8433<br>1.5754<br>1.5018	*
TK1	thymidine kinase 1 (Tk1), mRNA.	NM_009387.1	1.8403	
ALOX5AP	arachidonate 5-lipoxygenase activating protein (Alox5ap), mRNA.	NM_009663.1	1.8329	
UHRF1	ubiquitin-like, containing PHD and RING finger domains, 1 (Uhrf1), mRNA.	NM_010931.2	1.8282	
MCM5	minichromosome maintenance deficient 5, cell division cycle 46 (S. cerevisiae) (Mcm5), mRNA.	NM_008566.2	1.8226	
CDKN1C	cyclin-dependent kinase inhibitor 1C (P57) (Cdkn1c), mRNA.	NM_009876.3	1.7975	
5133400G04RIK	RIKEN cDNA 5133400G04 gene (5133400G04Rik), transcript variant 2, mRNA.	NM_029485.1	1.7715	
ELMO1	engulfment and cell motility 1, ced-12 homolog (C. elegans) (Elmo1), transcript variant 2, mRNA.	NM_198093.2	1.731	

GPR23	G protein-coupled receptor 23 (Gpr23), mRNA.	NM_175271.2	1.7145	
SLAMF9	SLAM family member 9 (Slamf9), mRNA.	NM_029612.3	1.71	
STK6	aurora kinase A (Aurka), mRNA.	NM_011497.3	1.6983	
H2AFZ	H2A histone family, member Z (H2afz), mRNA.	NM_016750.1	1.6962	
CAPN6	calpain 6 (Capn6), mRNA.	NM_007603.2	1.6952	
CD52	CD52 antigen (Cd52), mRNA.	NM_013706.1	1.6884	*
C1QA	complement component 1, q subcomponent, alpha polypeptide (C1qa), mRNA.	NM_007572.2	1.6858	*
CTSC	cathepsin C (Ctsc), mRNA.	NM_009982.2	1.6839	*
ALDH3B1	aldehyde dehydrogenase 3 family, member B1 (Aldh3b1), mRNA.	NM_026316.2	1.678	*
OGN	osteoglycin (Ogn), mRNA.	NM_008760.2	1.6716	
TGFBI	transforming growth factor, beta induced (Tgfbi), mRNA.	NM_009369.1	1.6641	*
FMO1	flavin containing monooxygenase 1 (Fmo1), mRNA.	NM_010231.2	1.6578	*
IGF2	insulin-like growth factor 2 (Igf2), mRNA.	NM_010514.2	1.6563	*
CORO1A	coronin, actin binding protein 1A (Coro1a), mRNA.	NM_009898.2<br>NM_009898.2<br>NM_009898.2	1.6563<br>1.6294<br>1.6205	
MCM6	minichromosome maintenance deficient 6 (MIS5 homolog, S. pombe) (S. cerevisiae) (Mcm6), mRNA.	NM_008567.1	1.6556	
SPAG5	sperm associated antigen 5 (Spag5), mRNA.	NM_017407.1	1.6556	
CYP7B1	cytochrome P450, family 7, subfamily b, polypeptide 1 (Cyp7b1), mRNA.	NM_007825.3	1.6527	*
NRM	nurim (nuclear envelope membrane protein) (Nrm), mRNA.	NM_134122.2	1.6419	
CDCA2	cell division cycle associated 2 (Cdca2), mRNA.	NM_175384.3	1.6403	
CRABP1	cellular retinoic acid binding protein I (Crabp1), mRNA.	NM_013496.2	1.6401	
TYMS	thymidylate synthase (Tyms), mRNA.	NM_021288.3	1.6377	
CXCL12	chemokine (C-X-C motif) ligand 12 (Cxcl12) mRNA.	NM_013655.2	1.6375	*
LDB2	LIM domain-binding 2 (Ldb2) mRNA.	NM_010698.2	1.635	*
EMR1	EGF-like module containing, mucin-like, hormone receptor-like sequence 1 (Emr1), mRNA.	NM_010130.3	1.6262	*
P2RY6	pyrimidinergic receptor P2Y, G-protein coupled, 6 (P2ry6), mRNA.	NM_183168.1	1.6111	
4632417K18RIK	RIKEN cDNA 4632417K18 gene (4632417K18Rik), mRNA.	NM_026640.2	1.606	
LRRC15	leucine-rich repeat containing 15 (Lrrc15) mRNA.	XM_358823.1	1.6042	
KNG1	kininogen 1 (Kng1), mRNA.	NM_023125.2	1.5999	*
OLFML1	olfactomedin-like 1 (Olfml1), mRNA.	NM_172907.2	1.5997	*
PBK	PDZ binding kinase (Pbk), mRNA.	NM_023209.1	1.5995	
AOC3	amine oxidase, copper containing 3 (Aoc3), mRNA.	NM_009675.1	1.5983	*
GMFG	glia maturation factor, gamma (Gmfg), transcript variant 1, mRNA.	NM_022024.2<br>NM_022024.2	1.5966<br>1.5325	*
NCF4	neutrophil cytosolic factor 4 (Ncf4), mRNA.	NM_008677.1	1.5936	*
SFRP2	secreted frizzled-related protein 2 (Sfrp2), mRNA.	NM_009144.1	1.5892	*
CSRP2	cysteine and glycine-rich protein 2 (Csrp2), mRNA.	NM_007792.3	1.5844	
SMPDL3B	sphingomyelin phosphodiesterase, acid-like 3B (Smpdl3b), mRNA.	NM_133888.2	1.582	
SMARCA1	SWI/SNF related, matrix associated, actin dependent regulator of chromatin, subfamily a, member 1 (Smarca1), mRNA.	NM_053123.3	1.5706	
2810417H13RIK	RIKEN cDNA 2810417H13 gene (2810417H13Rik), mRNA.	NM_026515.2	1.5684	
CDC2A	cell division cycle 2 homolog A (S. pombe) (Cdc2a), mRNA.	NM_007659.3	1.5683	
SIRPB1	signal-regulatory protein beta 1 (Sirpb1), transcript variant 3, mRNA.	NM_001002898.1	1.5595	*
RASSF4	Ras association (RalGDS/AF-6) domain family member 4 (Rassf4), mRNA.	NM_178045.3<br>NM_178045.3	1.5588<br>1.5215	*
COL1A2	collagen, type I, alpha 2 (Col1a2), mRNA.	NM_007743.2	1.5571	
KCNAB2	potassium voltage-gated channel, shaker-related subfamily, beta member 2 (Kcnab2), mRNA.	NM_010598.2	1.5566	*
LOC434858	predicted gene, EG434858 (EG434858), non-coding RNA.	NR_002883.1	1.556	
LSP1	PREDICTED: predicted gene, ENSMUSG00000043795 (ENSMUSG00000043795), mRNA.	XM_001480835.1	1.5468	
ARL11	ADP-ribosylation factor-like 11 (Arl11), mRNA.	NM_177337.3	1.544	
CX3CR1	chemokine (C-X3-C) receptor 1 (Cx3cr1) mRNA.	NM_009987.2	1.5435	
FOLR2	folate receptor 2 (fetal) (Folr2), mRNA.	NM_008035.1	1.5354	
5133400G04RIK	RIKEN cDNA 5133400G04 gene (5133400G04Rik), transcript variant 2, mRNA.	NM_029485.1	1.5328	
COL3A1	collagen, type III, alpha 1 (Col3a1), mRNA.	NM_009930.1	1.5317	*
CCNB1	cyclin B1 (Ccnb1), mRNA.	NM_172301.3	1.5308	

HMGN2	high mobility group nucleosomal binding domain 2 (Hmgn2), mRNA.	NM_016957.3	1.5306	
5830416A07RIK	zinc finger CCCH-type containing 18 (Zc3h18), transcript variant 1, mRNA.	NM_001029993.1	1.5283	
VAV1	vav 1 oncogene (Vav1), mRNA.	NM_011691.3	1.5251	
POLD1	polymerase (DNA directed), delta 1, catalytic subunit (Pold1), mRNA.	NM_011131.2	1.5233	
CHAF1B	chromatin assembly factor 1, subunit B (p60) (Chaf1b), mRNA.	NM_028083.3	1.5227	
SRPX	sushi-repeat-containing protein (Srpx), mRNA.	NM_016911.4	1.5225	*
DAB2	disabled 2 mitogen-responsive phosphoprotein (Dab2) mRNA	NM_023118.1	1.5198	*
MDK	midkine (Mdk), transcript variant 1, mRNA.	NM_010784.4	1.5185	
EVI2A	ecotropic viral integration site 2a (Evi2a), transcript variant 2, mRNA.	NM_010161.3	1.5179	*
6430706D22RIK	RIKEN cDNA 6430706D22 gene (6430706D22Rik), mRNA.	NM_198652.1	1.5165	
PLCG2	phospholipase C, gamma 2 (Plcg2), mRNA.	NM_172285.1	1.5154	
NID2	nidogen 2 (Nid2), mRNA.	NM_008695.2	1.5066	*
1810009M01RIK	transmembrane protein 176B (Tmem176b), mRNA.	NM_023056.3	1.5054	
C1QB	complement component 1, q subcomponent, beta polypeptide (C1qb), mRNA.	NM_009777.2	1.5032	*
IL1RL1	interleukin 1 receptor-like 1 (Il1rl1), transcript variant 2, mRNA.	NM_010743.1	1.5023	

S4 A Table: Genes (61) downregulated by PTHrP $_{1\text{-}36}$ treatment. Fold change <0.65 , p value< 0.05

Target ID	Definition (all probes: *Mus musculus*)	RefSeq ID	Fold change	Up regulated by both 0g and ablation
AQP5	aquaporin 5 (Aqp5), mRNA.<br>similar to aquaporin 5 (LOC100046616), mRNA.	NM_009701.4<br>XM_001476512.1	0.3705<br>0.4987	*
FOSB	FBJ osteosarcoma oncogene B (Fosb), mRNA.	NM_008036.2	0.4476	
TNFRSF11B	tumor necrosis factor receptor superfamily, member 11b (osteoprotegerin) (Tnfrsf11b), mRNA.	NM_008764.3	0.4687	*
HSPB1	heat shock protein 1 (Hspb1), mRNA.	NM_013560.1	0.4871	*
MRPPLF3	prolactin family 2, subfamily c, member 4 (Prl2c4), mRNA.	NM_011954.2	0.5052	*
NKD2	naked cuticle 2 homolog (Drosophila) (Nkd2), mRNA.	NM_028186.3	0.515	*
AI450948	AHNAK nucleoprotein 2 (Ahnak2), mRNA.	NM_001033476.1	0.5172	
PLF2	prolactin family 2, subfamily c, member 3 (Prl2c3), mRNA.	NM_011118.1	0.5191	*
BDNF	brain derived neurotrophic factor (Bdnf), transcript variant 3, mRNA.	NM_001048141.1	0.5335	*
TIMP3	tissue inhibitor of metalloproteinase 3 (Timp3), mRNA.	NM_011595.2<br>NM_011595.2	0.5338<br>0.6268	*
CRYAB	crystallin, alpha B (Cryab), mRNA.	NM_009964.1	0.5387	*
INHBB	PREDICTED: similar to Inhbb protein (LOC100046802), mRNA.	XM_001476835.1	0.5494	
ASS1	argininosuccinate synthetase 1 (Ass1), mRNA.	NM_007494.3	0.5595	*
ESD	esterase D/formylglutathione hydrolase (Esd), mRNA.	NM_016903.2	0.5693	*
ZFP36	zinc finger protein 36 (Zfp36), mRNA.	NM_011756.4	0.5767	
INHBA	inhibin beta-A (Inhba), mRNA.	NM_008380.1	0.5792	*
PRSS19	kallikrein related-peptidase 8 (Klk8), mRNA.	NM_008940.2	0.5806	
3110050N22RIK	family with sequence similarity 164, member A (Fam164a), mRNA.	NM_173181.3	0.5834	
CD44	CD44 antigen (Cd44), transcript variant 2, mRNA.	NM_001039150.1	0.5864	
JUNB	Jun-B oncogene (Junb), mRNA.	NM_008416.1	0.5893	
CDKN1A	cyclin-dependent kinase inhibitor 1A (P21) (Cdkn1a), mRNA.	NM_007669.2	0.592	
THBD	thrombomodulin (Thbd), mRNA.	NM_009378.2	0.5953	
GREM1	gremlin1 (Grem1) mRNA.	NM_011824.1	0.5979	*
GDF15	growth differentiation factor 15 (Gdf15), mRNA.	NM_011819.1	0.5986	*
MUSTN1	musculoskeletal, embryonic nuclear protein 1 (Mustn1), mRNA.	NM_181390.1	0.6016	*
MDM2	transformed mouse 3T3 cell double minute 2 (Mdm2), mRNA.	NM_010786.2	0.6034	*
ECM1	extracellular matrix protein 1 (Ecm1), mRNA.	NM_007899.1	0.6041	
DUSP1	dual specificity phosphatase 1 (Dusp1), mRNA.	NM_013642.2	0.609	
KCTD10	potassium channel tetramerisation domain containing 10 (Kctd10), mRNA.	NM_026145.3	0.6113	*
D4BWG0951E	DNA segment, Chr 4, Brigham & Women's Genetics 0951 expressed (D4Bwg0951e), mRNA.	NM_026821.4	0.613	

MYD116	myeloid differentiation primary response gene 116 (Myd116), mRNA.	NM_008654.1	0.6156	
RGS16	regulator of G-protein signaling 16 (Rgs16), mRNA.	NM_011267.2	0.6157	
OCIL	C-type lectin domain family 2, member d (Clec2d), mRNA.	NM_053109.2	0.6171	
SCMH1	sex comb on midleg homolog 1 (Scmh1), mRNA.	NM_013883.1	0.6175	
TRP53INP1	transformation related protein 53 inducible nuclear protein 1 (Trp53inp1), mRNA.	NM_021897.1	0.6187	
CCND1	cyclin D1 (Ccnd1), mRNA.	NM_007631.2	0.6205	
PHLDA1	pleckstrin homology-like domain, family A, member 1 (Phlda1), mRNA.	NM_009344.1	0.6228	
EEF1A2	eukaryotic translation elongation factor 1 alpha 2 (Eef1a2), mRNA.	NM_007906.2	0.6231	*
WHRN	whirlin (Whrn), transcript variant 3, mRNA.	NM_001008792.1	0.6237	
COL4A1	procollagen, type IV, alpha 1 (Col4a1), mRNA.	NM_009931.1	0.6247	
EPHX1	epoxide hydrolase 1, microsomal (Ephx1), mRNA.	NM_010145.2	0.6255	
ANXA8	annexin A8 (Anxa8), mRNA.	NM_013473.3	0.6258	
FOS	FBJ osteosarcoma oncogene (Fos), mRNA.	NM_010234.2	0.6262	
VEGFB	vascular endothelial growth factor B (Vegfb), mRNA.	NM_011697.2	0.6287	
UNC5B	unc-5 homolog B (C. elegans) (Unc5b), mRNA.	NM_029770.2	0.6295	
THBS1	thrombospondin 1 (Thbs1), mRNA.	NM_011580.3	0.6345	
HIST1H4H	histone cluster 1, H4h (Hist1h4h), mRNA.	NM_153173.2	0.635	
VAT1	vesicle amine transport protein 1 homolog (T californica) (Vat1), mRNA.	NM_012037.2	0.64	
BC065120	zinc finger, MIZ-type containing 1 (Zmiz1), mRNA.	NM_183208.2	0.6402	
E430002G05RIK	RIKEN cDNA E430002G05 gene (E430002G05Rik), mRNA.	NM_173749.3	0.6412	
STMN2	stathmin-like 2 (Stmn2), mRNA.	NM_025285.2	0.645	
PVRL2	poliovirus receptor-related 2 (Pvrl2), mRNA.	NM_008990.2	0.6454	
3110050N22RIK	family with sequence similarity 164, member A (Fam164a), mRNA.	NM_173181.1	0.6461	
PHF17	PHD finger protein 17 (Phf17), mRNA.	NM_172303.3	0.6468	
SLC4A2	solute carrier family 4 (anion exchanger), member 2 (Slc4a2), mRNA.	NM_009207.2	0.6499	
HSP105	heat shock protein 105 (Hsp105) mRNA.	NM_013559.1	0.652	
CYR61	cysteine rich protein 61 (Cyr61), mRNA.	NM_010516.1	0.6523	
SLC39A6	solute carrier family 39 (metal ion transporter), member 6 (Slc39a6), mRNA.	NM_139143.2	0.6535	
SCX	scleraxis (Scx), mRNA.	NM_198885.2	0.6554	*
BAG3	BCL2-associated athanogene 3 (Bag3), mRNA.	NM_013863.4	0.6559	
SCA2	ataxin 2 (Atxn2), mRNA.	NM_009125.2	0.657	
PKIA	protein kinase inhibitor, alpha (Pkia), mRNA.	NM_008862.3	0.6583	
